# Finding REMO: a sequencing method for recognition-encoded melamine oligomers

**DOI:** 10.1039/d6sc05680f

**Published:** 2026-07-29

**Authors:** Ben Iddon, Pawel H. Grab, Joseph T. Smith, Oliver N. Evans, Anca-Luiza Cotîrlan, Christopher A. Hunter

**Affiliations:** a Yusuf Hamied Department of Chemistry, University of Cambridge Lensfield Road Cambridge CB2 1EW UK herchelsmith.orgchem@ch.cam.ac.uk

## Abstract

Recognition-encoded melamine oligomers (REMO) are synthetic polymers based on an alternating triazine–piperazine backbone equipped with side chains that promote sequence-selective formation of duplexes and provide a basis for molecular replication *via* template-directed synthesis. Here we report the development of a method for determining the sequence of a REMO using a backbone fragmentation strategy. Methyl iodide was used to selectively methylate nitrogen atoms on the backbone without alkylating sites on the side chains. Nitrogen quaternisation generated electrophilic sites on the backbone that underwent aminolysis in methylamine solution. Each chain cleavage reaction generated three different fragments that were detectable by LCMS. This two-step fragmentation procedure was verified as a viable sequencing method for REMO of different length equipped with a variety of different side chains. The sequencing method was also used to investigate the fidelity of a replication process using base-filling with a mixed sequence REMO template.

## Abstract 2

Recognition-encoded melamine oligomers (REMO) are synthetic polymers with interesting sequence-dependent properties. We report a sequencing method for REMO based on chemical fragmentation and use it to assess the fidelity of a copying experiment.

## Graphical abstract

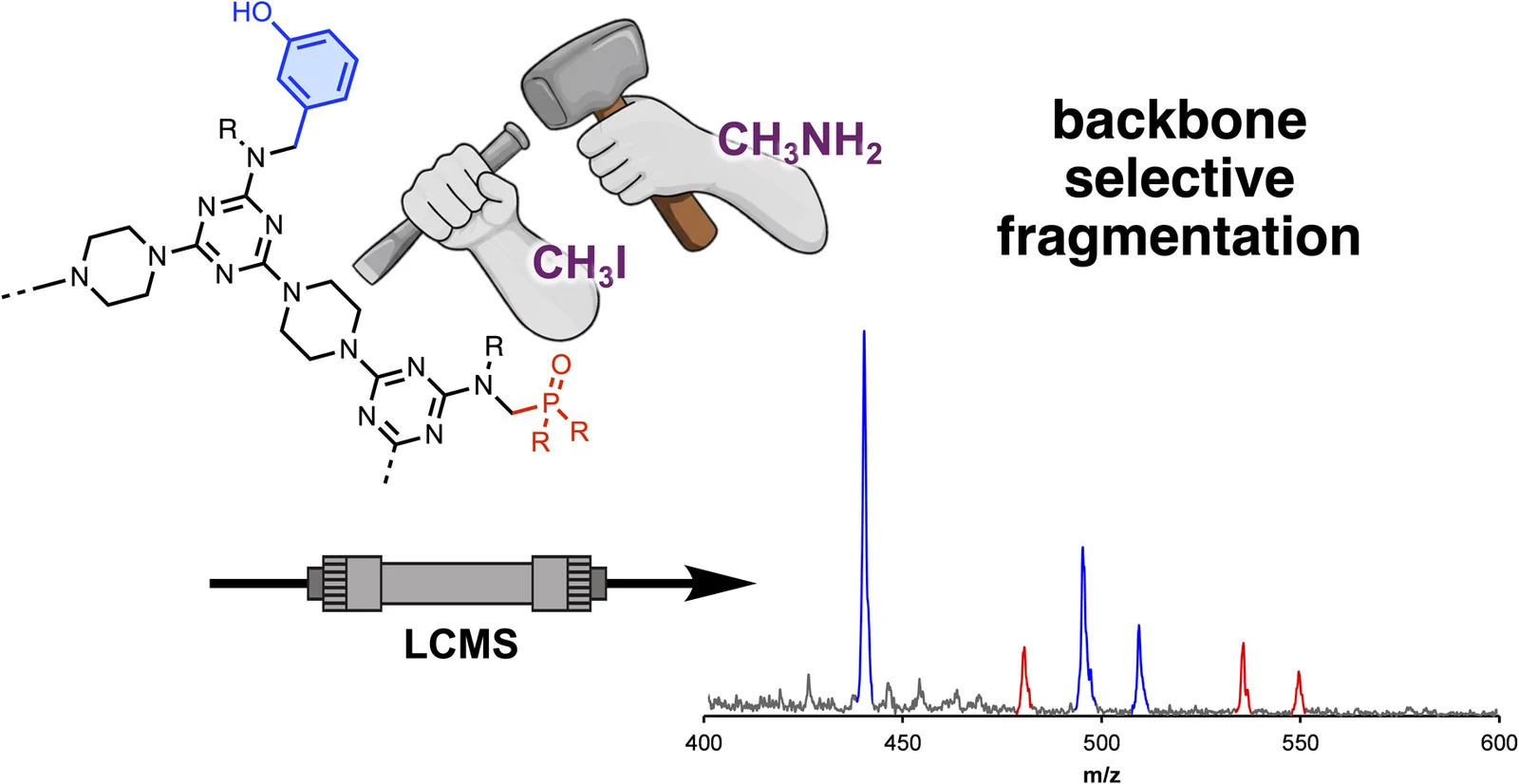

## Introduction

Nature achieves highly optimised functions with a relatively small set of building blocks by exploiting monodisperse recognition-encoded polymers. The precise arrangement of monomers determines the three-dimensional structure of the folded polymer which in turn determines the functional properties.^1^ Synthetic sequence-controlled macromolecules are emerging as potential tools for achieving similar functionalities in non-biological contexts.^2–9^ Recognition-encoded melamine oligomers (REMO) are synthetic polymers consisting of an alternating triazine–piperazine backbone equipped with side chains that carry H-bond recognition units or reactive handles. REMO can be synthesised with precise sequence-control using automated solid-phase synthesis,^10^ sequence-complementary strands cooperatively assemble into H-bonded duplexes,^11–15^ and single strands fold in a sequence-dependent manner.^11,16,17^ We have also reported progress towards replication of REMO *via* template-directed synthesis.^18,19^ REMO therefore represent a promising chemical system for the development of novel functional macromolecules that operate outside of biological conditions. However, new analytical tools are required for characterising these new sequence polymers, and here we report a structure determination method that can be used to decode the sequence of monomer building blocks in a REMO.

**Fig. 1:**
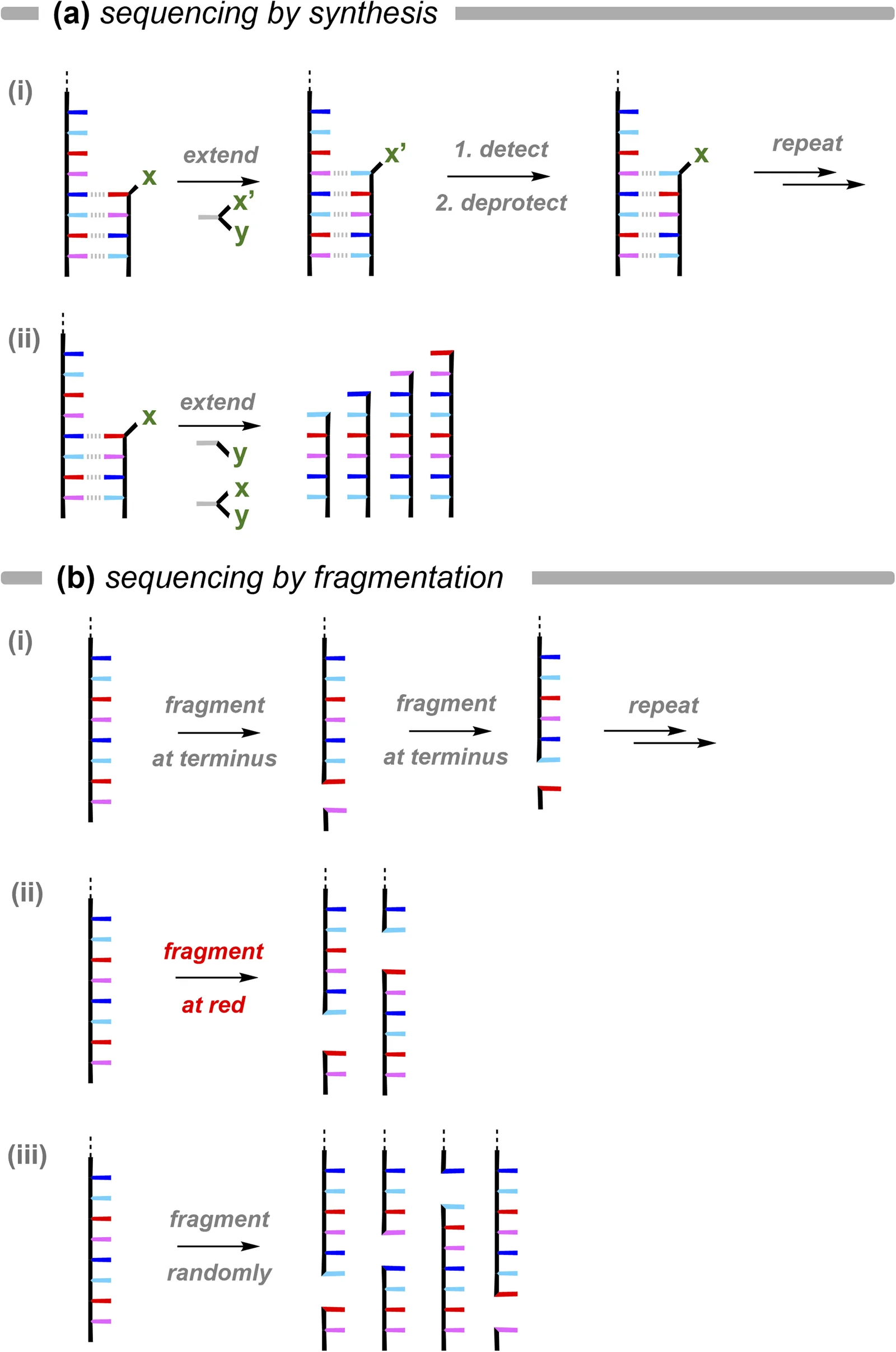
Polymer sequencing strategies. (a) Sequencing by synthesis: (i) replication with reversible termination (ii) replication with permanent termination (x and y are reactive groups and x′ is a protected x group). (b) Sequencing by fragmentation: (i) chain-end fragmentation (ii) monomer-selective fragmentation (iii) unselective fragmentation.

The methods that have been developed for sequencing polymers rely either on fragmentation of the chain or partial copying of the chain using template-directed synthesis.^20,21^ In both strategies, a series of truncated strands are detected, which allows the sequence of the polymer to be decoded. Synthesis-based sequencing methods are limited to polynucleotides, which are able undergo high-fidelity enzymatic replication (Fig. 1a). Sequence information is read either by using reversible terminators, which allow the sequential observation of replication intermediates,^22,23^ or by using permanent terminators, which truncate the copy strands.^24,25^ Fragmentation of polymers can be achieved by tandem mass-spectrometry or by chemical or enzymatic degradation (Fig. 1b). Tandem mass spectrometry has been used to sequence polypeptides, and is the most common method used for sequencing synthetic polymers.^26–36^ This method has the advantage that small amounts of low purity sample can be used, but the bonds connecting the backbone must be significantly weaker than the bonds connecting the side-chains. Alternatively, the polymer can be fragmented prior to analysis by mass spectrometry. The backbone can be cleaved selectively at certain monomers,^37^ selectively at the chain end,^38–45^ or unselectively at random positions on the chain.^46–51^ Other methods of sequencing include the use of nanopores,^52–54^ spectroscopic read-outs,^55–59^ or isotopic ratio encoding.^60^ Here we describe the development of a sequencing method for REMO based on chemical fragmentation of the backbone. The utility of the methodology is demonstrated by quantifying the fidelity of a template-directed synthesis experiment using base-filling on a mixed-sequence template.^19^

## Results and discussion

### Backbone fragmentation

**Fig. 2:**
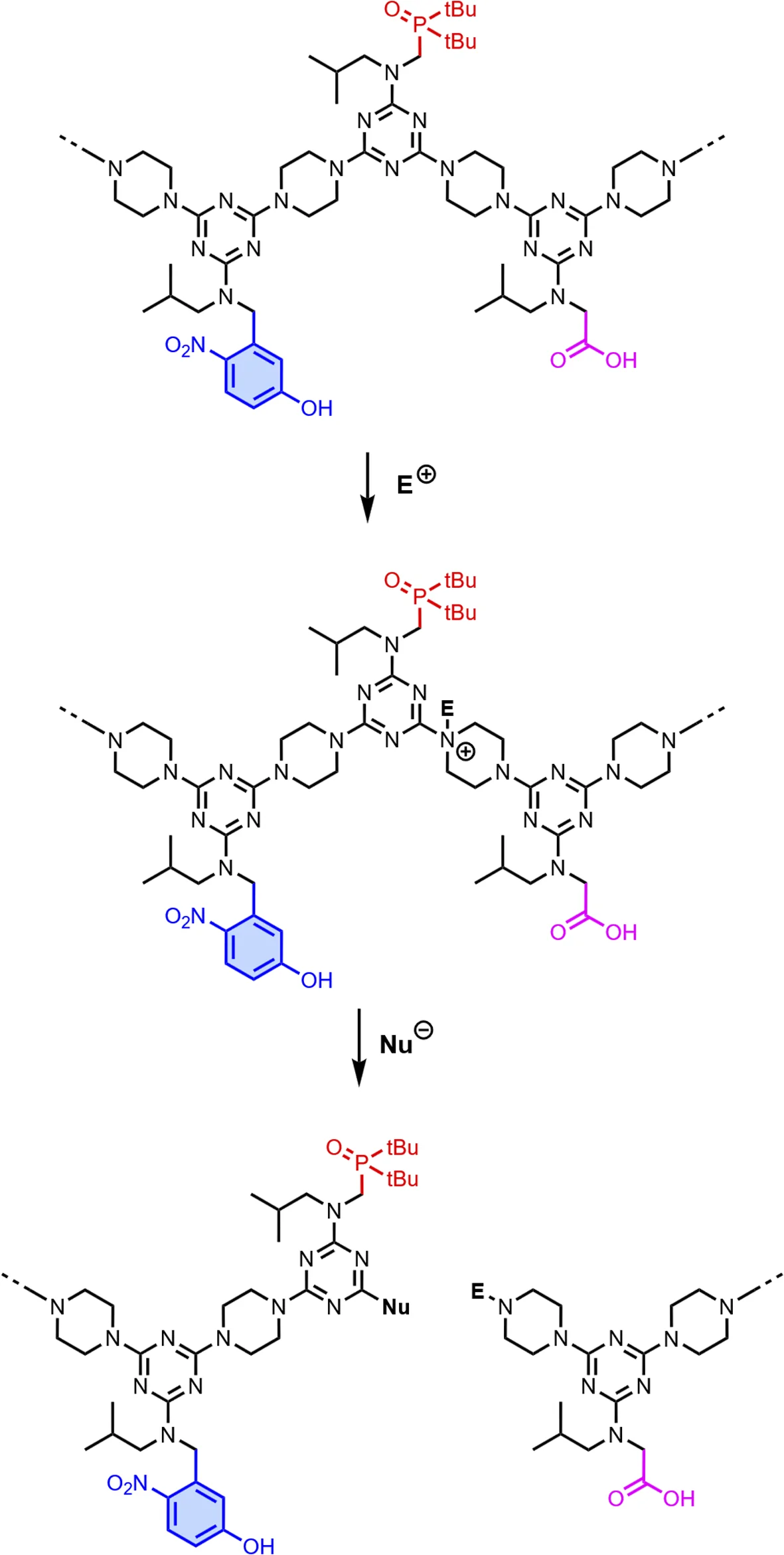
Chemical fragmentation of a REMO by selective reaction of an electrophile with a nitrogen in the backbone, activating the adjacent triazine towards nucleophilic attack.

Fig. 2 illustrates the chemical structure of a REMO. Whilst other synthetic polymers have been designed for sequencing, containing easily cleavable bonds in the backbone, REMO were instead designed to have interesting conformational properties.^11,16^ However, the chemical stability of the triazine–piperazine backbone makes common sequencing methods unsuitable for REMO – preliminary tandem MS experiments showed that the side chains are preferentially cleaved, erasing the sequence information (Fig. S33). The development of a method for selective chemical fragmentation of the backbone therefore appeared to be the most attractive option for REMO sequencing.^21,51^*N*-triazinylammonium salts are used as peptide coupling reagents and react with nucleophiles leading to cleavage of the exocyclic triazine–nitrogen bond.^61–63^ By analogy, we envisaged the triazine–piperazine bonds of the REMO backbone might be cleaved in a two-step process: alkylation of the piperazine to generate a *N*-triazinylammonium salt followed by nucleophilic substitution (Fig. 2). Compound 1 (2,4,6-tri-piperidinyl-1,3,5-triazine) was synthesised in order to investigate this process.

**Fig. 3:**
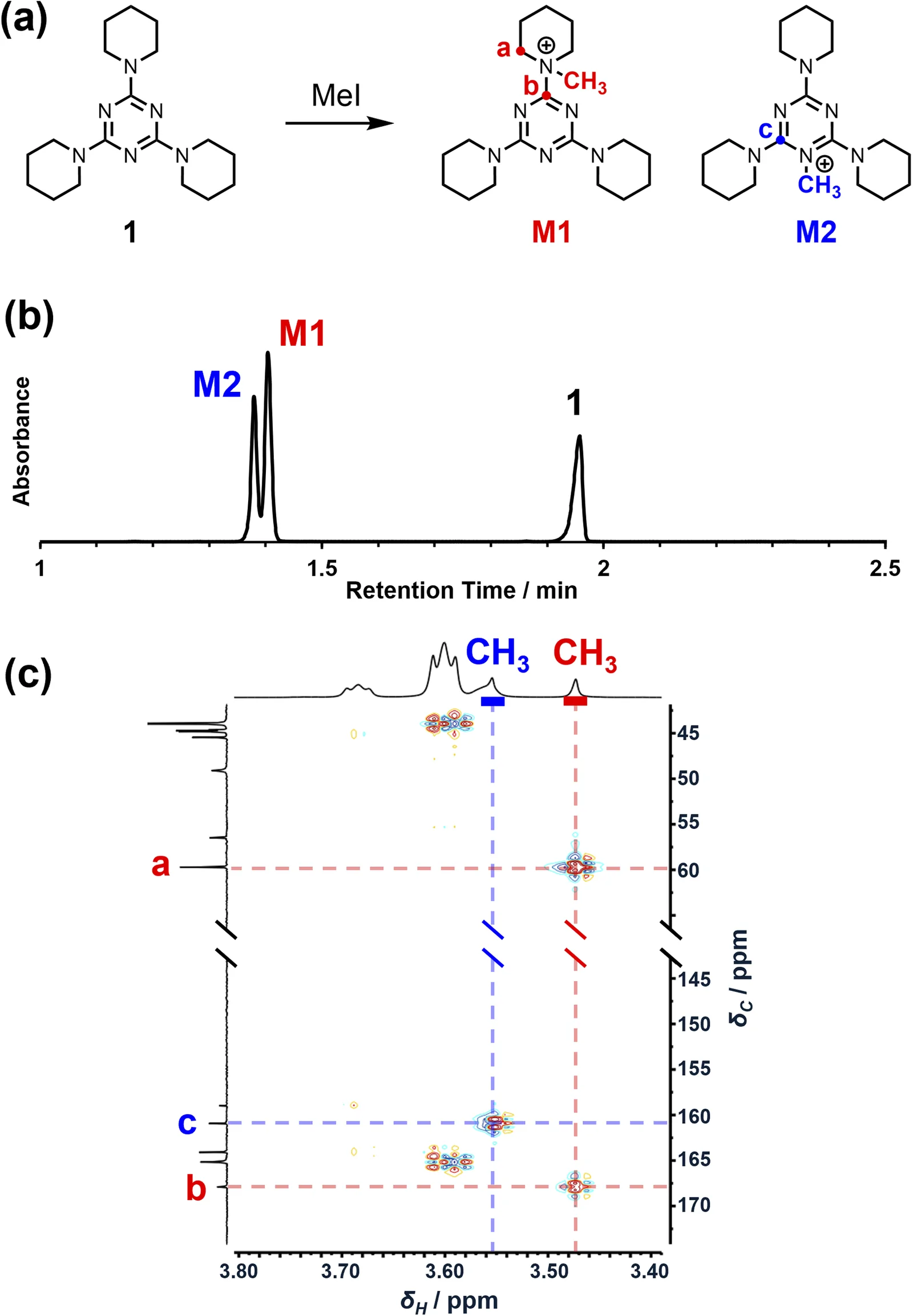
(a) Products of reaction of 1 with methyl iodide/DMF (80 : 20) for 72 hours; (b) UPLC trace (254 nm) of the crude product mixture; (c) 700 MHz ^1^H–^13^C HMBC NMR spectrum (CDCl_3_, 298 K) of the crude product confirming the identities of regioisomers M1 and M2. UPLC details: C18 column at 40 °C using water + 0.1% formic acid (A) and MeCN + 0.1% formic acid (B); 5–100% B over 2 minutes then 100% B for 1 minute.

When compound 1 was stirred in methyl iodide solution (80% in DMF) for three days, two products were observed by LCMS, each with a mass corresponding to addition of a single methyl group (Fig. 3). NMR analysis of the crude reaction mixture confirmed the presence of two regioisomers: the *N*-triazinylammonium compound, M1, and compound M2 which had undergone methylation on a triazine nitrogen. The progress of the reaction was followed by UPLC over a two week period, and the conversion of starting material did not increase after three days (Fig. S22). Heating the reaction mixture, adding silver oxide, or using methyl triflate as the electrophile did not increase the yield of methylation.

**Fig. 4:**
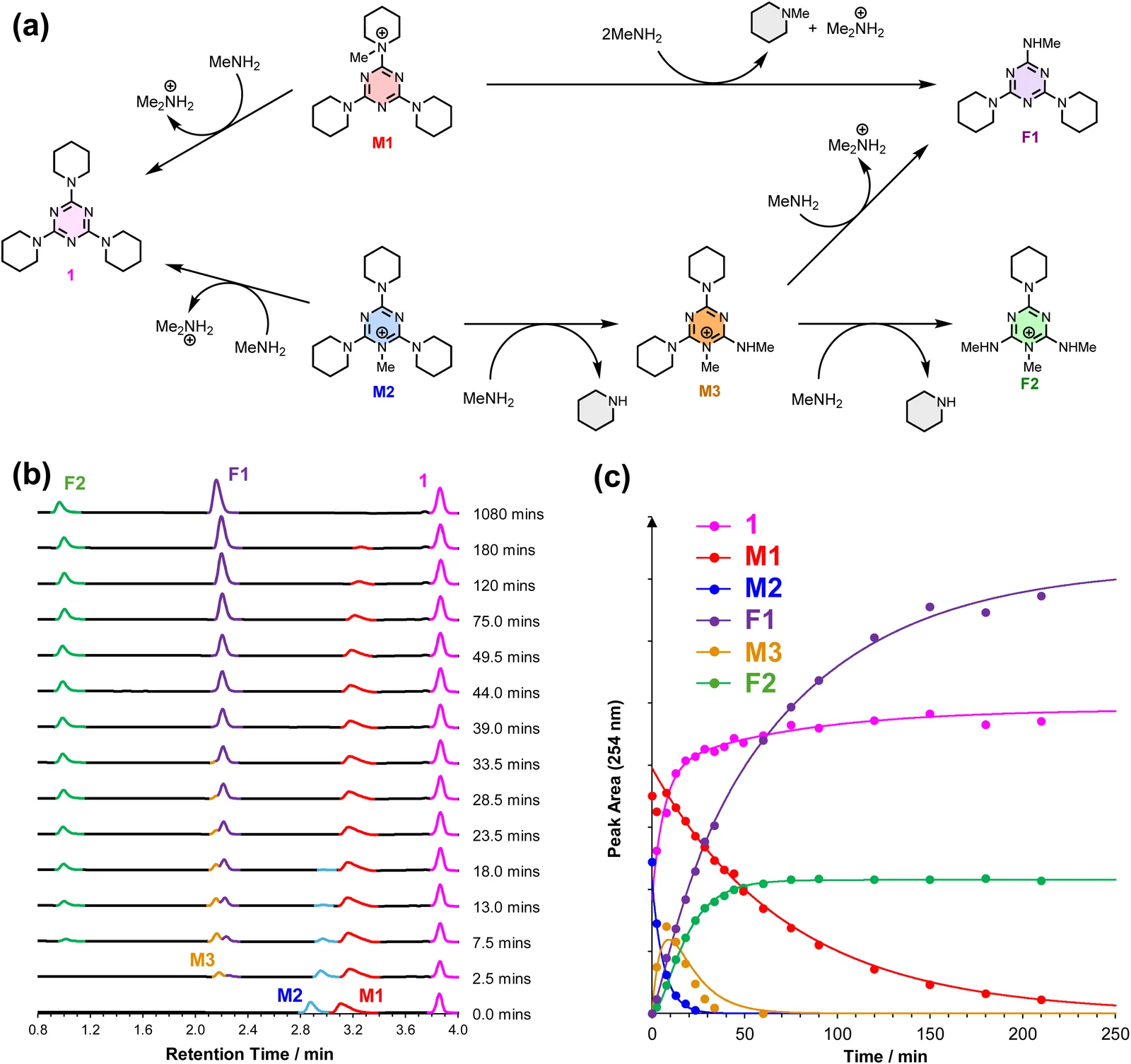
Reaction of an aliquot of the mixture of 1, M1 and M2 obtained from the reaction with methyl iodide with methylamine (33 wt% in EtOH) at room temperature (RT). (a) Reaction network. (b) HPLC traces (254 nm) of the reaction mixture at different time points (peaks were assigned using the corresponding mass spectra). (c) Time evolution of the product distribution. The data points are the integrals of the HPLC peaks, and the lines are simulated values obtained by fitting the experimental data to a kinetic model for the reaction network (see SI for details). HPLC details: CORTECS C8 column (2.7 µm, 4.5 × 50 mm) at 22 °C using water (A) and MeCN (B) flow: 1.7 mL min^−1^; gradient 0.00 min 22% B, 0.25 min 26% B, 1.40 min 26% B, 1.45 min 36% B, 2.70 min 36% B, 2.75 min 80% B, 3.35 min 80% B, 3.40 min 22% B.

When the mixture of 1, M1 and M2 obtained from methylation reaction was treated with benzylamine (neat), the methylated compounds M1 and M2 were consumed. Although two new compounds corresponding to fragments of compound 1 were detected by LCMS, a significant proportion of M1 and M2 were also demethylated to regenerate compound 1. A variety of nucleophiles and conditions were screened in order to identify conditions that minimise this demethylation pathway (Table S2). Secondary amine nucleophiles resulted in almost exclusive demethylation, suggesting that sterics played a key role in determining the relative rates of the fragmentation and demethylation processes. Methylamine (33 wt% in EtOH) gave the greatest yield of the fragmented products. Fig. 4 shows the time course for the reaction of a mixture of 1, M1 and M2 in methylamine solution (33 wt% in EtOH), and the structures of the two triazine products, F1 and F2, which were isolated and characterised by NMR spectroscopy and X-ray crystallography (Fig. S3–S8). The data were consistent with the network of nucleophilic aromatic substitution and demethylation reactions shown in Fig. 4a. Fig. 4c shows the fit of the HPLC data to a kinetic model for this reaction network. It was not possible to obtain a good fit if any of the processes shown in Fig. 4a were neglected from the model (Fig. S26). This analysis suggests that fragmentation process results in two piperidine fragments – piperidine and *N-*methylpiperidine – as well as the two triazine fragments, F1 and F2.

**Fig. 5:**
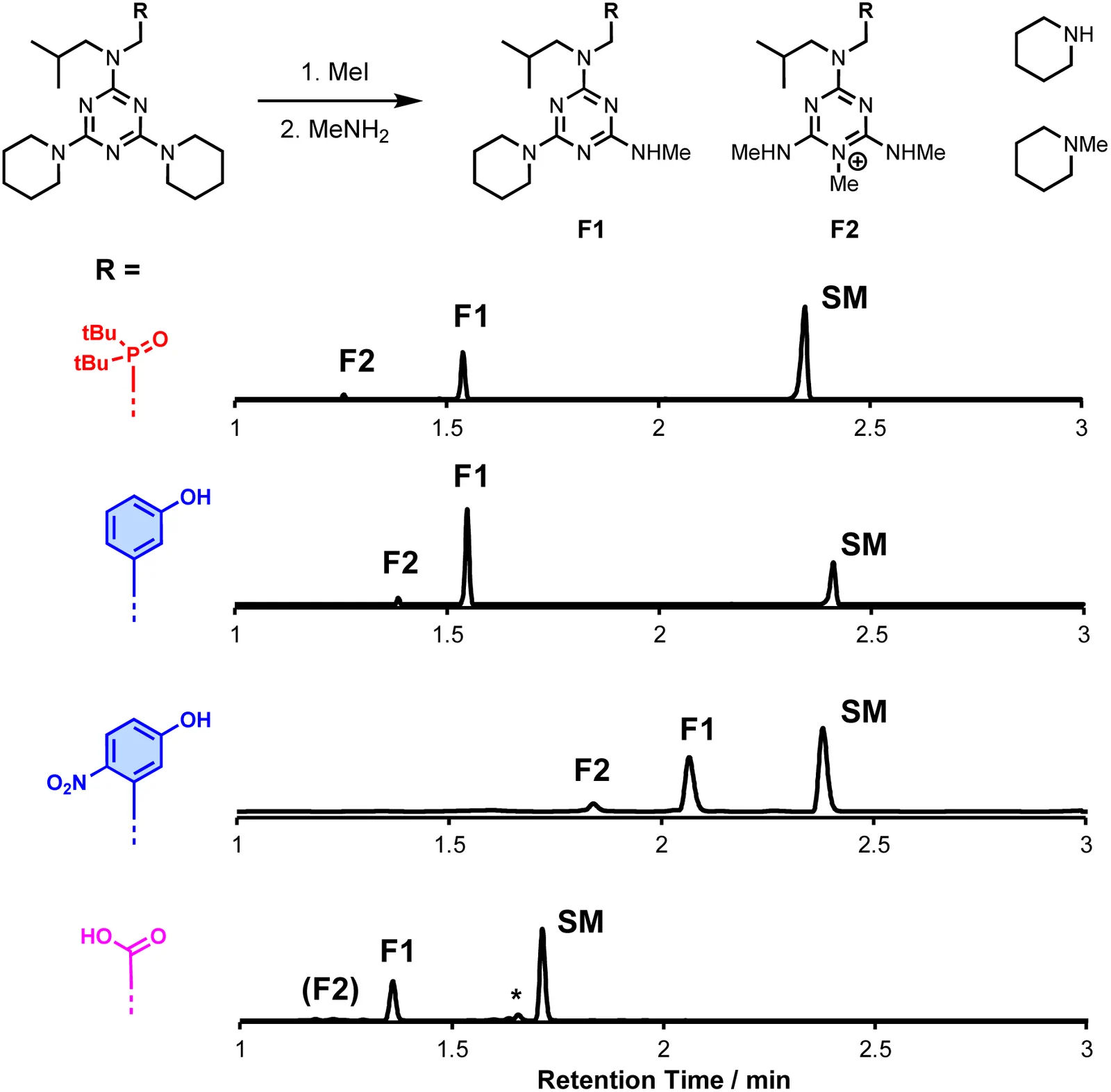
Fragmentation of REMO 1-mers by reaction with methyl iodide/DMF (80 : 20) for 72 hours followed by reaction with methylamine (33 wt% in EtOH) for 15 hours (RT). Two fragment types, F1 and F2, were detected in each case. SM is the unreacted starting material, and the peak labelled with an asterisk corresponds to the methyl amide of the carboxylic acid. UPLC conditions: C4 column at 40 °C using water + 0.1% formic acid (A) and MeCN + 0.1% formic acid (B); 5–100% B over 2 minutes then 100% B for 1 minute. For the nitrophenol 1-mer, the B solvent was THF + 0.1% formic acid.

To investigate whether these reaction conditions show selectivity for fragmentation of the backbone over cleavage of the side chains which carry the sequence information, four different REMO 1-mers with different side chains were studied (Fig. 5). When these compounds were subjected to the two-step fragmentation process, two types of triazine fragment, F1 and F2, were observed in each case (Fig. 5). Importantly, no cleavage of the exocyclic triazine–nitrogen bond connecting the side chain was ever observed, presumably due to the greater steric bulk around the side chain nitrogen atoms, which inhibits one or both of the methylation and aminolysis steps.

**Fig. 6:**
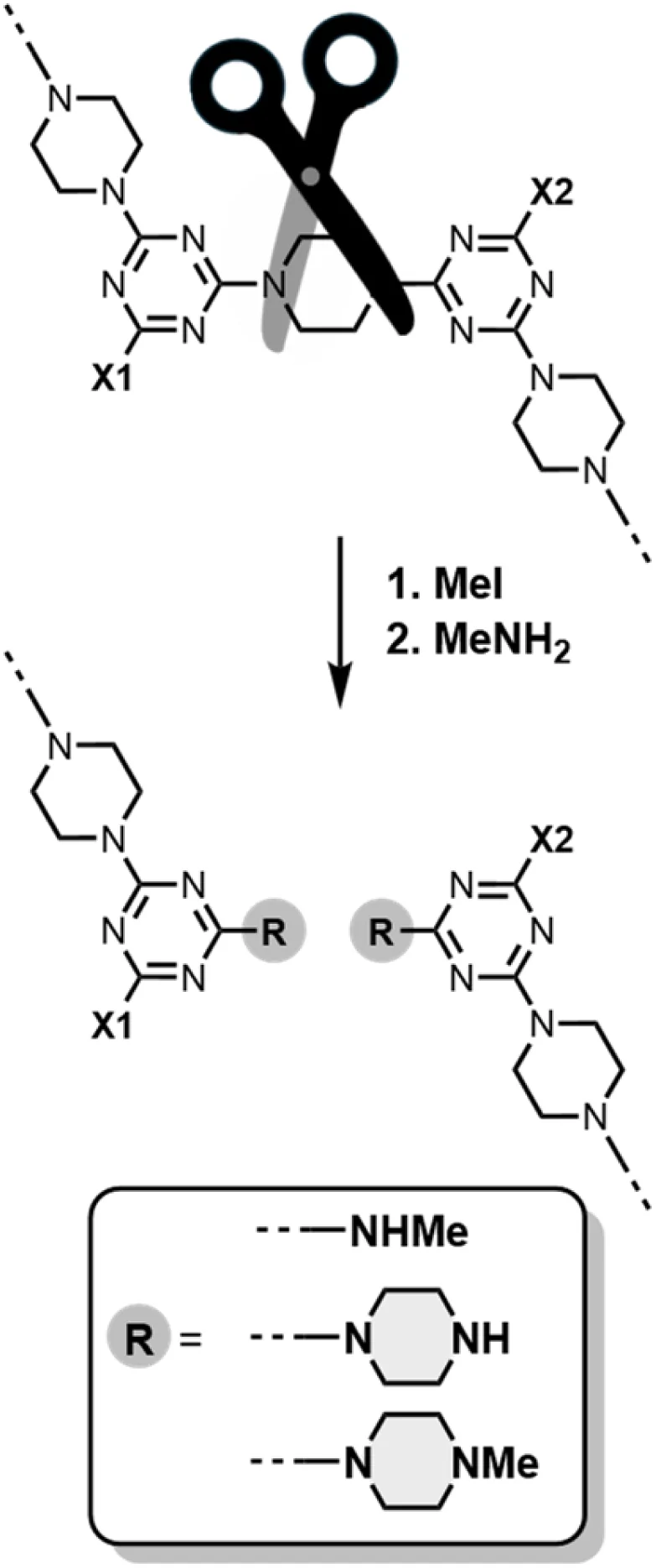
Chemical fragmentation of a REMO gives three different types of fragment for each of the triazines on either side of the cut: one that terminates in methylamine, one that terminates in piperazine, one that terminates in *N*-methylpiperazine.

The experiments described above show that the REMO backbone can be chemically fragmented in a two-step procedure: methylation with methyl iodide followed by aminolysis with methylamine. Cleavage of the recognition units does not occur. Fig. 6 illustrates how this procedure would translate to fragmentation of a longer REMO, resulting in six different types of oligomeric fragment. Cleavage of the backbone between two triazines bearing X1 and X2 side chains generates two fragment strands, each of which could be terminated in one of three different R groups: methylamine, piperazine or *N*-methylpiperazine.

### Sequence determination

Fig. 5 shows that the chemical fragmentation procedure results in only partial, not complete, fragmentation of REMO. Therefore, when a longer REMO is treated with the fragmentation conditions, the backbone is partially degraded to generate a mixture of different length fragments. REMO with different composition have unique molecular weights, so the composition of bases in each fragment can be determined by LCMS, which then enables reconstruction of the sequence of the original REMO.

**Fig. 7:**
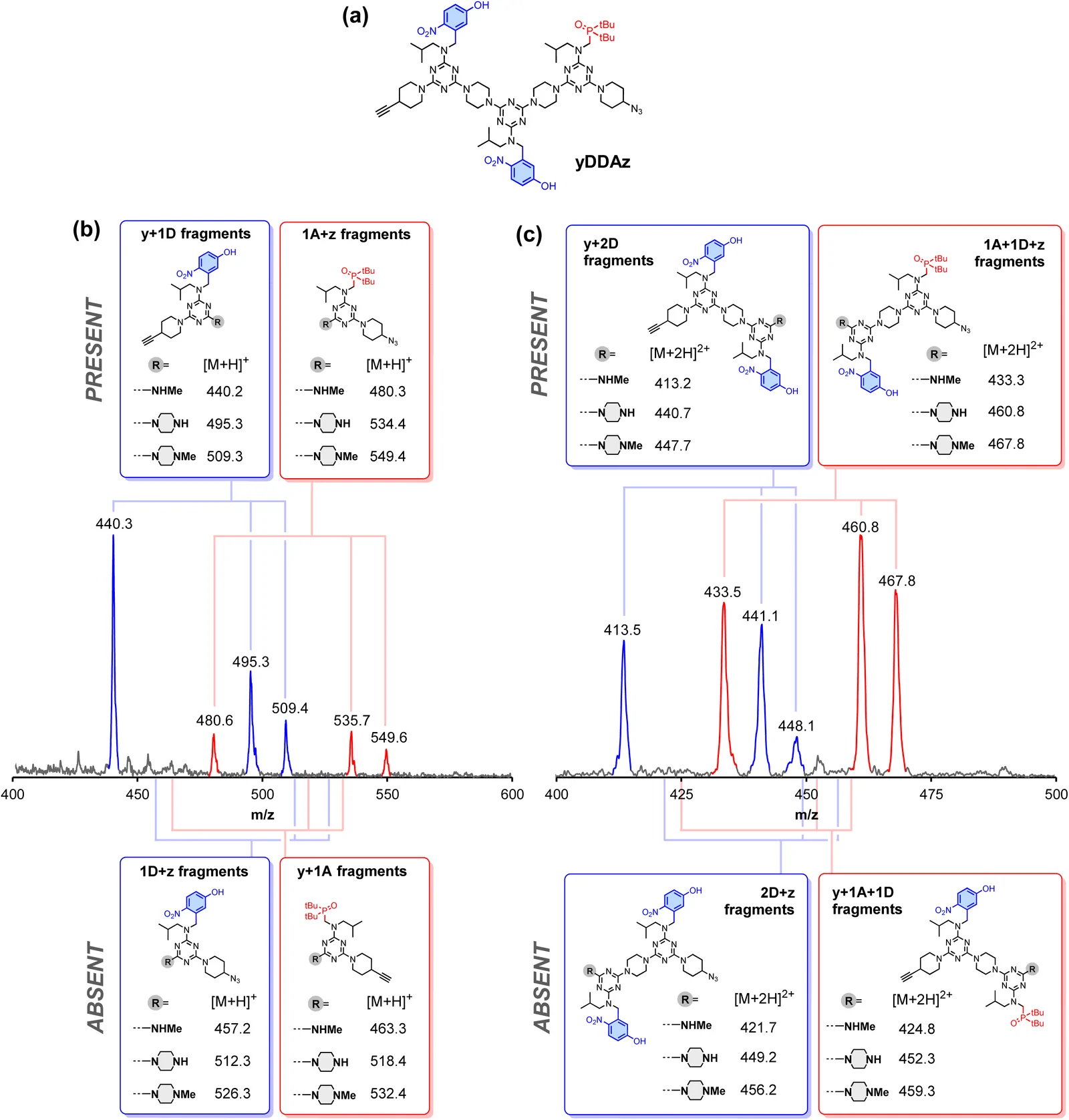
Sequencing of the REMO 3-mer yDDAz. (a) Structure of yDDAz. (b) Structures of all possible 1-mer fragments that could be obtained from all possible sequence isomers of yDDAz, and ESI(+) mass spectrum of the 1-mer region of the UPLC trace (0.4–1.9 min retention times). (c) Structures of all possible 2-mer fragments that could be obtained from all possible sequence isomers of yDDAz, and ESI(+) mass spectrum of the 2-mer region of the UPLC trace (1.9–2.8 min retention times).

As a test of the fragmentation method, a mixed sequence REMO 3-mer yDDAz (y = terminal alkyne, D = 4-nitrophenol, A = phosphine oxide, z = terminal azide, see Fig. 7a) was synthesised and subjected to the two-step fragmentation process, and the products were analysed by LCMS (see SI for synthetic details). Fragments of the same length have similar retention times, so mass spectra were produced by combining data over the relevant retention time windows. Fig. 7b shows the mass spectrum of the 1-mer fragments, and Fig. 7c shows the mass spectrum of the 2-mer fragments. In the 1-mer mass spectrum, three fragments with composition y+1D with different R groups were observed, as expected from the fragmentation illustrated in Fig. 6. Similarly, three 1A+z fragments with different R groups were observed. In contrast, none of the corresponding y+1A or 1D+z fragments were observed. The 2-mer mass spectrum in Fig. 7c shows the presence of three y+2D fragments and three 1A+1D+z fragments, but none of the corresponding 2D+z or y+1A+1D fragments. These data unequivocally confirm the sequence of the yDDAz 3-mer, and show that the same fragmentation behaviour is observed for REMO 1-mers and longer oligomers.

**Fig. 8:**
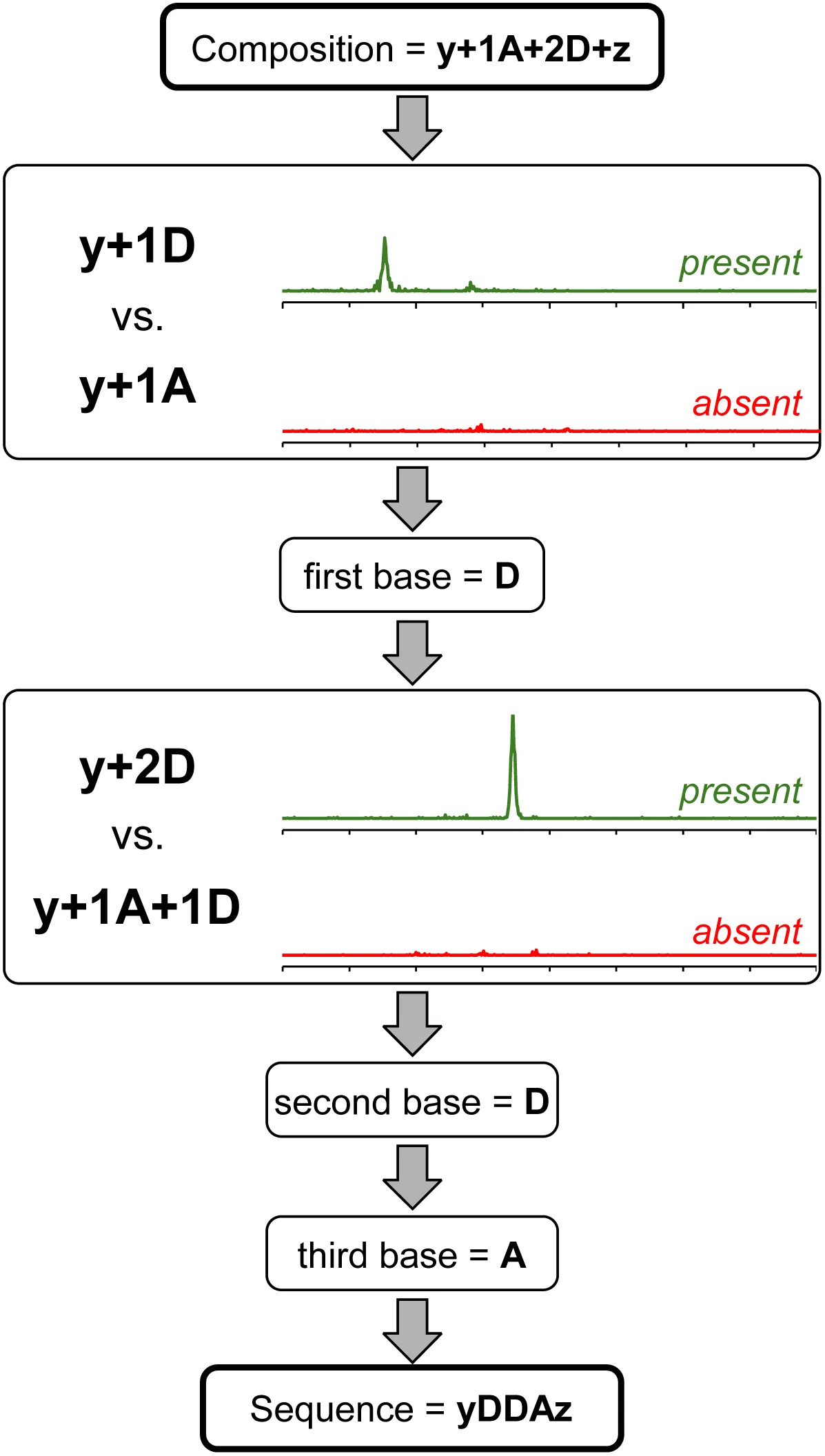
*De novo* sequence determination of a REMO 3-mer. The presence or absence of fragments is determined using extracted-ion chromatograms (EICs). Only fragments terminating in methylamine are considered here, but any or all of the fragment types can be used.

Whilst the data in Fig. 7 confirms the sequence of the REMO 3-mer, Fig. 8 demonstrates how the sequence could be algorithmically determined without prior knowledge (*de novo*), using the same LCMS data. Extracted-ion chromatograms (EICs) are used to determine whether fragments of a given composition are present in the fragment mixture: observation of a clear peak in the EIC is used to positively identify the presence of the fragment. In the first step, the EIC for fragments with composition y+1D and y+1A are compared. The y+1D fragment is clearly identified as present, and the y+1A fragment is absent. This proves that the first base is D, and so the REMO sequence begins yD. To determine the identity of the second base, the EIC for fragments with composition y+2D and y+1A+1D are compared. In this step, only the y+2D is present, so the REMO sequence continues yDD. At this point, there is only one unassigned base remaining, so the third base must be A, and the REMO sequence is yDDAz. In this example, only fragments terminating in methylamine are shown for simplicity, however in principle any, or a combination, of the different fragment types could be used for sequence determination. In fact, using multiple different fragment types will improve confidence in fragment detection.

**Fig. 9:**
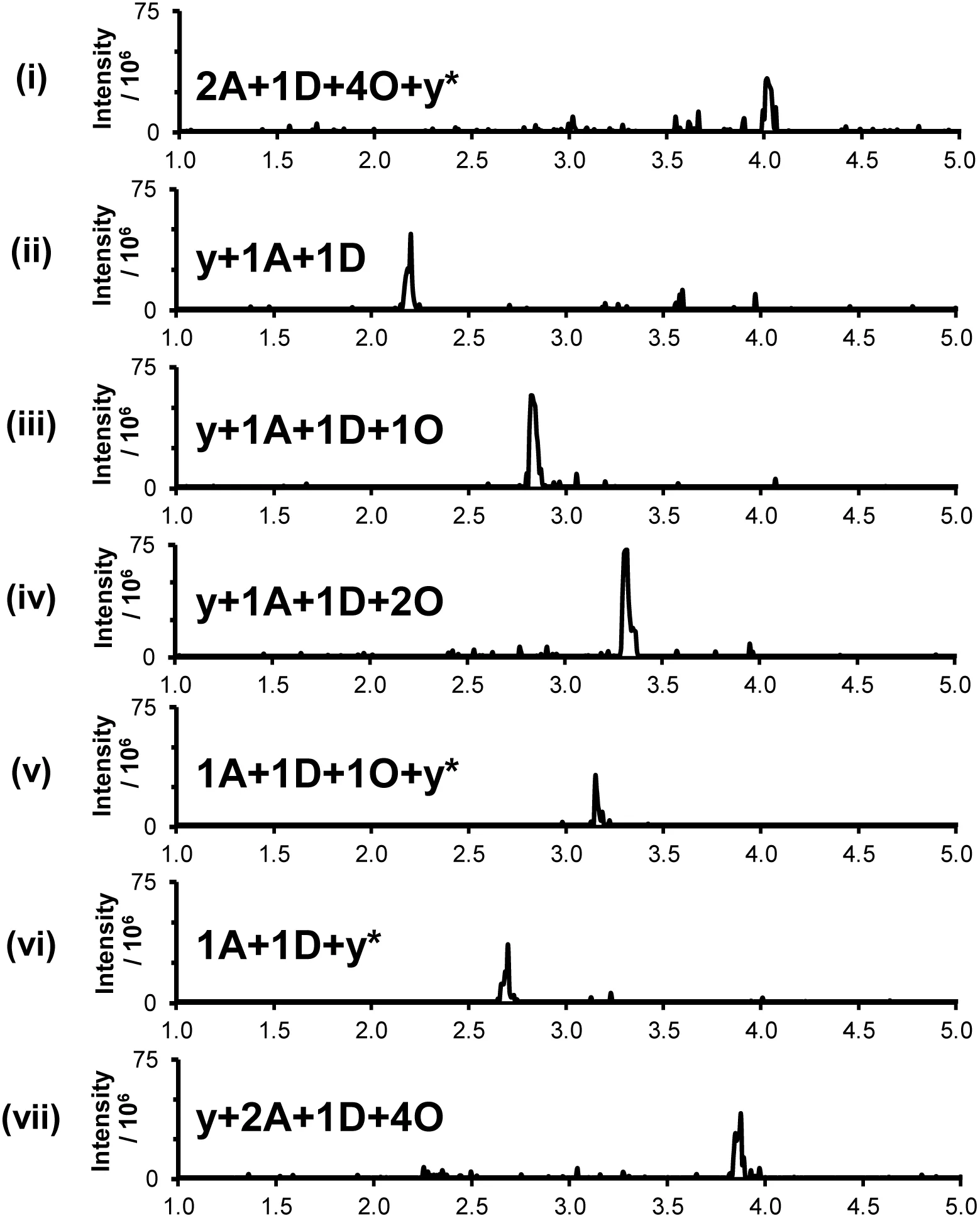
(i)–(vii) Extracted ion chromatograms (*m*/*z* ± 0.5) for some piperazine terminated fragments present in the mixture resulting from fragmentation of a REMO 8-mer with composition y+2A+2D+4O+y*.

**Fig. 10:**
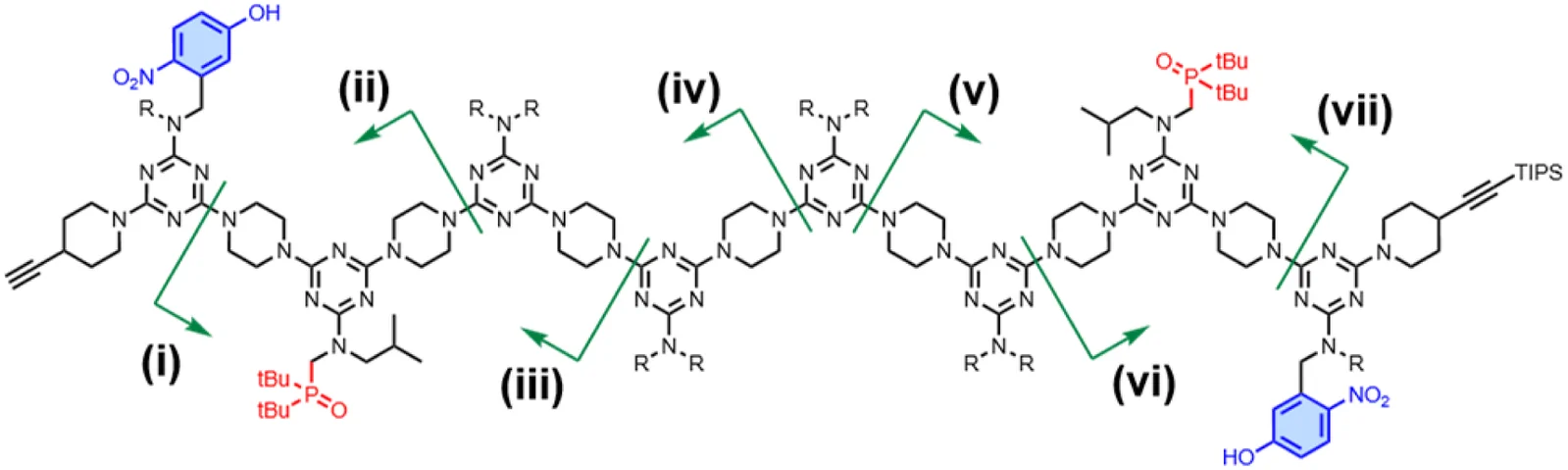
Chemical structure of the REMO 8-mer with sequence yDAOOOOADy* which was sequenced as detailed in Table 1. The green arrows indicate the sites of fragmentation leading to fragments (i)–(vii) in Fig. 9. *R* = 2-ethylhexyl.

To test the performance of the sequencing protocol on longer oligomers, an 8-mer with composition y+2A+2D+4O+y* (O = bis-2-ethylhexyl, y* = TIPS protected terminal alkyne) was subjected to the fragmentation procedure, and the fragment mixture was analysed by LCMS. EICs were used to probe the fragment mixture, and Fig. 9 shows EICs for seven fragments terminating in piperazine that were found to be present, labelled with their composition. Table 1 walks through how the sequence of the 8-mer is decoded using this information. Firstly, observation of a 7-mer fragment with composition 2A+1D+4O+y* (Fig. 9i) implies that the other end of the fragmented chain had the composition y+1D, and this observation is used to assign the identity of the first base as D. Next, observation of the fragment y+1A+1D (Fig. 9(ii)) shows that the second base is A. The other fragment information is sequentially used to decode the 8-mer REMO sequence one base at a time (Table 1). Fig. 10 shows the chemical structure of the REMO yDAOOOOADy*, and the green arrows show the sites of fragmentation that give rise to the fragments observed by EIC in Fig. 9. We have recently shown that this REMO folds into a stable helix in dichloromethane solution, whilst the sequence isomer yDAOOOODAy* folds into a hairpin,^16^ highlighting the importance of REMO primary structure in determining oligomer properties.

**Table 1:** Sequence determination of a REMO 8-mer with composition y+2A+2D+4O+y* using fragments observed in extracted ion chromatograms

Fragment composition	EIC in Fig. 9	Updated REMO sequence
		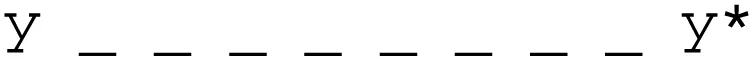
2A+1D+4O + y*	(i)	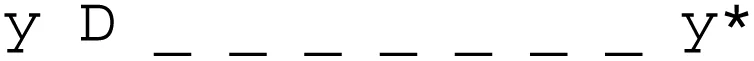
y+1A+1D	(ii)	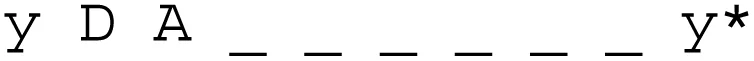
y+1A+1D+1O	(iii)	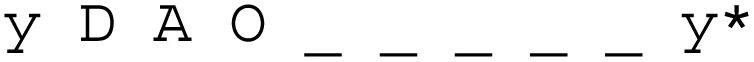
y+1A+1D+2O	(iv)	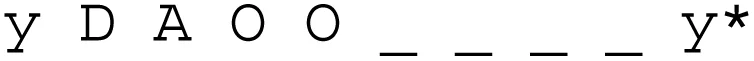
y+2A+1D+4O	(vii)	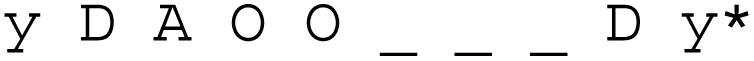
1A+1D + y*	(vi)	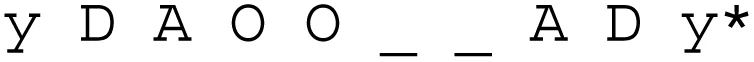
1A+1D+1O + y*	(v)	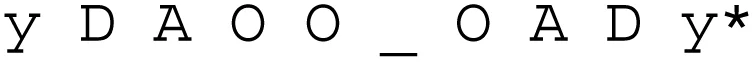
		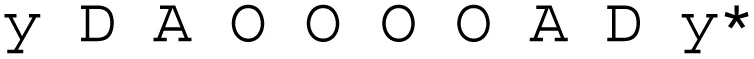

These experiments show that the two-step fragmentation protocol is a reliable method for *de novo* sequence determination of REMO. There are however limitations to the sequencing method such the inability to sequence oligomers within complex mixtures, and the increasing complexity of the fragment mixture with oligomer length. The development of this sequencing method will enable further investigations of sequence-structure–function relationships.

### Analysis of templating fidelity

The development of chemical systems capable of replication with sequence-information transfer was identified by Orgel as the holy grail of organic chemistry.^64^ The development of sequencing methods for synthetic polymers is an essential part of assessing the fidelity of information transfer in such a replication process.^58,65^ We have reported two different strategies for the replication of REMO, templated polymerisation and templated base-filling,^18,19^ but these studies were limited to homo-sequence templates, in part because there was no method for determining the sequence of the products. The sequencing method described here allows us to investigate the fidelity of templating processes using mixed-sequence REMO templates.

**Fig. 11:**
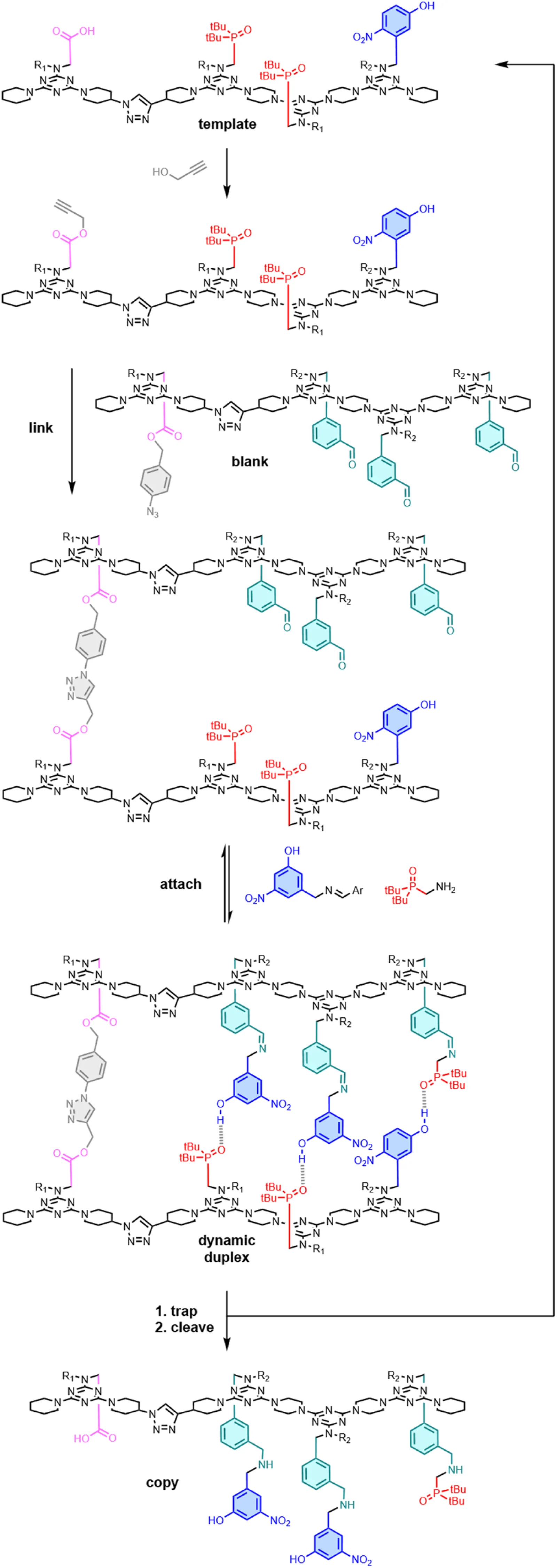
Proposed templating of a mixed-sequence REMO by base-filling. The carboxylic acid recognition unit on the template was first esterified using EDC and propargyl alcohol, which allowed the trialdehyde blank strand to be covalently linked *via* a CuAAC reaction using Cu(i)TBTA. Dynamic imine chemistry was then used to equilibrate complementary 3-nitrophenol and phosphine oxide amines onto the copy strand, giving the dynamic duplex. Reduction of the imines with HSiCl_3_/DMF irreversibly trapped the dynamic duplex, and cleavage of the esters with LiOH regenerated the template and released the base-filled copy (the sequence-complementary copy of the template is illustrated). *R*_1_ = *i*-butyl; *R*_2_ = 2-ethylhexyl.

Fig. 11 shows the base-filling strategy.^19^ The template strand has a carboxylic acid, which is used to covalently link it to a trialdehyde blank strand, and three H-bond recognition units, a 4-nitrophenol and two phosphine oxides, which carry the sequence information (see SI for synthetic details). In the templating process, the carboxylic acid on the template was first esterified with a large excess of propargyl alcohol to prevent any intramolecular reaction with the 4-nitrophenol recognition unit. The blank strand was then attached using a copper-catalysed azide-alkyne cycloaddition (CuAAC) reaction. The next step in the process is dynamic attachment of amines equipped with recognition units to the blank strand using imine chemistry. However, the 3-nitrophenol amine had limited solubility in dichloromethane, so it was first converted to an imine using 3,5-di-*tert*-butylbenzaldehyde. A 12-fold excess of the 3-nitrophenol imine and the phosphine oxide amine was added to a 1 mM dichloromethane solution of the REMO template equipped with the blank strand.

The solvent was removed, the sample was redissolved in toluene to give a template concentration of 0.1 mM, and the mixture was left to equilibrate overnight. The expected product is the dynamic duplex shown in Fig. 11, where the sequence of recognition units incorporated into the blank strand is complementary to the sequence of recognition units on the template. Reduction of this mixture with trichlorosilane (0.5 M) and DMF (0.5 M) trapped the imines as the corresponding secondary amines, and the ester linkers were then hydrolysed using lithium hydroxide to regenerate the original template and release the base-filled copy strand.

**Fig. 12:**
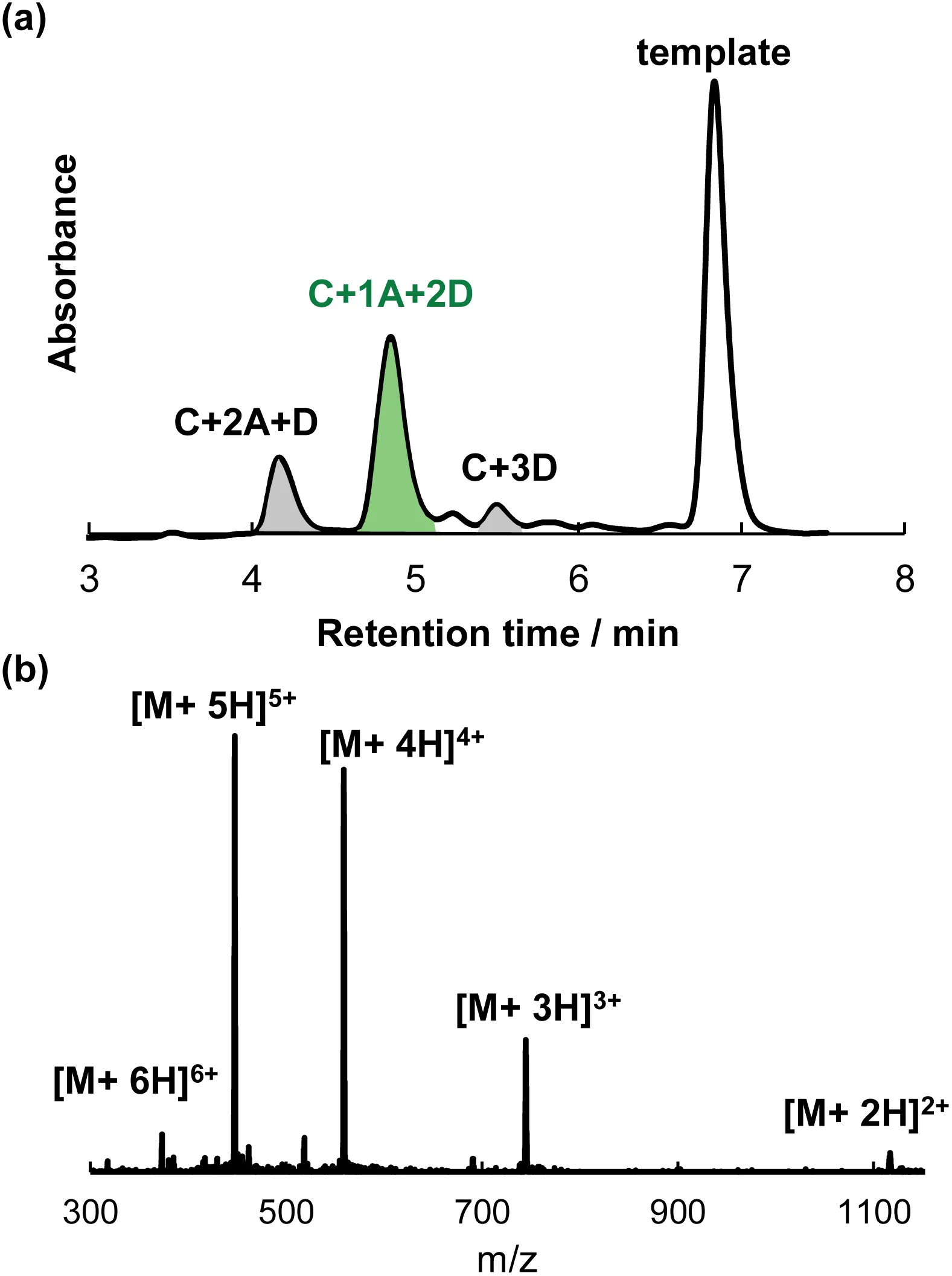
(a) HPLC trace (254 nm) of the products of the replication cycle shown in Fig. 11. Peaks due to the copy strands are labelled according to the composition of recognition units identified in the corresponding mass spectra. HPLC conditions: C8 column at 40 °C using water/acetonitrile (95 : 5) + 0.1% trifluoroacetic acid (A) and THF + 0.1% trifluoroacetic acid (B); gradient of 50–65% B over 9 minutes. (b) Mass spectrum (ESI+) of the isolated C+1A+2D product.

Fig. 12a shows the HPLC trace of the resulting mixture, which contains the template and three different types of copy strand, which were identified using the corresponding mass spectra. The copy strands are labelled according to the composition of recognition units (C = carboxylic acid, A = phosphine oxide and D = 3-nitrophenol). Copy strands of identical recognition unit composition but different sequences could not be resolved by HPLC. The major product was C+1A+2D, which would be consistent with the sequence-complementary copy of the template (CDDA) shown in Fig. 11. The two minor products in Fig. 12a are C+2A+1D and C+3D, which are copies that contain one mismatch. The C+3A copy, which would contain two mismatches, was not observed. Assuming the products have similar extinction coefficients, the yield of C+1A+2D can be estimated to be 68%, which represents a significant template effect (a statistical mixture would contain 19% of this composition, see Table S4). The C+1A+2D copy was isolated by semi-preparative HPLC (Fig. 12b), and this sample was used for sequencing.

**Fig. 13:**
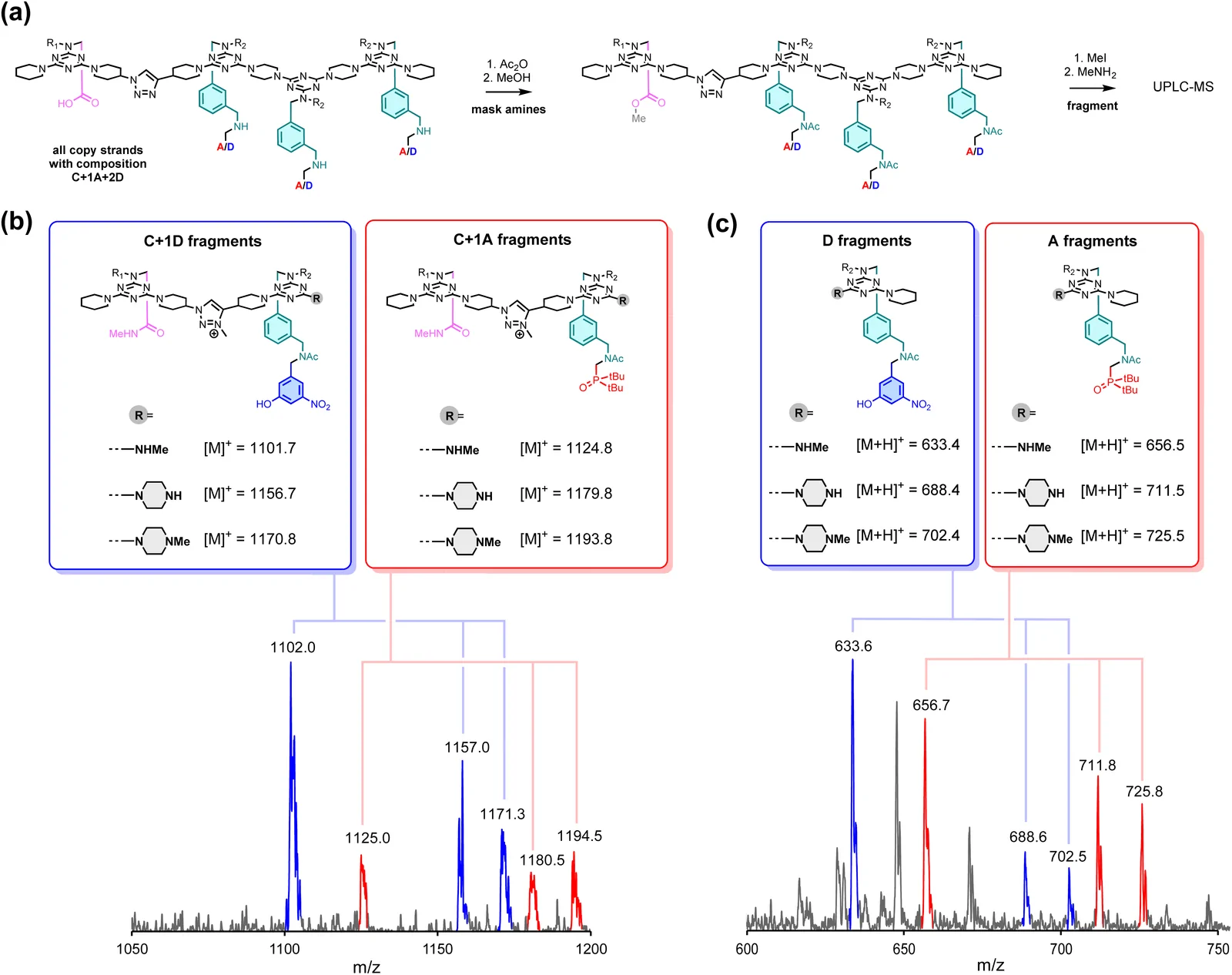
Fragmentation of the C+1A+2D product obtained from the templating cycle shown in Fig. 11. (a) Preparation of the C+1A+2D for fragmentation. (b) Chemical structures of 2-mer fragments that could be obtained from different sequences with composition C+1A+2D, and ESI(+) mass spectrum of the associated region of the UPLC trace (1.70–1.85 min retention times). (c) Chemical structures of 1-mer fragments that could be obtained from different sequences with composition C+1A+2D, and ESI(+) mass spectrum of the associated region of the UPLC trace (1.80–1.90 min retention times). *R*_1_ = *i*-butyl; *R*_2_ = 2-ethylhexyl. The peaks in grey come from different fragments with similar retention times.

The C+1A+2D sample was treated with acetic anhydride followed by methanol in order to mask the secondary amines, which would otherwise react with methyl iodide. The carboxylic acid recognition unit was converted quantitatively to the corresponding methyl ester during this process. This sample was then subjected to the fragmentation procedure described above, and the fragment mixture was analysed by LCMS (Fig. 13). Tests on a model compound showed that the triazole is quantitatively methylated under the fragmentation conditions (Fig. S29–S32) and the methyl ester is quantitatively converted to the *N*-methyl amide. Fig. 13a shows the mass spectrum of the 2-mer fragments obtained from the carboxylic acid end of the copy strand. Although the intensity of the MS peaks corresponding to incorporation of 3-nitrophenol are larger, it is clear that a significant number of copy strands contain a phosphine oxide recognition unit at this position. Similarly, the mass spectrum of the 1-mer fragments obtained from the other end of the copy strand show incorporation of both 3-nitrophenol and phosphine oxide at this position (Fig. 13b). These results show that the C+1A+2D sample contains more than one sequence, so although there is good selectivity for the recognition unit composition in the templating process, the sequence has been scrambled. Presumably the dynamic duplex is sufficiently flexible for some crossover to take place, such that recognition units on the template can base-pair with complementary side chains at multiple sites on the copy strand.

## Conclusions

A method for the sequence determination of recognition-encoded melamine oligomers (REMO) has been developed using a backbone fragmentation strategy. Selective methylation of nitrogen atoms on backbone rather than the side chains can be achieved using methyl iodide. This reaction generates electrophilic sites on the backbone that undergo aminolysis in methylamine solution. The resulting chain cleavage generates three different fragments that can be detected by LCMS. This procedure was verified as a viable sequencing method for REMO of different length equipped with a variety of different side chains. The sequencing method was used to investigate the fidelity of a template-directed synthesis process using base-filling.^19^ Dynamic imine chemistry was used to incorporate amines equipped with 3-nitrophenol (D) or phosphine oxide (A) recognition units onto a trialdehyde blank strand, which was connected to a template strand *via* ester linkages between carboxylic acids (C) on the two strands. In the presence of a mixed-sequence template equipped with 4-nitrophenol and phosphine oxide recognition units, preferential incorporation of complementary recognition units into the blank strand was observed. Specifically, when a template with the sequence CAAD was used, a 68% yield of a copy strand with the sequence-complementary composition C+1A+2D was observed after trapping the dynamic duplex by reduction and cleaving the esters connecting the two strands. However, sequencing of the C+1A+2D copy revealed that significant amounts of scrambling had occurred in the replication process, and the copy strand contained multiple sequence isomers. Just as sequencing has underpinned major advances in biochemistry and molecular biology, the development of the sequencing method described here establishes an important tool that will enable further investigations of sequence-structure–function relationships using synthetic polymers based on the REMO architecture.

## Author contributions

The manuscript was written through contributions of all authors.

## Conflicts of interest

There are no conflicts to declare.

## Supplementary Material

SC-017-D6SC05680F-s001

SC-017-D6SC05680F-s002

## Data Availability

CCDC 2532820 and 2532821 contain the supplementary crystallographic data for this paper.^66*a*,*b*^ All supporting data is provided in the supplementary information (SI). Supplementary information: materials and methods, detailed synthetic procedures, characterisation including NMR spectra and HRMS of all compounds, kinetic analysis, and supplementary sequencing experiments. See DOI: https://doi.org/10.1039/d6sc05680f.
