# Supplementary material for: Finding REMO: a sequencing method for recognition-encoded melamine oligomers
Source: Chem Sci. 2026 Jul 29. Online ahead of print. doi: 10.1039/d6sc05680f (PMC13418241; doi:10.1039/d6sc05680f)
Supplement: SC-OLF-D6SC05680F-s001 [file SC-OLF-D6SC05680F-s001.pdf]

Supplementary Information (SI) for Chemical Science.
This journal is © The Royal Society of Chemistry 2026

Finding REMO: A Sequencing Method for Recognition-Encoded Melamine Oligomers

Ben Iddon, Pawel H. Grab, Joseph T. Smith, Oliver N. Evans, Anca-Luiza Cotîrlan and Christopher A. Hunter*

Yusuf Hamied Department of Chemistry, University of Cambridge, Lensfield Road, Cambridge CB2 1EW, UK. E-mail: herchelsmith.orgchem@ch.cam.ac.uk

Supporting Information

Table of Contents

1. General Experimental Details 2
2. Sequencing 4
   a. Synthesis and Characterisation 4
   b. Time Course of Methylation 29
   c. Nucleophile Screening 30
   d. HPLC Kinetics Study 32
   e. General Sequencing Procedure 37
   f. Additional Investigations 38
3. Base-Filling 45
   a. Synthesis and Characterisation 45
   b. Imine attachment, trapping and cleavage 78
   c. Relative imine stabilities 79
   d. Analysis of final copy mixture 80
   e. Product sequencing 81
4. References 81

1. General Experimental Details

All the reagents and materials were obtained from commercial sources and used without further purification. Dry solvents were taken from the solvent purification system Pure SolvTM by Innovative Technology, Inc.. Thin layer chromatography was carried out using Silica gel 60F on glass. Flash chromatography was carried out on an automated system (Combiflash Rf+ or Rf Lumen) using pre-packed cartridges of silica (25 μm PuriFlash Column). NMR spectra were recorded on a Bruker 400 MHz AVIII400, 400 MHz Neo Prodigy, 400 MHz QNP cryoprobe, 500 MHz AVIII Smart Probe or 700 MHz TCO cryoprobe spectrometer. The residual solvent was used as the internal standard. In chloroform-*d*, ^{1}H spectra were referenced to δ 7.26 ppm and ^{13}C spectra to 77.16 ppm for the solvent signal. In methanol-d_4, ^{1}H spectra were referenced to δ 3.31 ppm and ^{13}C spectra to 49.00 ppm for the solvent signal. In DMSO-d_6, ^{1}H spectra were referenced to δ 2.50 ppm and ^{13}C spectra to 39.52 ppm for the solvent signal. In acetonitrile-d_3, ^{1}H spectra were referenced to δ 1.94 ppm and ^{13}C spectra to 1.32 ppm for the solvent signal. All chemical shifts are quoted in ppm on the δ scale. Splitting patterns are given as follows: s (singlet), d (doublet), t (triplet), q (quartet), p (pentet), sept (septet), non (nonet) and m (multiplet), broad peaks are denoted br. ^{1}H and ^{13}C NMR spectra were assigned as far as possible using DEPT, COSY, HSQC and HMBC spectra. Psuedo-equivalent environments are labelled with the same number and where there are multiple peaks that are not fully resolved, from pseudo-equivalent environments or rotamers, peaks are grouped in square brackets. Where multiple peaks arise only from rotamers, this is specified. A Waters LCT premier mass spectrometer was used to obtain the ESI+ mass spectra. FT-IR spectra were measured on a PerkinElmer Spectrum One spectrometer equipped with an ATR cell. The LCMS analysis of samples was performed using Waters Acquity H-class UPLC coupled with a single quadrupole Waters SQD2. Unless otherwise noted, an ACQUITY UPLC BEH C4 Column, 300 Å, 1.7 μm, 2.1 mm × 50 mm was used and the conditions of the UPLC method are as follows. Solvent A: Water + 0.1% Formic acid; Solvent B: THF + 0.1% Formic acid; Gradient of 0-4 minutes 30% - 100% B then 2 minutes 100% B with re-equilibration time of 2 minutes. Flow rate: 0.4 ml/min; Column temperature of 40 °C; Injection volume of 2 μL. The signal was monitored at 254 nm. Semi-preparative reverse-phase HPLC was performed on an Agilent HP-1100 Series HPLC system. XBridge BEH C8 OBD Prep column, 130 Å, 5 μm, 10 mm × 100 mm was used as the HPLC column The conditions of the HPLC method are as

follows: Solvent A: Water/acetonitrile (95:5) + 0.1% trifluoroacetic acid, Solvent B: THF + 0.1% trifluoroacetic acid; gradient of 0-9 minutes 50%-65% B with re-equilibration time of 2 minutes. Flow rate: 4 mL/min; column temperature of 40 °C; injection volume of 10 μL. The signal was monitored at 254 nm. Single-crystal X-ray diffraction data for **F1** and **F2** were collected on a Bruker D8-QUEST diffractometer, equipped with an Incoatec IμS Cu microsource (λ = 1.54178 Å) and a PHOTON-III detector. The temperature was maintained at 180(2) K. Data collection was performed using Bruker *APEX5*. Diffraction images were integrated using *SAINT*. Multi-scan absorption corrections were applied using *SADABS*. The structures were solved using *SHELXT* and refined using *SHELXL*. Molecular graphics were generated using Mercury.

2. Sequencing

a. Synthesis and Characterisation

Synthesis of 1

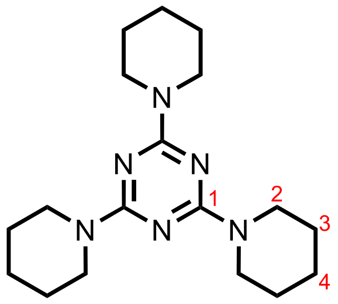

Cyanuric chloride (3.00 g, 16.3 mmol, 1.0 eq) was dissolved in dry THF (20 mL) and piperidine (32 mL, 325 mmol, 20 eq) was added to the stirred solution dropwise at 0°C. Once addition was complete the reaction mixture was heated under reflux for 90 minutes. The crude product was extracted with ethyl acetate (30 mL), washed with HCl (1 M, 30 mL), water (30 mL) and then brine (30 mL). The organic layer was dried with anhydrous $MgSO_4$ and the organic solvents were removed *in vacuo*. The product was purified by flash column chromatography (SiO_2, 0 – 4% EtOAc in petroleum ether). The solvents were removed *in vacuo* and the purified product was dried *in vacuo* giving **1** as a white solid (1.28 g, 3.88 mmol, 24%).

^{1}H NMR (700 MHz, $CDCl_3$): δ_H = 3.70 (m, 12H, H_2), 1.69 – 1.59 (br quint, *J* = 5.6 Hz, 8H, H_4), 1.56 – 1.51 (m, 12H, H_3);

^{13}C NMR (176 MHz, $CDCl_3$): δ_C = 165.6 (C_1), 44.2 (C_2), 26.0 (C_3), 25.2 (C_4);

FT-IR (ATR): ν_{max} /cm^{-1} 3000, 2932, 2849, 1529, 1480, 1459, 1438, 1373, 1348, 1297, 1271, 1259, 1234, 1129, 1099, 1026, 993, 911, 853, 807, 731.

The spectroscopic data was consistent with the literature.[1]

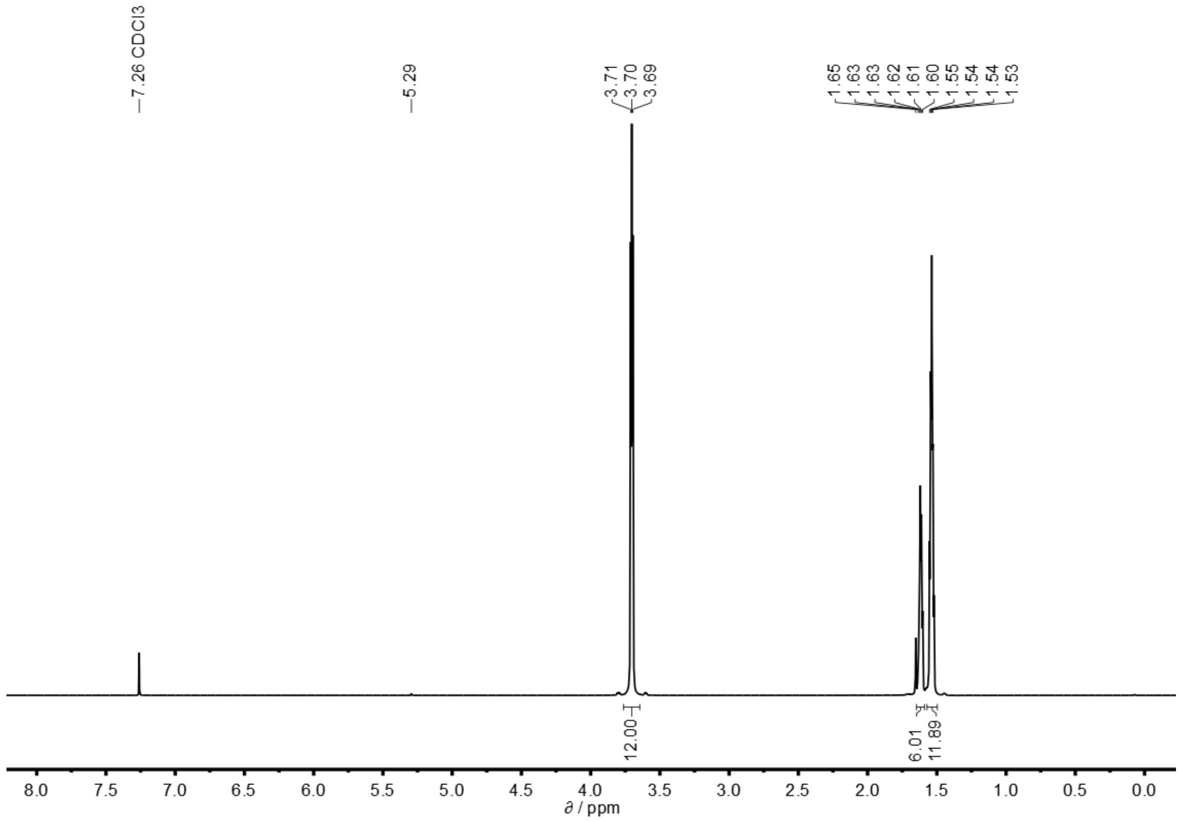

Figure S1. ^{1}H NMR spectrum (700 MHz, $CDCl_3$, 298 K) of **1**.

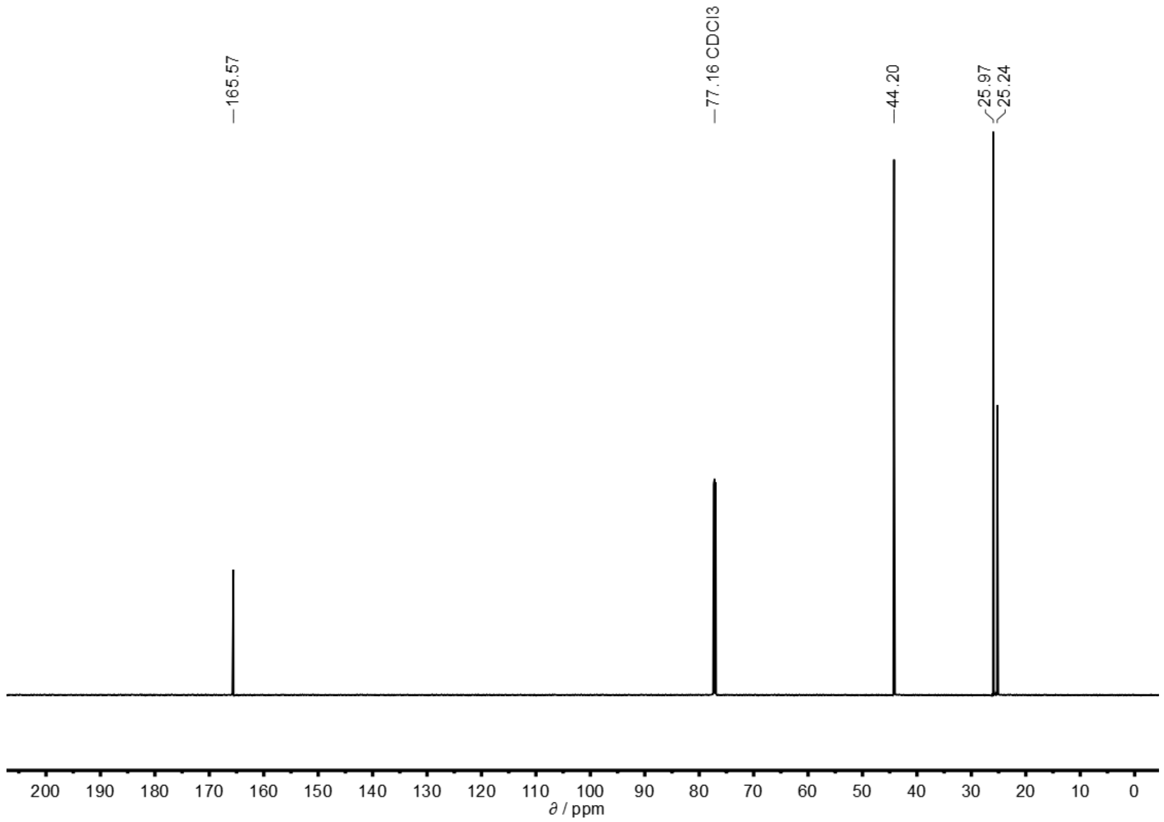

Figure S2. ^{13}C NMR spectrum (176 MHz, $CDCl_3$, 298 K) of **1**.

Synthesis of F1 and F2

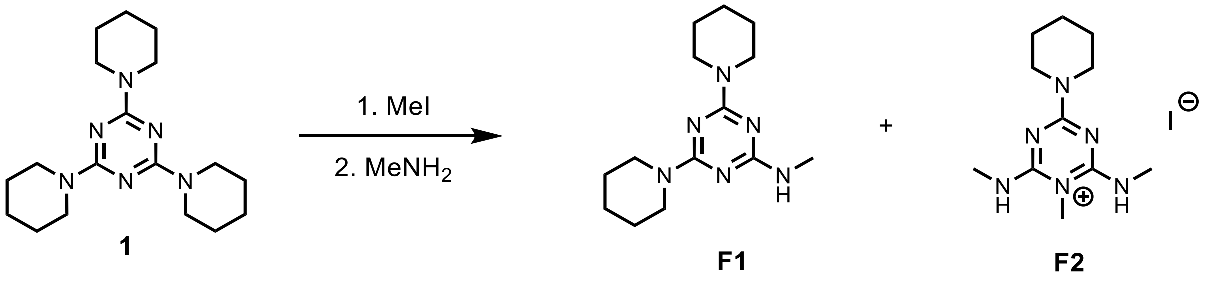

1 (115 mg) was stirred in methyl iodide (2 mL) and DMF (0.5 mL) for 48 hours then the solvents were removed under a stream of nitrogen. The residue was redissolved in methylamine solution (33 wt.% in EtOH, 3 mL) and stirred overnight. The solvents were removed under a stream of nitrogen. The crude was purified by flash column chromatography (C18, 10 - 100% MeCN in H_2O) to obtain **F1** (24 mg) and **F2** (7 mg), both as white solids.

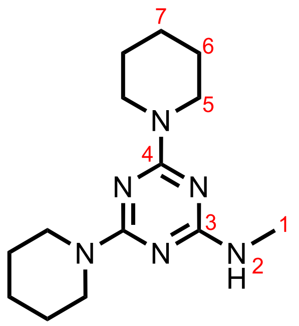

F1

^{1}H NMR (400 MHz, $CDCl_3$): δ_H = 6.50 (q, *J* = 4.8 Hz, 1H, H_2), 3.68 – 3.59 (m, 8H, H_5), 2.72 (d, *J* = 4.7 Hz, 3H, H_1), 1.63 – 1.53 (m, 4H, H_7), 1.46 – 1.42 (m, 8H, H_6);

^{13}C NMR (176 MHz, $CDCl_3$): δ_C = 166.0 (C_3), 164.1 (C_4), 43.4 (C_5), 27.0 (C_1), 25.4 (C_6), 24.4 (C_7);

HRMS (ESI+): calc. for $[C_{14}H_{24}N_6 + H]^+$ is 277.2135, found 277.2140;

FT-IR (ATR): ν_{max} /cm^{-1} 3342, 2929, 2850, 1556, 1521, 1481, 1459, 1438, 1383, 1363, 1291, 1274, 1243, 1186, 1131, 1092, 1024, 991, 852, 808.

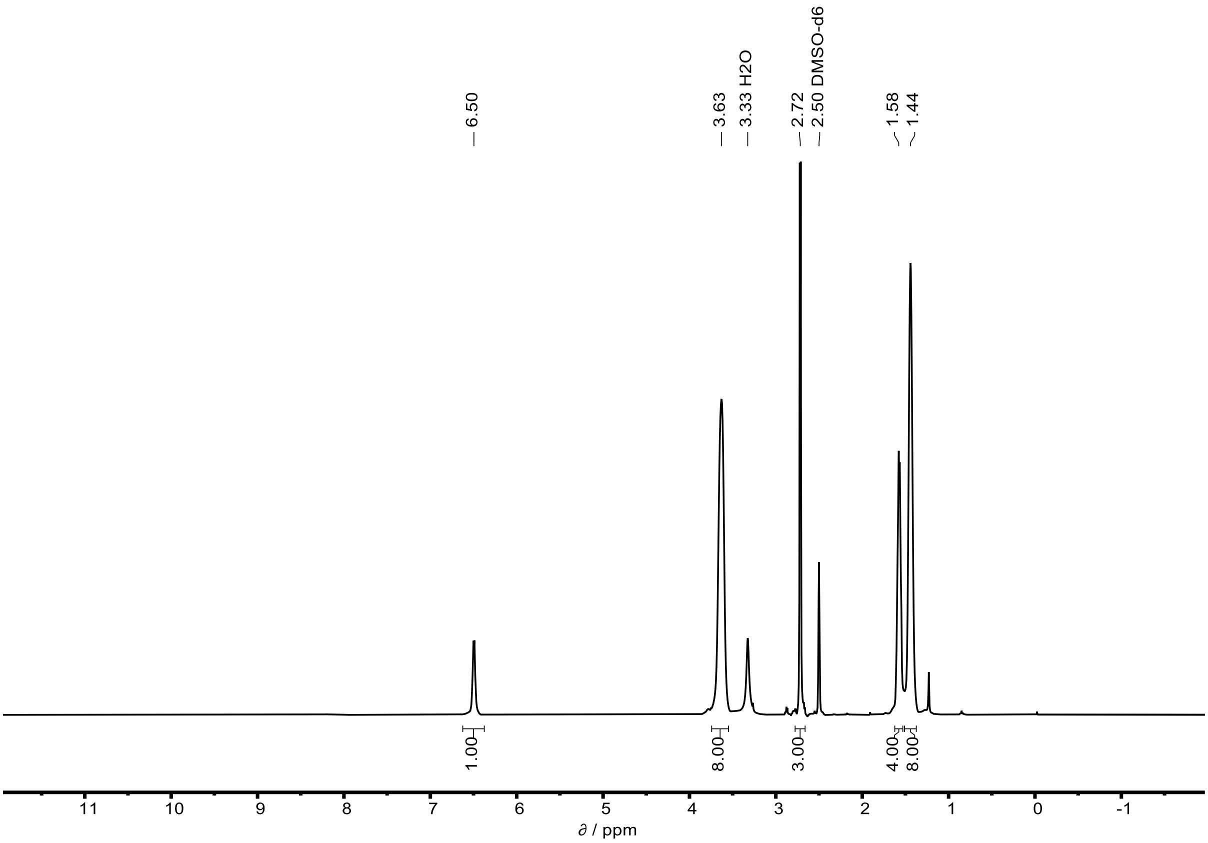

Figure S3. ^{1}H NMR spectrum (400 MHz, DMSO, 298 K) of **F1**.

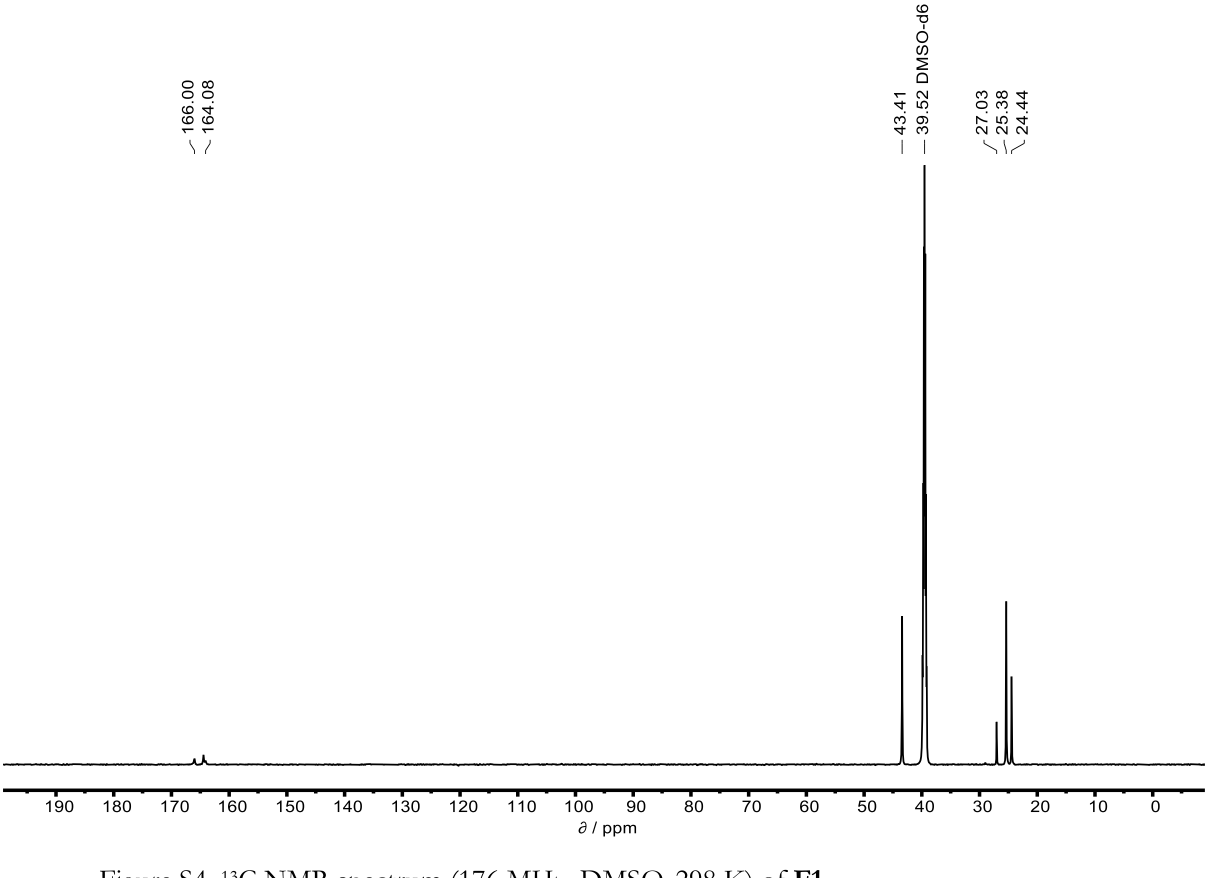

Figure S4. ^{13}C NMR spectrum (176 MHz, DMSO, 298 K) of **F1**.

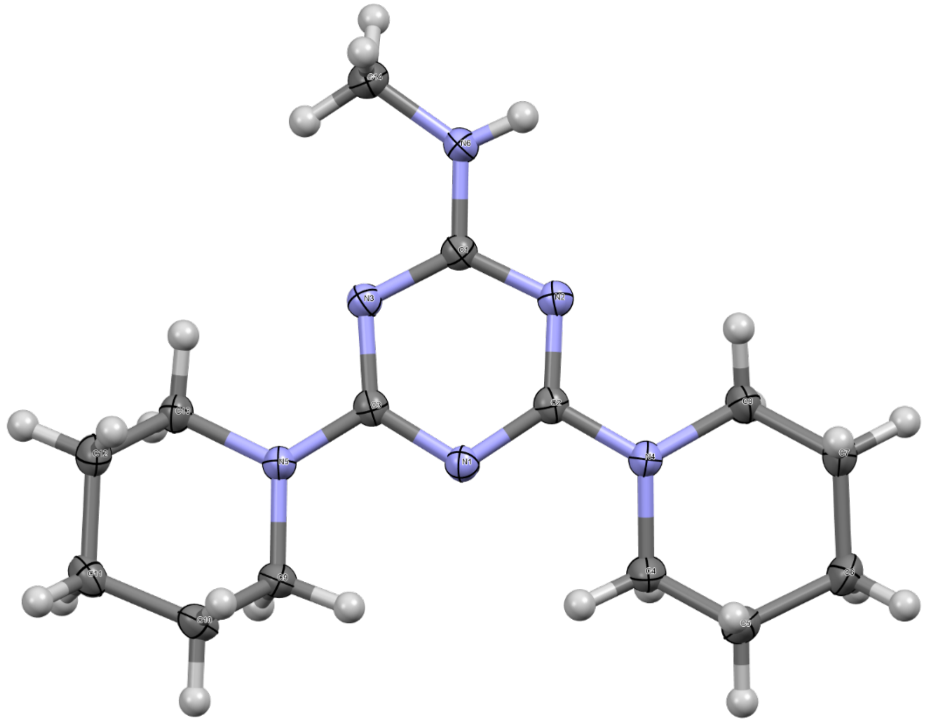

Figure S5. Molecular structure of **F1** with displacement ellipsoids at 50% probability for non-H atoms.

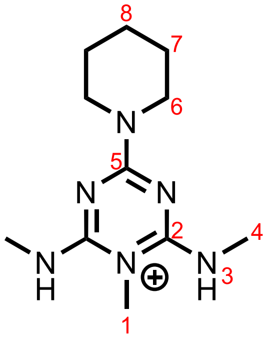

F2

^{1}H NMR (700 MHz, DMSO-d_6): δ_H = 8.17 (s, 2H, H_3), 3.79 (t, J = 5.5 Hz, 4H, H_6), 3.26 (s, 3H, H_1), 2.87 (s, 6H, H_4), 1.64 (p, J = 5.8 Hz, 2H, H_8), 1.53 (p, J = 5.9 Hz, 4H, H_7);

^{13}C NMR (176 MHz, DMSO-d_6): δ_C = 158.6 (C_5), 155.2 (C_2), 44.3 (C_6), 31.2 (C_1), 28.3 (C_4), 25.4 (C_7), 23.8 (C_8);

HRMS (ESI+): calc. for $[C_{11}H_{21}N_6]^+$ is 237.1822, found 237.1823;

FT-IR (ATR): ν_{max} /cm^{-1} 3214, 2942, 2857, 1649, 1595, 1538, 1490, 1447, 1413, 1333, 1300.

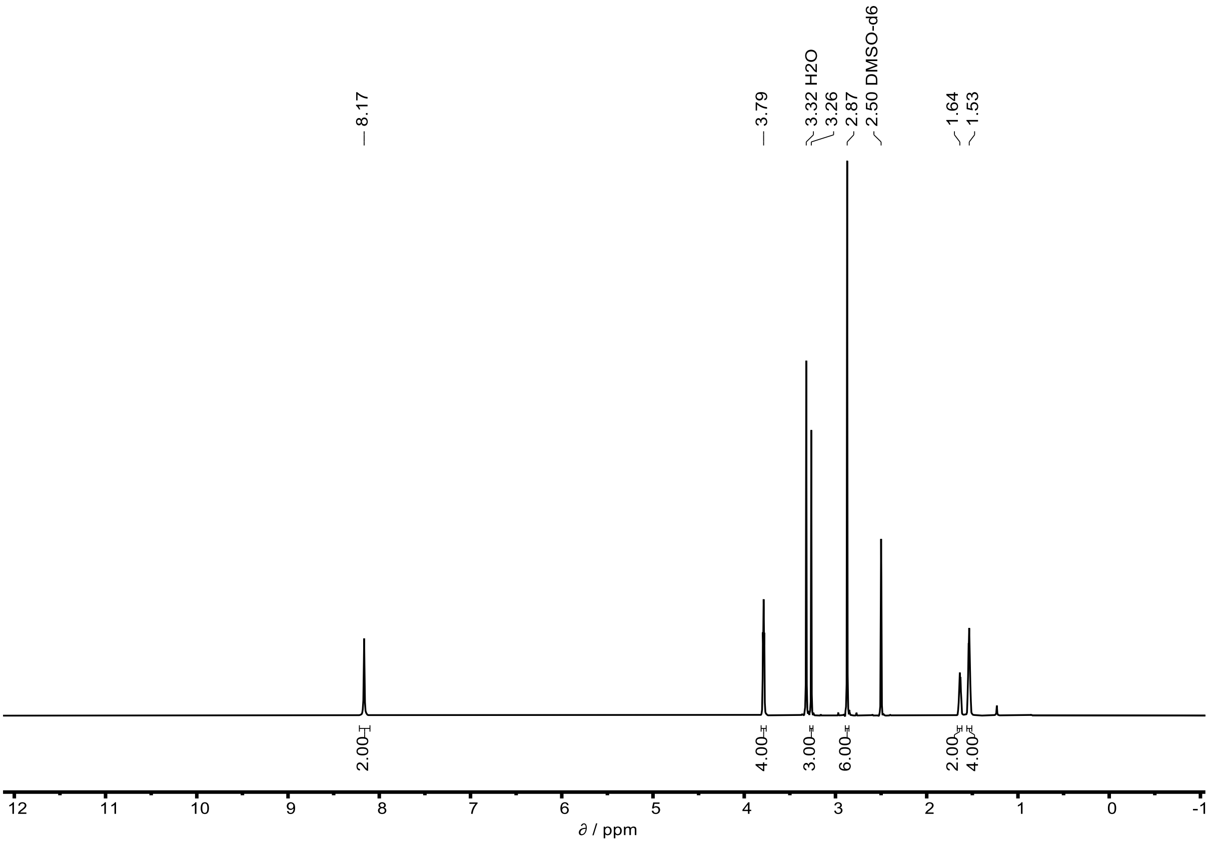

Figure S6. ^{1}H NMR spectrum (700 MHz, DMSO, 298 K) of **F2**.

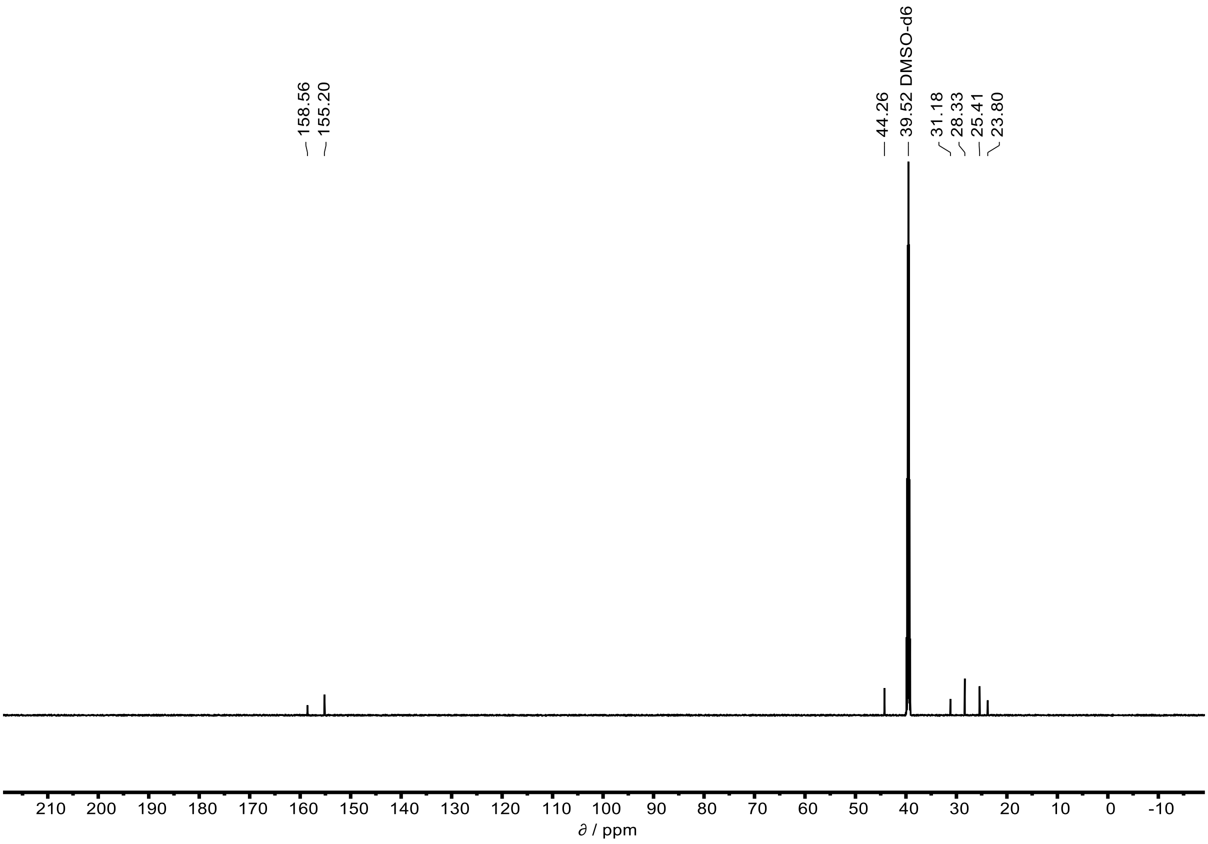

Figure S7. ^{13}C NMR spectrum (176 MHz, DMSO, 298 K) of **F2**.

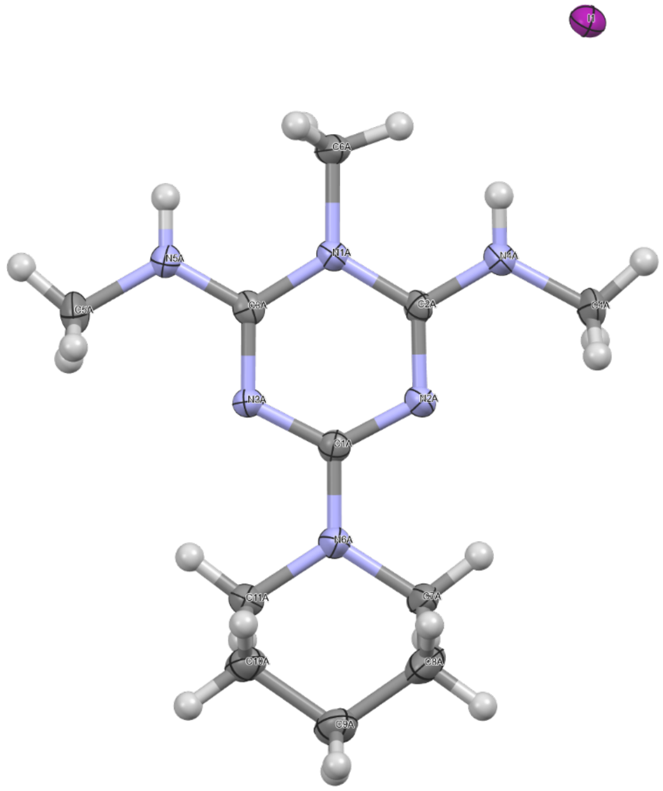

Figure S8. Molecular structure of **F2** with displacement ellipsoids at 50% probability for non-H atoms.

Table S1. Crystal structure and refinement details for compounds **F1** and **F2**.

Parameter	F1	F2
Empirical formula	$C_{14}H_{24}N_6$	$C_{11}H_{21}N_6I$
Formula weight	276.39	364.24
Temperature (K)	180(2)	180(2)
Crystal system	Orthorhombic	Orthorhombic
Space group	Pbca (No. 61)	$P\ 2_1\ 2_1\ 2_1$ (No. 19)
a (Å)	15.8764(7)	12.5319(7)
b (Å)	9.0853(4)	14.1229(8)
c (Å)	19.9252(9)	17.1943(9)
α (°)	90	90
β (°)	90	90
γ (°)	90	90
Volume ($Å^3$)	2874.0(2)	3043.2(3)
Z	8	8
Calc. density ($g\ cm^{-3}$)	1.278	1.590
F(000)	1200	1456
Crystal size (mm^3)	0.300 × 0.300 × 0.180	0.140 × 0.140 × 0.080
Radiation	Cu Kα (λ = 1.54178 Å)	Cu Kα (λ = 1.54178 Å)
2θ range for data collection (°)	8.88–133.07	8.10–133.26
Reflections collected	27,738	29,214
Independent reflections	2502	5340
Data / restraints / parameters	2502 / 0 / 187	5340 / 0 / 331
Goodness-of-fit on F^2	1.059	1.126
Final R values [I > 2σ(I)]	R_1 = 0.0369, wR_2 = 0.0963	R_1 = 0.0251, wR_2 = 0.0605
Final R values (all data)	R_1 = 0.0375, wR_2 = 0.0968	R_1 = 0.0254, wR_2 = 0.0606
Largest diff. peak/hole (e $Å^{-3}$)	0.358 / −0.216	0.772 / -0.511

Synthesis of 2

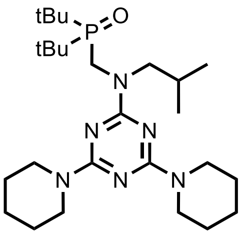

Compound **2** was synthesised according to the literature procedure.[2]

Synthesis of 3

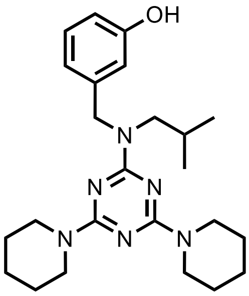

Compound **3** was synthesised according to the literature procedure.[2]

Synthesis of 4

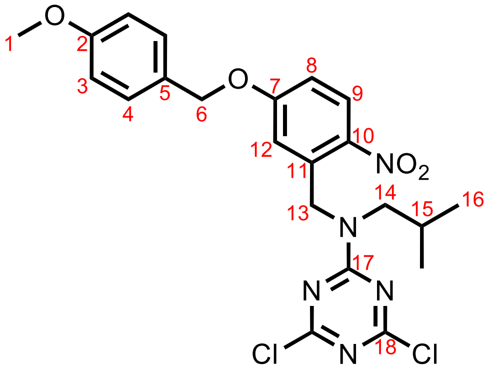

A solution of isobutylamine (25.75 mL, 259.1 mmol, 1.5 eq) and O-(*para*-methoxybenzyl)-3-formyl-4-nitrophenol[3] (49.60 g, 172.76 mmol, 1 eq) was stirred in dry DCM (350 mL) in the presence of molecular sieves (4 Å). After 4 hours, the solvent was removed *in vacuo,* and the residue was redissolved in MeOH (350 mL). The solution was cooled to 0 °C, and sodium borohydride (7.84 g, 207.3 mmol, 1.2 eq) was added in small portions. The resulting mixture was stirred at room temperature overnight. The reaction was quenched with H_2O (100 mL), and the organic solvents were removed *in vacuo*. The aqueous layer was extracted five times with EtOAc and the combined organic layers were washed with 0.1 M HCl solution and dried ($MgSO_4$) then the solvents were removed *in vacuo*. The resulting oil was redissolved in THF

(175 mL) and cooled to -78 °C. By cannula, the solution was added dropwise to a second flask containing cyanuric chloride (34.76 g, 190.0 mmol, 1.1 eq) and K_2CO_3 (25.42 g, 138.2 mmol, 2 eq) in THF (175 mL) at -78 °C. Following the addition, the mixture was stirred at -78 °C for 1 hour. The mixture was then filtered and the filtrate was concentrated. The residue was redissolved in EtOAc and washed three times with 0.1 M HCl solution and dried ($MgSO_4$). The solvent was removed *in vacuo* to afford the compound as a white solid (51.16 g, 104.2 mmol, 60%).

^{1}H NMR (700 MHz, Chloroform-*d*): δ_H = 8.25 (d, J = 9.1 Hz, 1H, H_9), 7.27 (d, J = 8.3 Hz, 2H, H_4), 7.00 (dd, J = 9.0, 2.7 Hz, 1H, H_8), 6.95 – 6.88 (m, 2H, H_3), 6.56 – 6.50 (m, 1H, H_{12}), 5.26 (s, 2H, H_{13}), 5.07 (s, 2H, C_6), 3.85 (d, J = 1.0 Hz, 3H, H_1), 3.40 (d, J = 7.5 Hz, 2H, H_{14}), 2.14 (n, J = 6.9 Hz, 1H, H_{15}), 0.97 – 0.96 (m, 6H, H_{16});

^{13}C NMR (176 MHz, Chloroform-*d*): δ_C = 170.4 (C_{18}), 166.0 (C_{17}), 163.3 (C_{10}), 159.9 (C_2), 141.1 (C_7), 135.1 (C_{11}), 129.0 (C_9), 128.9 (C_4), 127.2 (C_5), 114.4 (C_3), 113.7 (C_8), 113.6 (C_{12}), 70.7 (C_6), 55.4 (C_1), 55.2 (C_{14}), 49.5 (C_{13}), 27.0 (C_{15}), 20.14 (C_{16});

HRMS (ESI+): calc. for $[C_{22}H_{23}Cl_2N_5O_4 + H]^+$ is 492.1200 , found 492.1197;

FT-IR (ATR): ν_{max} /cm^{-1} 2961, 1556, 1510, 1477, 1436, 1390, 1322, 1287, 1229, 1168, 1138, 1080, 1062, 1031, 973, 843, 797, 733, 702, 646, 541.

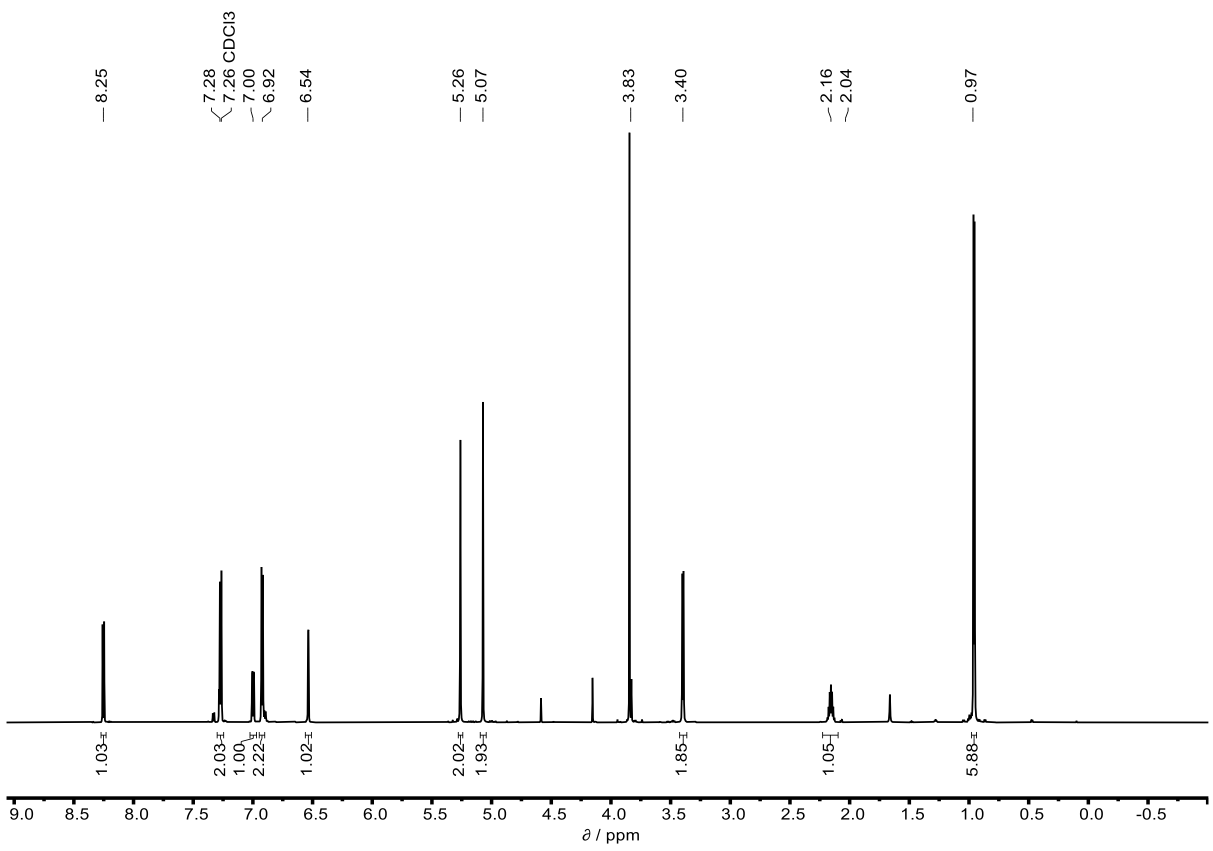

Figure S9. ^{1}H NMR spectrum (700 MHz, $CDCl_3$) of **4**.

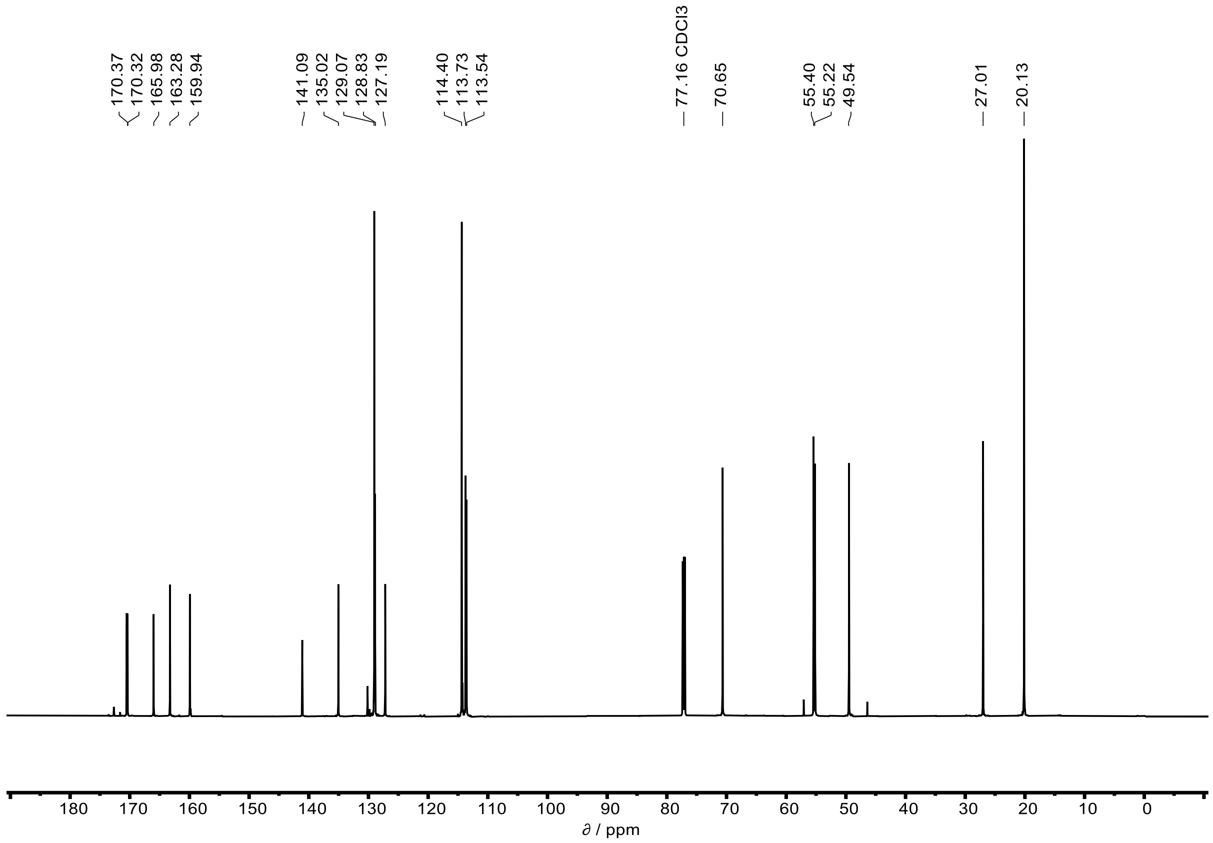

Figure S10. ^{13}C NMR spectrum (176 MHz, $CDCl_3$) of **4**.

Synthesis of 5

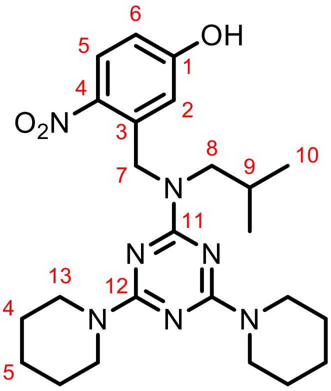

4 (199 mg, 0.40 mmol, 1.0 eq) was dissolved in THF (0.5 mL) and piperidine (2 mL) was added. The solution was heated in a microwave reactor at 80 °C for 20 minutes then the solvent was removed under a stream of N_2. The residues were dissolved in EtOAc and washed with 1M HCl solution twice and with brine once then dried ($MgSO_4$). The solvents were removed *in vacuo* then the residues were dissolved in CH_2Cl_2 (0.5 mL) and TFA (2 mL) and stirred for 1 hour. The solvent was removed under a stream of N_2. The residues were redissolved in EtOAc and washed with water once and with brine once then dried ($MgSO_4$). The crude was purified by reverse phase flash column chromatography (C4, A = water + 0.1% formic acid, B = MeCN + 0.1% formic acid, gradient of 30 – 60% B). The product was obtained as a white solid (154 mg, 0.328 mmol, 81%).

^{1}H NMR (400 MHz, Chloroform-*d*): δ_H = 8.02 (d, J = 9.0 Hz, 1H, H_5), 6.67 (d, J = 2.7 Hz, 1H, H_2), 6.60 (dd, J = 8.9, 2.7 Hz, 1H, H_6), 5.12 (s, 2H, H_7), 3.70 (s, 4H, H_{13}), 3.52 (s, 4H, H_{13}), 3.39 (d, J = 7.2 Hz, 2H, H_8), 2.10 (non, J = 6.8 Hz, 1H, H_9), 1.66 – 1.37 (m, 12H, $H_{14,15}$) 0.90 (d, J = 6.5 Hz, 6H, H_{10});

^{13}C NMR (101 MHz, Chloroform-*d*): δ_C = 167.9 (C_{12}), 164.7 (C_{11}), 162.1 (C_1), 140.5 (C_4), 139.0 (C_3), 128.3 (C_5), 114.8 (C_2), 114.1 (C_6), 55.1 (C_8), 49.2 (C_7), [44.9 and 44.6 (C_{13}, rotamers)], 27.8 (C_9), 25.8 (C_{14}), 24.9 (C_{15}), 20.6 (C_{10});

HRMS (ESI+): calcd. for $[C_{24}H_{35}N_7O_3 + H]^+$ is 470.2874, found 470.2885.

FT-IR (ATR): ν_{max} /cm^{-1} 2932, 2854, 1580, 1530, 1483, 1461, 1442, 1387, 1371, 1331, 1297, 1273, 1235, 1128,1098, 1071, 1023, 991, 845, 807, 736;

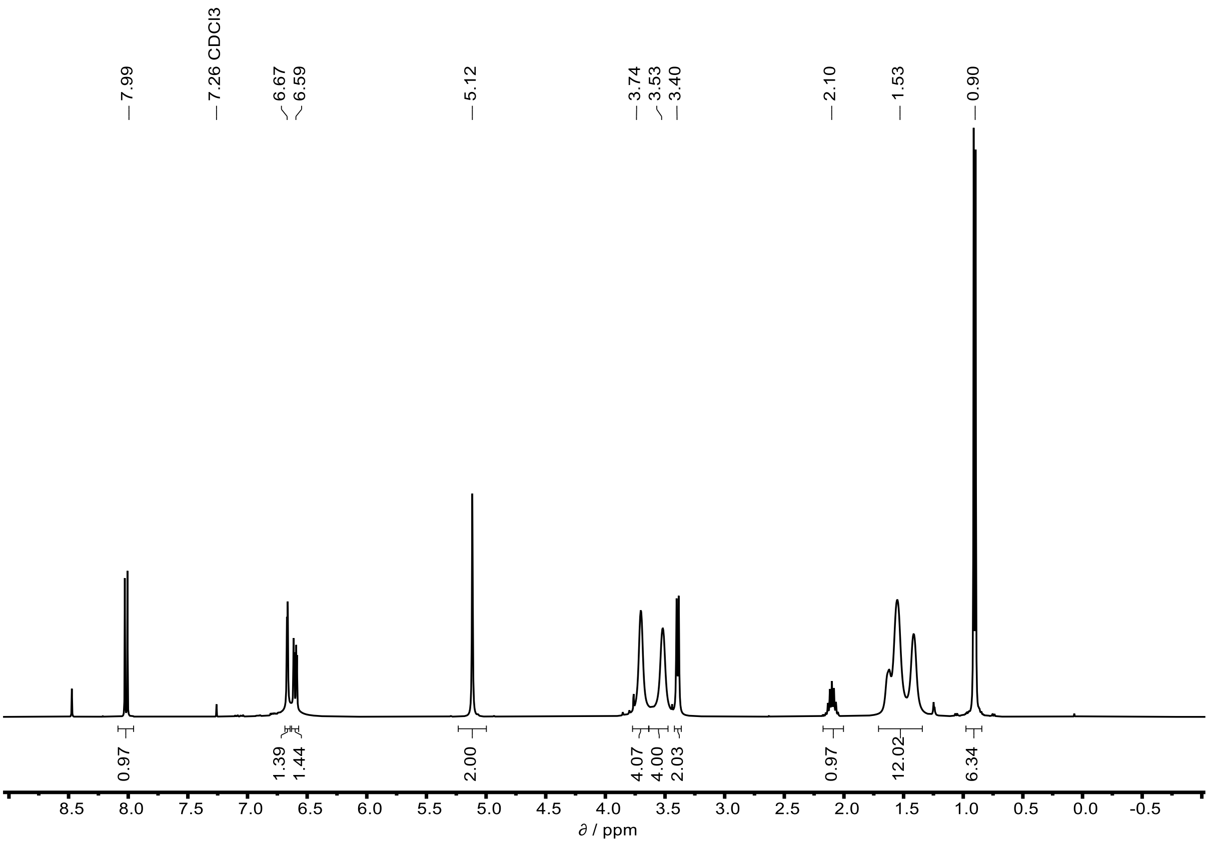

Figure S11. ^{1}H NMR spectrum (400 MHz, $CDCl_3$) of **5**.

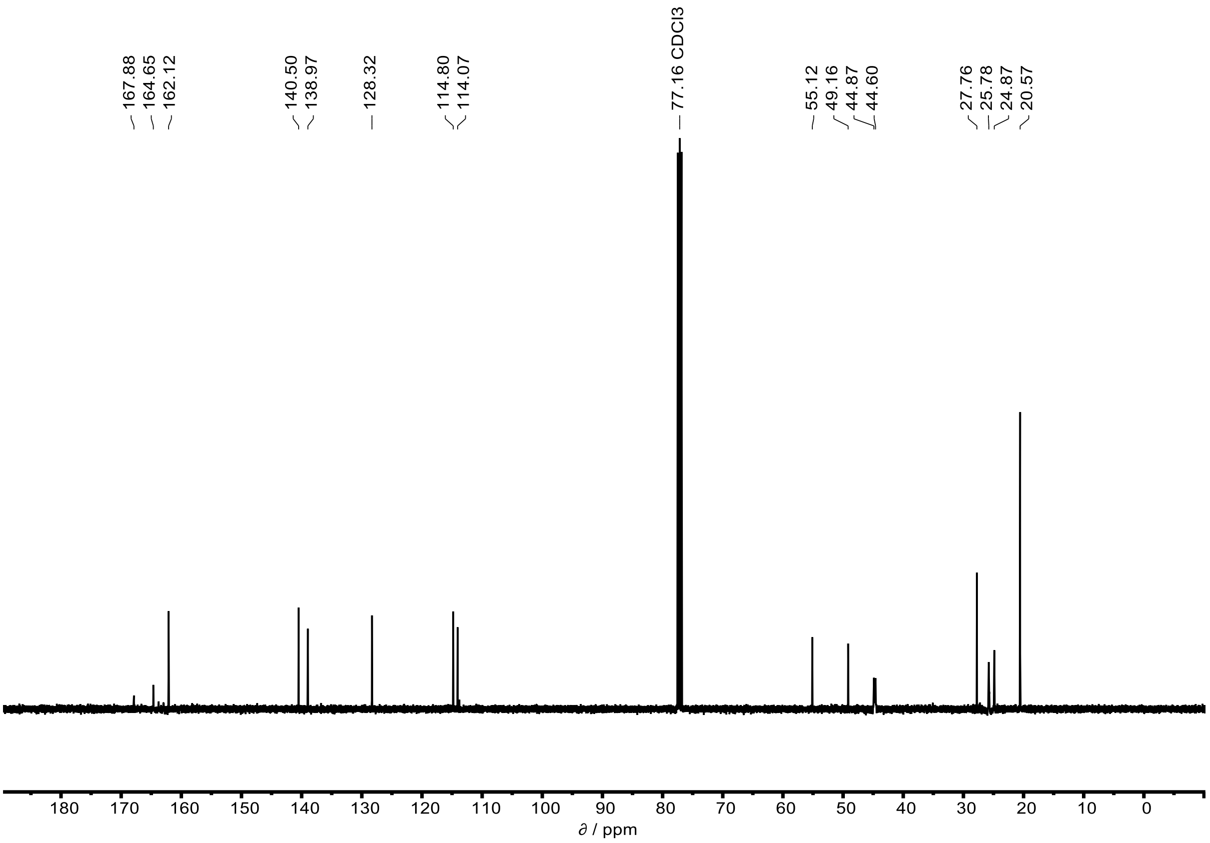

Figure S12. ^{13}C NMR spectrum (101 MHz, $CDCl_3$) of **5**.

Synthesis of Carboxylic Acid Dichlorotriazine

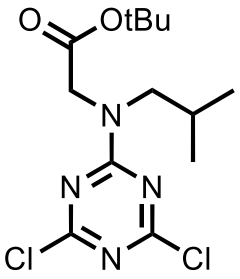

Carboxylic Acid Dichlorotriazine was synthesised according to the literature procedure.[4]

Synthesis of 6

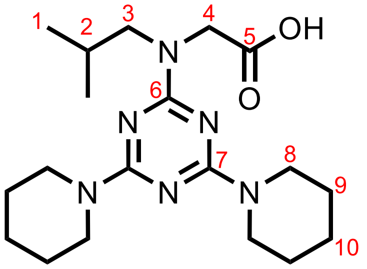

To a solution of **Carboxylic Acid Dichlorotriazine** (81.6 mg, 0.243 mmol, 1.0 eq) and DIPEA (170 µL, 0.97 mmol, 4.0 eq) in dry THF (3 mL) under an atmosphere of N_2 was added piperidine (95.5 µL, 0.97 mmol, 4.0 eq). The solution was heated under reflux for 15 hours and then the solvent was removed *in vacuo*. The residue was dissolved in EtOAc (20 mL) and washed with HCl (0.1 M in water, 3 x 10 mL). The combined aqueous layers were extracted with EtOAc (10 mL), dried ($MgSO_4$) and the solvent was removed *in vacuo*. The residues were dissolved in dry CH_2Cl_2 (2 mL) and TFA (130 µL) was added. The solution was stirred for 48 hours and the solvent was removed *in vacuo*. The residue was dissolved in EtOAc (20 mL) and washed with 0.1 M HCl solution, water, and brine then dried ($MgSO_4$). The solvent was removed *in vacuo* and the crude was purified by flash column chromatography (SiO_2, 0 – 80% EtOAc in Pet. Ether). The product was obtained as a white solid (75 mg, 0.200 mmol, 82%).

^{1}H NMR (500 MHz, $CDCl_3$) δ_H = 4.11 (s, 2H, H_4), 3.68 (m, 8H, H_8), 3.41 (s, 2H, H_3), 2.01 (m, 1H, H_2), 1.68 – 1.42 (m, 12H, $H_{9,10}$), 0.86 (d, J = 6.6 Hz, 6H, H_1);

^{13}C NMR (126 MHz, $CDCl_3$) δ_C = 165.3 ($C_{5,6,7}$), 164.2 ($C_{5,6,7}$), 56.7 (C_3), 53.9 (C_4), 44.5 (C_8), 27.8 (C_2), 25.9 (C_9), 25.0 (C_{10}), 20.3 (C_1);

HRMS (ESI+): calcd. for $[C_{19}H_{32}N_6O_2 + H]^+$ is 377.2660, found 377.2663;

FT-IR (ATR): ν_{max} /cm^{-1} 2952, 2935, 2853, 1536, 1486, 1461, 1442, 1290, 1273, 1246, 1237, 445.

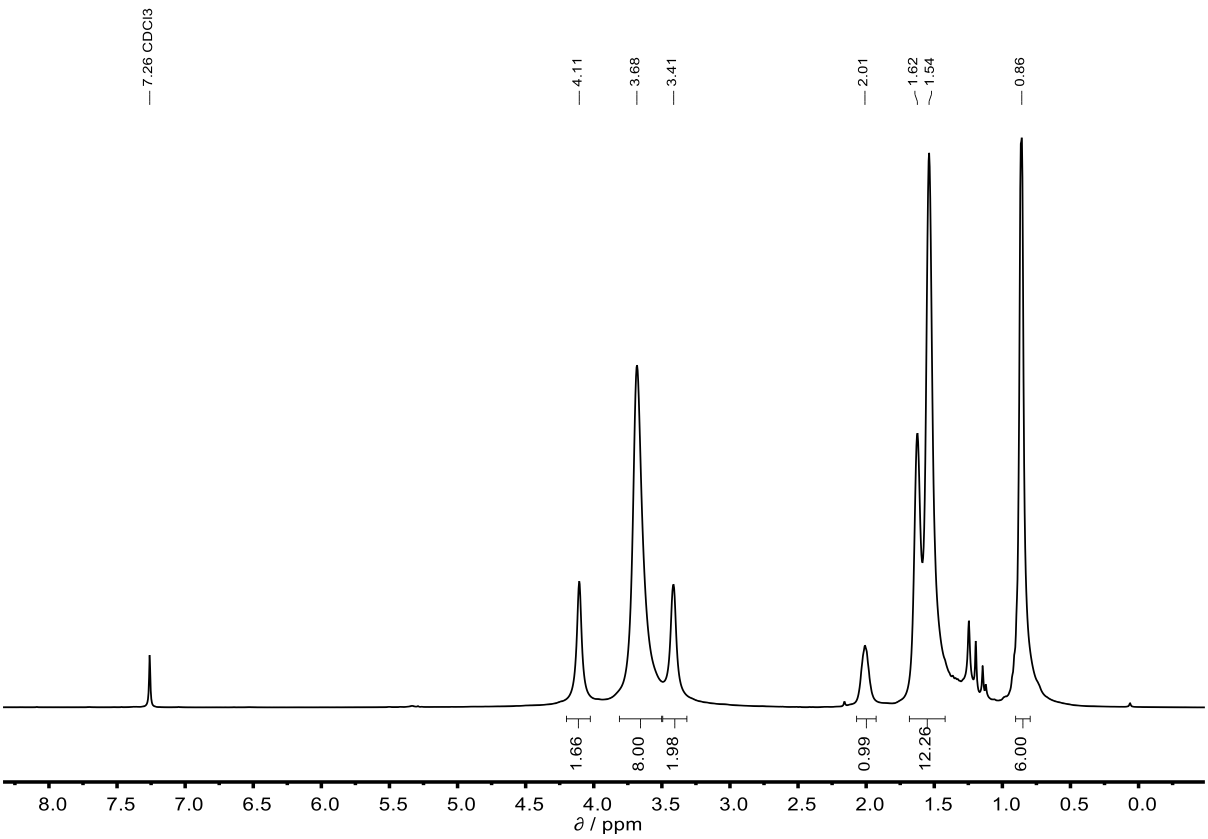

Figure S13. ^{1}H NMR spectrum (500 MHz, $CDCl_3$, 298 K) of **6**.

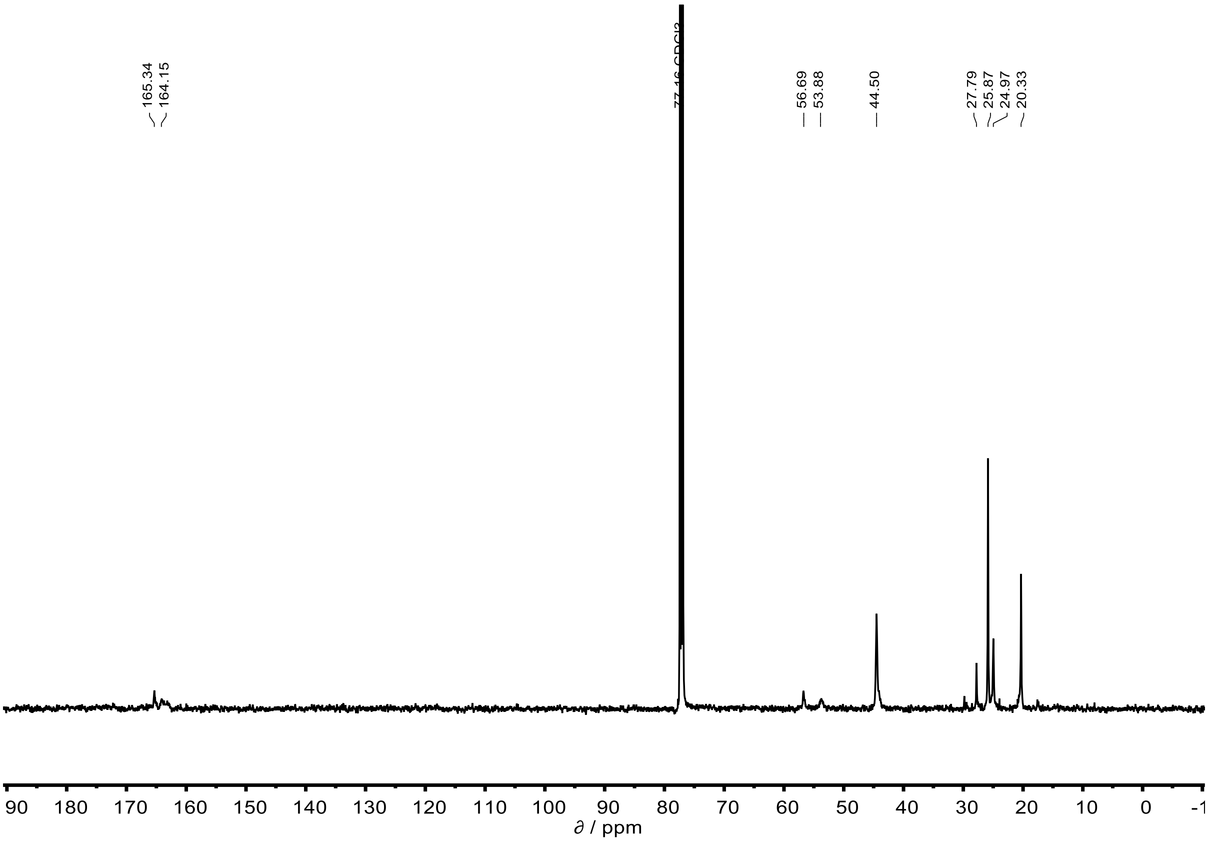

Figure S14. ^{13}C NMR spectrum (126 MHz, $CDCl_3$, 298 K) of **6**.

Synthesis of 7

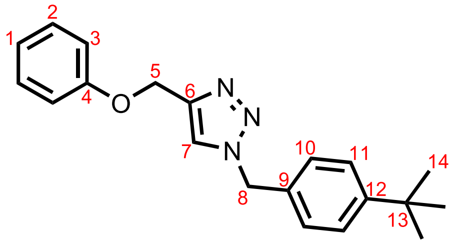

1-Phenoxy-2-propyne (60.2 mg, 0.46 mmol, 1.3 eq) was added to a solution of Copper(I) tetrakis(acetonitrile) hexafluorophosphate (169.4 mg, 0.53 mmol, 1.5 eq) in DMF. 4-tertbutylbenzylazide (67.6 mg, 0.36 mmol, 1.0 eq) was added to the reaction mixture and stirred. The reaction mixture was heated under microwave irradiation for 30 mins at 90°C. The reaction mixture was subsequently flushed with a stream of N_2 to remove DMF followed by dilution with EtOAc (40 mL). The mixture was washed with water, 1M HCl twice and EDTA (0.01 M, 1% NH_4OH). The organic layer was removed and the aqueous layer was extracted twice with EtOAc. The combined organic extracts were washed once again with EDTA (0.01 M, 1% NH_4OH), followed by 5% LiCl solution and brine then dried ($MgSO_4$). The solvent was removed *in vacuo* and the product was purified by flash column chromatography (SiO_2, 15 – 25% EtOAc in Pet. Ether). The product was obtained as a pale orange solid (71.9 mg, 0.22 mmol, 63%).

^{1}H NMR (500 MHz, Methanol-*d*$_4$): δ_H = 7.96 (s, 1H, H_7), 7.39 (d, *J* = 8.45 Hz, 2H, H_{11}), 7.21 – 7.27 (m, 4H, $H_{2,10}$), 6.96 (d, *J* = 8.75 Hz, 2H, H_3), 6.92 (tt, *J* = 7.35, 1.00 Hz, 1H, H_1), 5.53 (s, 2H, H_8), 5.10 (s, 2H, H_5), 1.29 (s, 9H, H_{14});

^{13}C NMR (126 MHz, Methanol-*d*$_4$): δ_C = 159.7 (C_4), 152.8 (C_{12}), 145.4 (C_6), 133.7 (C_9), 130.5 (C_2), 128.9 (C_{10}), 126.9 (C_{11}), 125.1 (C_7), 122.2 (C_1), 115.9 (C_3), 62.3 (C_5), 54.7 (C_8), 35.4 (C_{13}), 31.7 (C_{14});

FT-IR (ATR): ν_{max} / cm^{-1} 3125, 3081, 2956, 2919, 2867, 1600, 1586, 1497, 1465, 1454, 1439, 1402, 1301, 1247, 1210, 1172, 1129, 1077, 1053, 1041, 1017, 838, 813, 784, 750, 703, 689, 672, 572, 552, 510;

HRMS (ESI+): calculated for $[C_{20}H_{23}N_3O + H]^+$ is 322.1914, found is 322.1936.

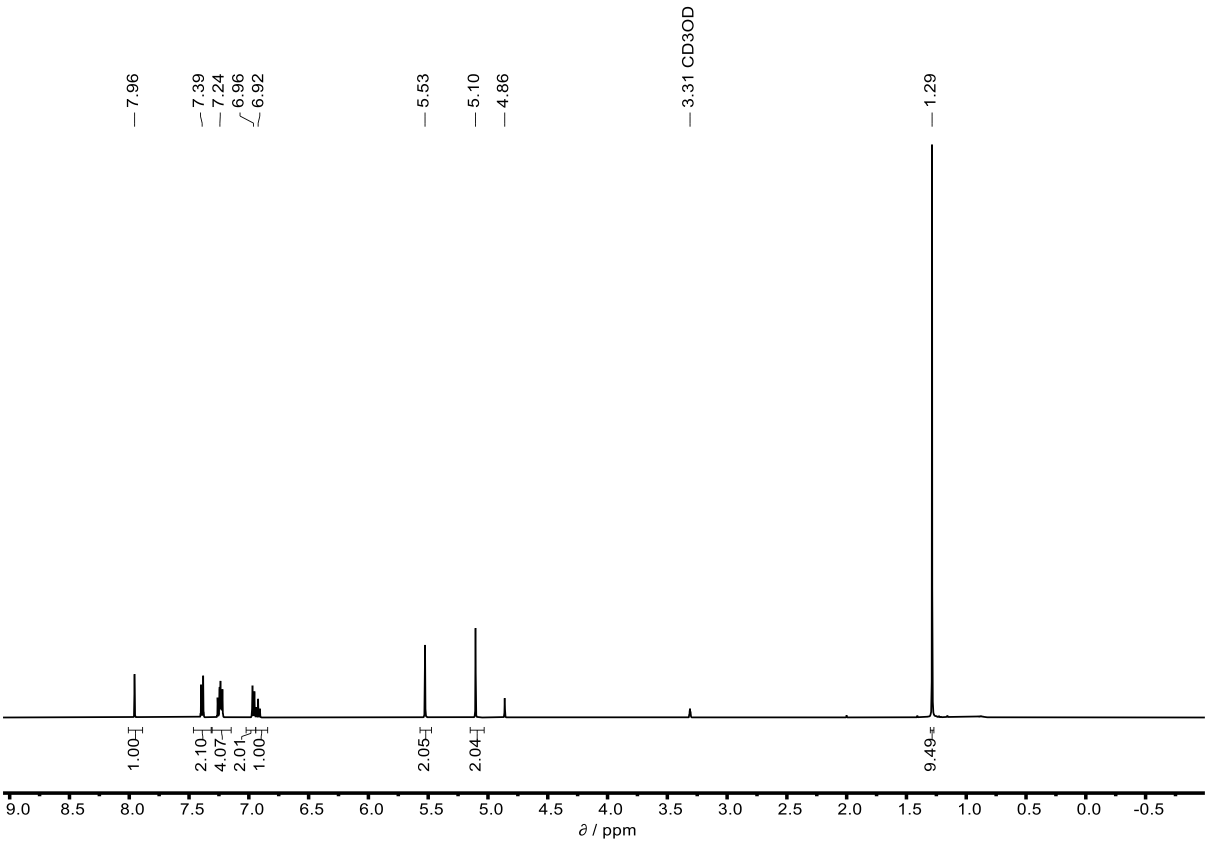

Figure S15. ^{1}H NMR spectrum (500 MHz, CD_3OD, 298 K) of **7**.

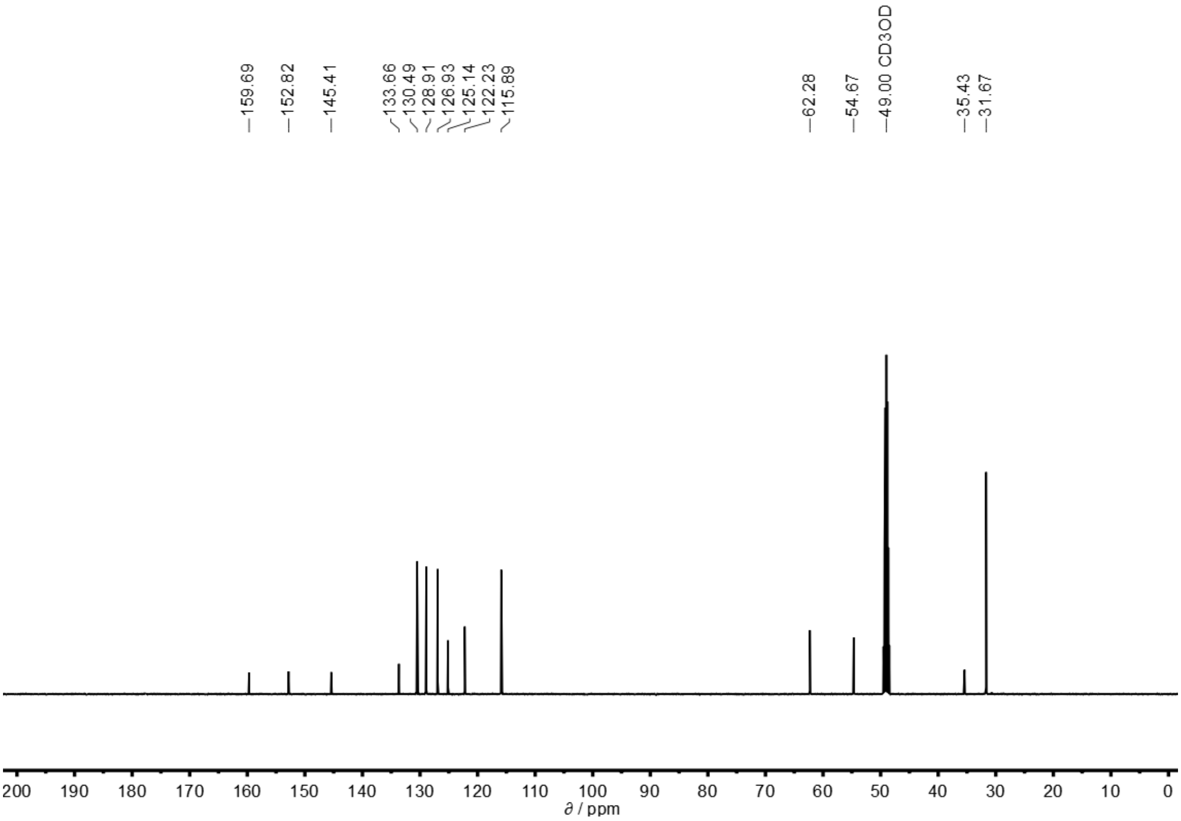

Figure S16. ^{13}C NMR spectrum (126 MHz, CD_3OD, 298 K) of **7**.

Synthesis of 8

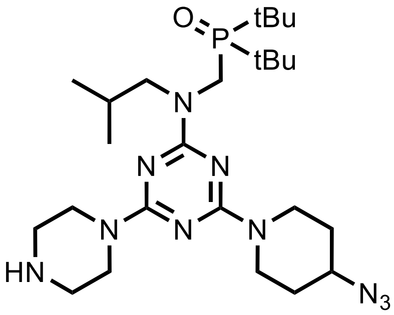

Compound **8** was synthesised according to the literature procedure.[3]

Synthesis of 9

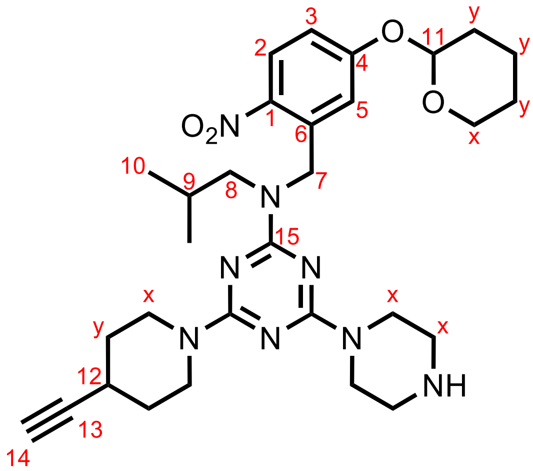

4 (472 mg, 1.04 mmol, 1.0 eq) was dissolved in THF and cooled to -78 °C. This was followed by dropwise addition of 4-ethynylpiperidinium trifluoroacetate (0.5 M solution in THF, 1 eq) and DIPEA (0.54 mL, 3.12 mmol, 3.0 eq) at -78 °C. The solution was allowed to warm to room temperature and stirred for 2 hours. A saturated solution of piperazine (2.0 M in THF, 8 eq) was added and then the solution was heated in a microwave reactor at 60 °C for 60 minutes. The solvent was removed *in vacuo* and the residues were dissolved in EtOAc and washed three times with water. The organic layer was dried ($MgSO_4$) then the crude was purified by flash column chromatography (SiO_2, 0 – 30% MeOH in CH_2Cl_2). **9** was obtained as a brown oil (336 mg, 0.58 mmol, 56%).

^{1}H NMR (500 MHz, CDCl$_3$): δ_H = 8.07 (d, J = 9.1 Hz, 1H, H_2), 6.97 (dd, J = 9.0, 2.7 Hz, 1H, H_3), 6.81 (d, J = 3.7 Hz, 1H, H_5), 5.37 (t, J = 3.4 Hz, 1H, H_{11}), 5.09 (s, 2H, H_7), 4.21 –2.73 (m, 16H, H_x and H_8), 2.66 – 2.50 (m, 1H), 2.64 and 2.52 (rotamers, m, 1H, H_{12}), 2.20 – 2.00 (m, 2H, $H_{9,14}$), 2.00 – 1.18 (m, 10H, H_y), 0.91 (d, J = 6.7 Hz, 6H, H_{10});

^{13}C NMR (126 MHz, CDCl$_3$): δ_C = 166.2 (C_{15}), 165.4 (triazine C), 165.1 (triazine C), 161.4 (C_4), 141.9 (C_1), 139.1 (C_6), 127.6 (C_2), 116.1 and 116.0 (rotamers, C_5), 113.8 and 113.8 (rotamers, C_3), 96.7 (C_{11}), 87.0 (C_{13}), 69.4 and 69.2 (rotamers, C_{14}), 62.5 (C_x of THP), 54.9 (C_8), 49.0 (C_7), 45.7 (C_x), 45.5 (C_x), 43.7 (C_x), 43.6 (C_x), 41.8 (C_x), 41.8 (C_x), 31.4 (C_y), 31.2 (C_y), 30.0 (C_y), 27.8 (C_9), 27.4 (C_y), 27.3 (C_y), 25.0 (C_y), 20.7 (C_{10}), 18.7 (C_y);

FT-IR (ATR): ν_{max} / cm^{-1} 3307, 2948, 2927, 2852, 1531, 1482, 1430, 1313, 1282, 1255, 1241, 1202, 903, 728;

HRMS (ESI+): calculated for $[C_{30}H_{42}N_8O_4 + H]^+$ is 579.3402, found is 579.3418.

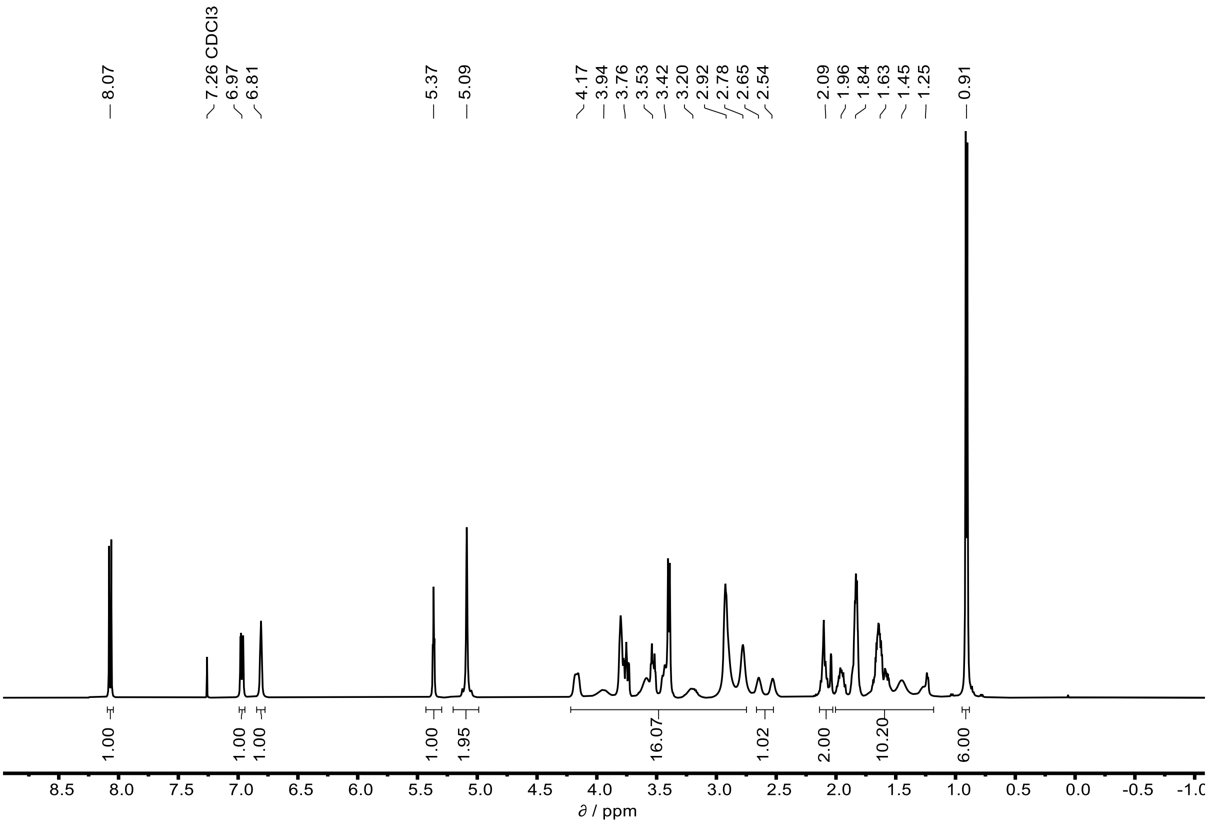

Figure S17. ^{1}H NMR spectrum (500 MHz, $CDCl_3$, 298 K) of **9**.

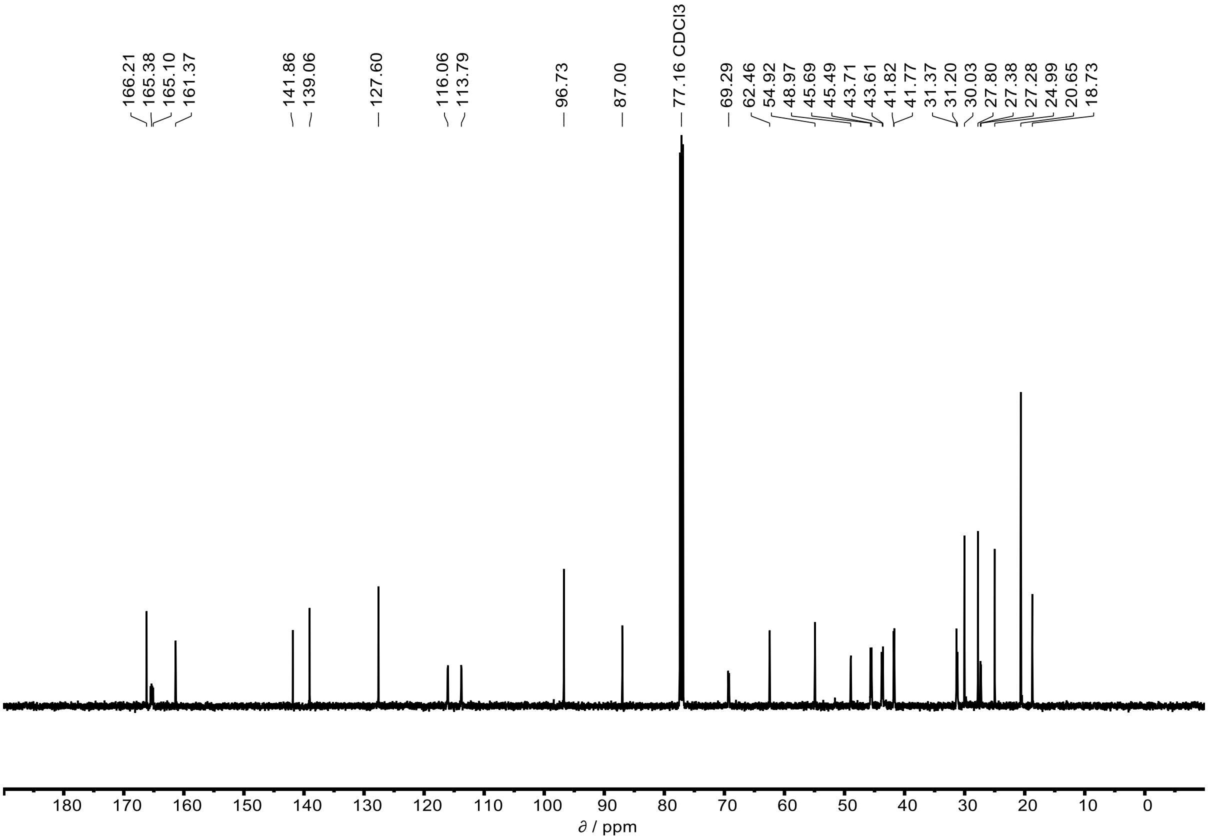

Figure S18. ^{13}C NMR spectrum (126 MHz, $CDCl_3$, 298 K) of **9**.

Synthesis of yDDAz

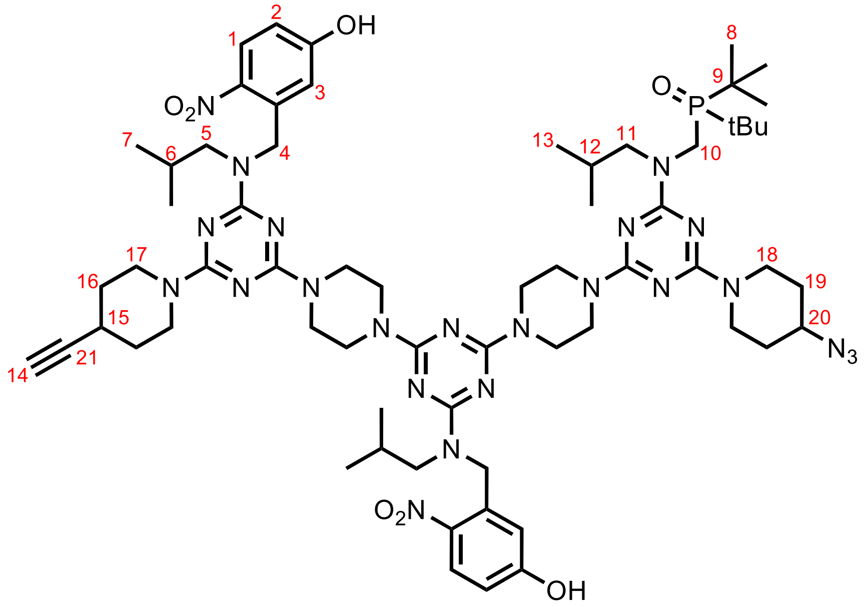

4 (51.3 mg, 0.11 mmol, 1 eq) was dissolved in THF (1.12 mL) and cooled to -78 °C. This was followed by the dropwise addition of **9** (0.5 M solution in THF, 1 eq) and DIPEA (39.2 µL, 0.23 mmol, 3 eq) at -78 °C. The temperature of the resulting mixture was raised to 15°C and stirred for 2 hours. The solvent was removed *in vacuo.* The remaining residue was dissolved in EtOAc and washed with water (3 times). The organic layer was dried ($MgSO_4$) and the solvent was removed *in vacuo* to yield a REMO 2-mer which was used in the next step without further purification.

The crude REMO 2-mer and **8** (181.3 mg, 0.33 mmol, 3 eq) were dissolved in THF (0.45 mL). The mixture was heated in a microwave reactor at 60°C for 60 minutes. The solvent was removed *in vacuo.* The residue was dissolved in EtOAc and washed with water (3 times). The organic layer was dried ($MgSO_4$) and the solvent was removed *in vacuo*. The crude product was partially purified by flash chromatography (SiO_2, 0 – 40% MeOH in CH_2Cl_2). This crude was then dissolved in a mixture of MeOH (0.5 mL), CH_2Cl_2 (0.5 mL) and TFA (0.1 mL) and stirred for 2 hours. The solvent was removed under a stream of nitrogen then the crude was purified by flash column chromatography (SiO_2, 0 – 10% MeOH in CH_2Cl_2). **yDDAz** was obtained as a yellow foam (26.1 mg, 0.020 mmol, 19%).

^{1}H NMR (700 MHz, $CDCl_3$): δ_H = 8.19 – 7.95 (m, 2H, H_1), 7.07 – 6.58 (m, 4H, $H_{2,3}$), 5.36 – 4.82 (m, 4H, H_4), 4.62 – 4.30 (m, 2H, H_{10}), 4.31 – 4.14 (m, 2H, H_{18}), 4.05 – 3.26 (m, 29H, $H_{5,11,17,18,20}$ and protons of piperazine rings), 2.82 – 2.63 (m, 1H, H_{15}), 2.20 – 2.07 (m, 4H, $H_{6,12,14}$), 1.96 – 1.86 (m, 3H, $H_{16,19}$), 1.81 – 1.68 (m, 2H, $H_{16,19}$), 1.68 – 1.51 (m, 3H, $H_{16,19}$), 1.34 – 1.27 (m, 18H overlaps with grease, H_8), 1.01 – 0.80 (m, 18H, overlaps with grease, $H_{7,13}$);

^{13}C NMR (176 MHz, $CDCl_3$): δ_C = 164.2 – 161.0, 139.6, 128.8 – 128.3 (C_1), 117.24, 115.7 – 114.1 ($C_{2,3}$), 85.5 (C_{21}), 70.4 (C_{14}), 57.8, 56.1, 55.7, 49.8 (C_4), 44.7 – 42.3 (carbons of piperazines), 41.2 (C_{20}), 35.9 (d, $^{1}J_{PC}$ = 54.3 Hz, C_9), 30.9, 30.7, 29.8, 27.8 ($C_{6,12}$), 26.7 (C_8), 26.5 (C_{15}), 20.6 ($C_{7,13}$), 20.42 ($C_{7,13}$);

^{31}P NMR (162 MHz, $CDCl_3$): δ_P = 64.0;

FT-IR (ATR): ν_{max} / cm^{-1} 2959, 2093, 1673, 1613, 1536, 1483, 1434, 1310, 1254, 1201, 1139, 995, 807, 729;

HRMS (ESI+): calculated for $[C_{64}H_{94}N_{23}O_7P + H]^+$ is 1328.7522, found is 1328.7529.

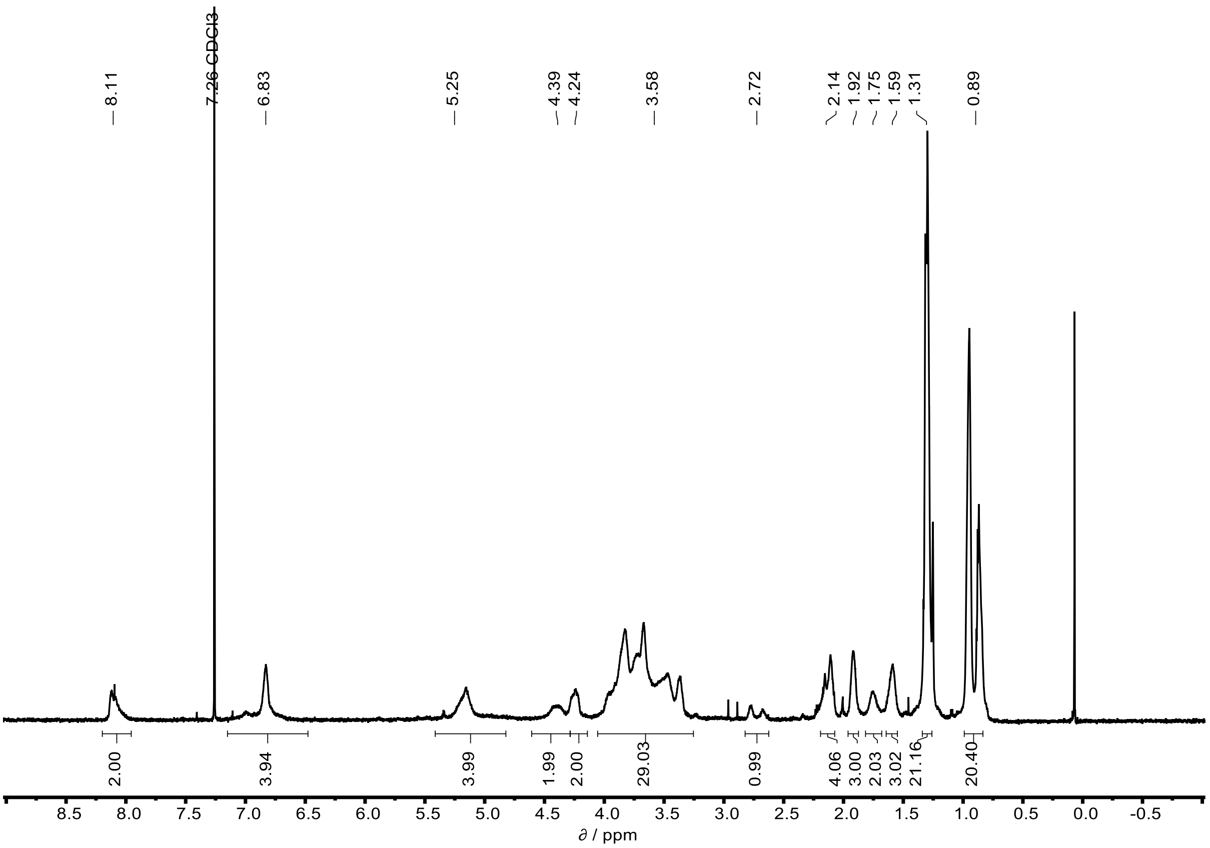

Figure S19. ^{1}H NMR spectrum (700 MHz, $CDCl_3$, 298 K) of **yDDAz**.

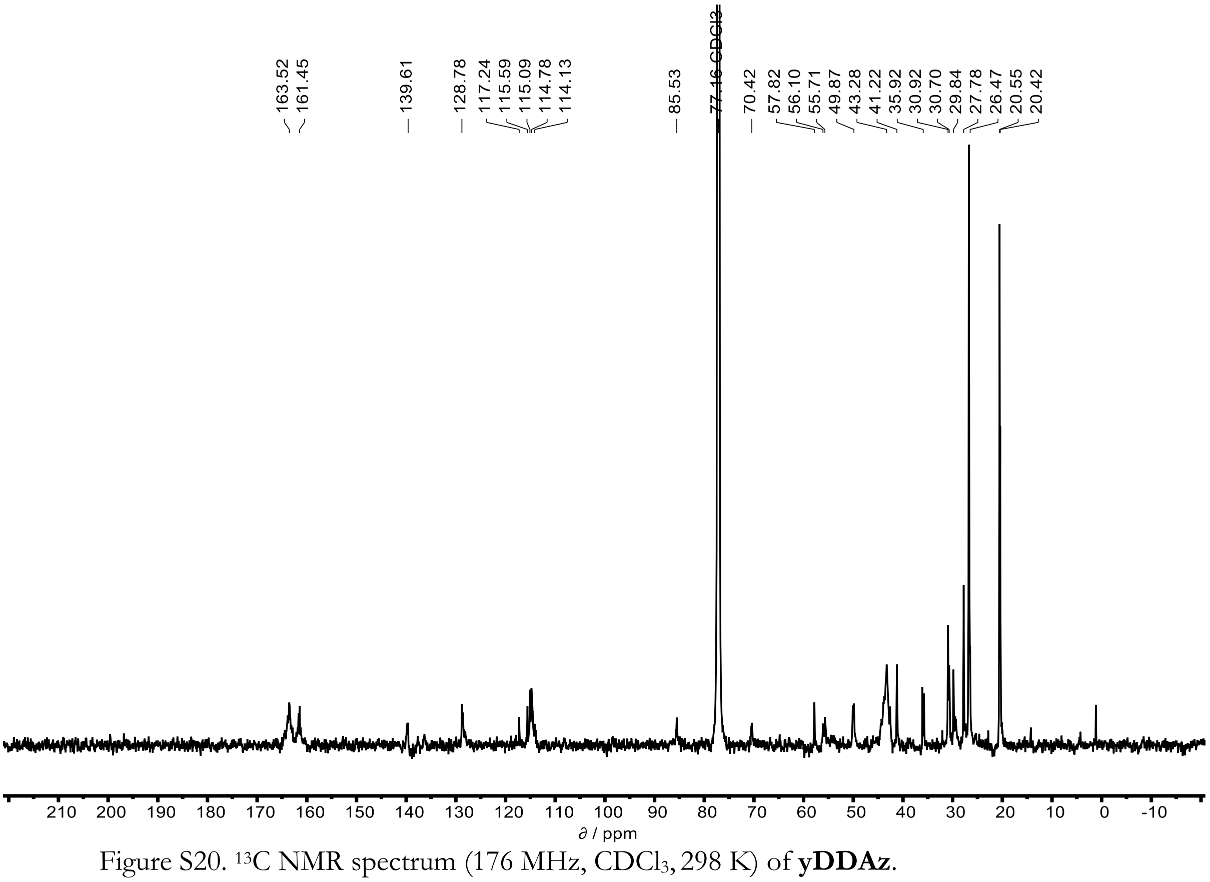

Figure S20. ^{13}C NMR spectrum (176 MHz, $CDCl_3$, 298 K) of **yDDAz**.

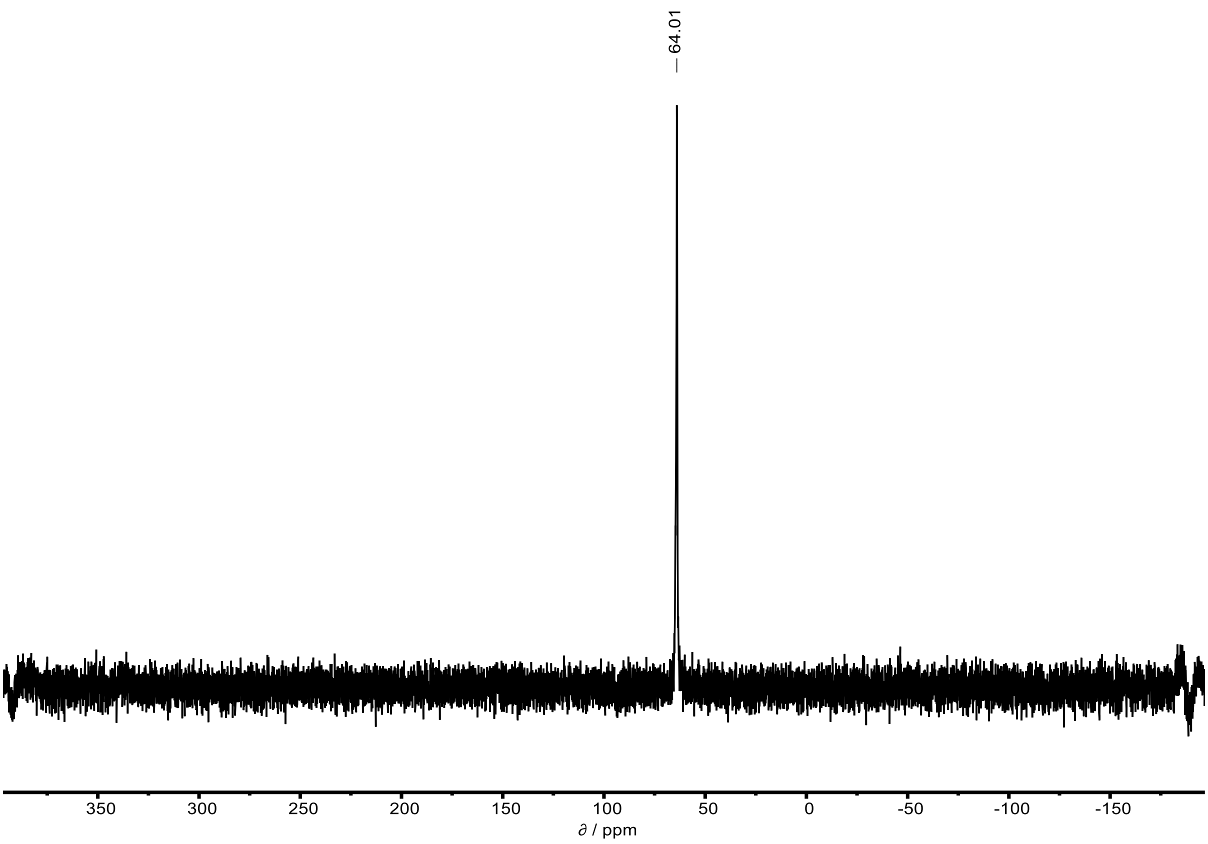

Figure S21. ^{31}P NMR spectrum (162 MHz, $CDCl_3$, 298 K) of **yDDAz**.

Synthesis of yDAOOOOADy*

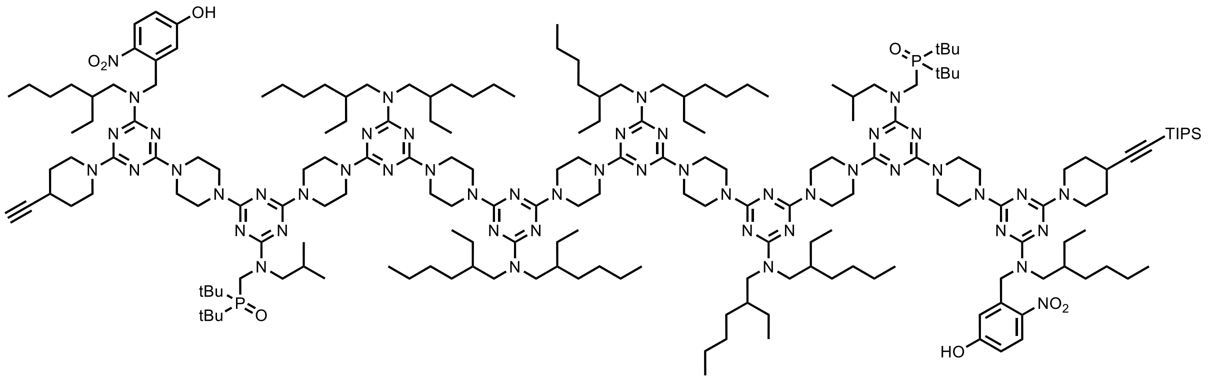

yDAOOOOADy* was synthesised according to the literature procedure.[5]

Synthesis of pAADp

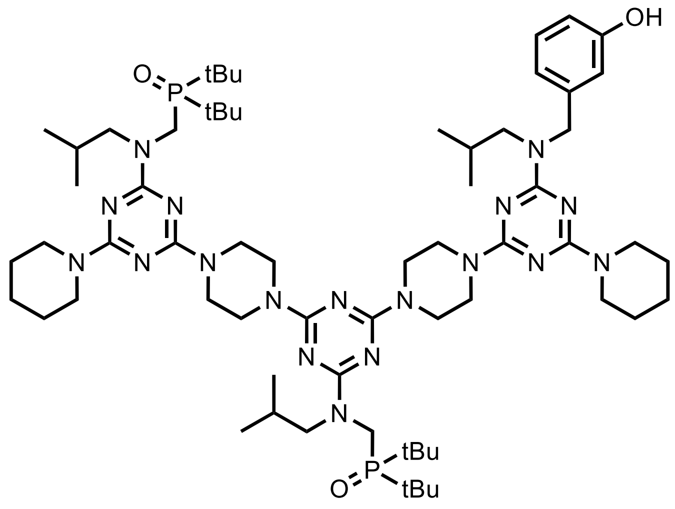

Compound **pAADp** was synthesised according to the literature procedure.[2]

b. Time Course of Methylation

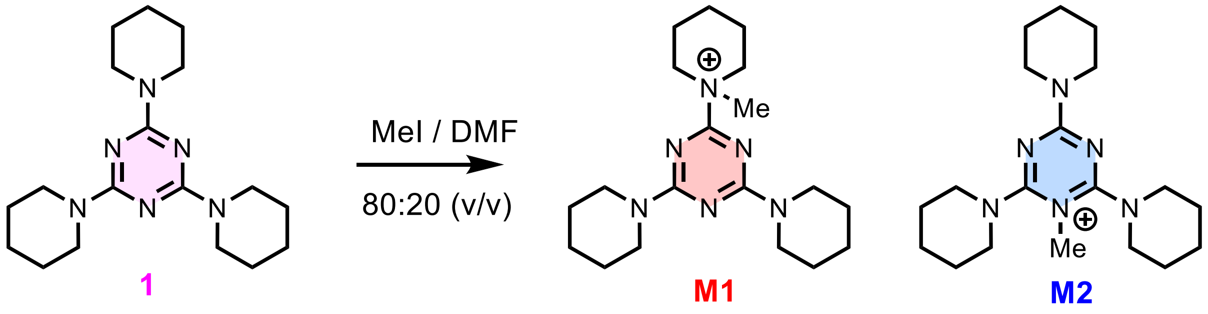

Compound **1** (5.7 mg, 17 µmol) was dissolved in methyl iodide (0.8 mL) and DMF (0.2 mL) in a sealed vial and stirred at room temperature. Periodically, 10 µL aliquots of the reaction mixture were removed, and MeOH (1.0 mL) was added. 0.1 mL of the resultant mixture was removed from this solution and MeOH (0.9 mL) was added. This was analysed by UPLC with an injection volume of 2.0 µL. UPLC conditions: C18 column at 40 °C; water + 0.1 % formic acid (A) and acetonitrile + 0.1 % formic acid (B); gradient of 5 – 100% B over three minutes. The integrals of the 254 nm UV trace were calculated using the built-in integration function in the MassLynx Software.

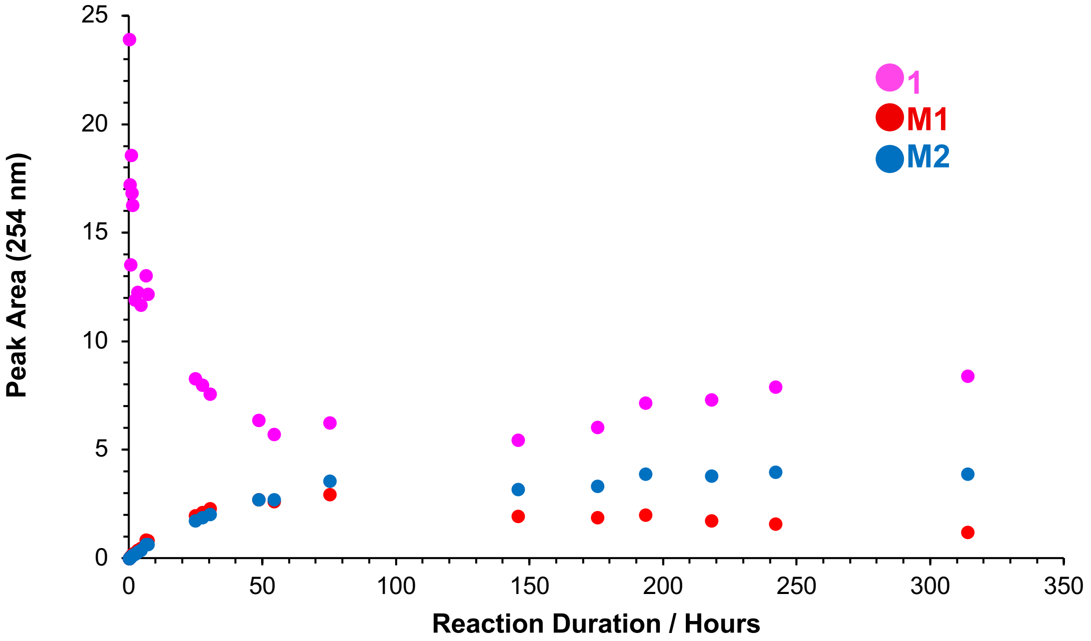

Figure S22. Product distribution, quantified by the UPLC peak areas (254 nm), of the methylation reaction of compound **1** as a function of the reaction duration. The maximum yield of methylated compounds is achieved at approximately three days.

c. Nucleophile Screening

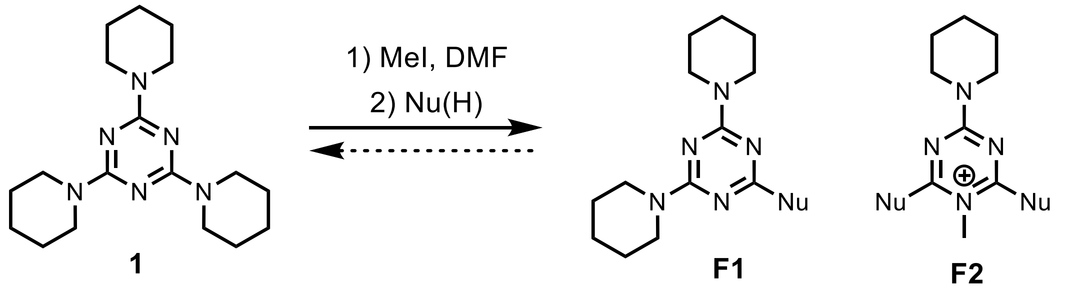

General procedure for screening nucleophiles for the fragmentation reactions of **M1** and **M2**:

Compound **1** was dissolved in methyl iodide (0.8 mL) and DMF (0.2 mL) in a sealed vial and stirred at room temperature for three days, after which the volatiles were removed under a stream of N_2. The resulting mixture of **1**, **M1** and **M2** (approx. 3 mg total) was dissolved in a solution of a nucleophile (1 mL) until complete consumption of **M1** and **M2**. When elevated temperatures were desired, microwave irradiation was used.

The reaction mixtures were then analysed by UPLC-MS, and the UPLC peak areas (254 nm) were used to qualitatively estimate the relative abundances of products. In most cases, three major products were observed: compound **1**, and fragments of the type **F1** and **F2**. In some cases, additional products were observed but not identified. Table S2 shows the results of the screening. The optimal conditions, which gave the highest total yield of **F1** and **F2,** was methylamine (33 wt.% in ethanol) at room temperature and so this was chosen for all subsequent REMO fragmentation experiments.

Table S2. Qualitative product distributions observed when a mixture of **1**, **M1** and **M2** was reacted under different nucleophilic conditions. Key: **+++** = Very High Yield; **++** = High Yield; **+** = Low Yield; (+) = Trace Yield; **-** = Not observed.

**Nu(H) =**	**Solvent**	**T / °C**	**Product**			
			**F1**	**F2**	**1**	**Other**
$MeNH_2$	EtOH	25	+++	++	+	**-**
$BnNH_2$	Neat[i]	25	+	++	+	**-**
	Neat[i]	80	**(+)**	+	+++	**-**
	EtOH	25	++	+	+	**(+)**
Propargyl Amine	Neat[i]	25	++	+	+	+
	Neat[i]	80	+	+	+++	+
	EtOH	25	++	+	+	+
$^nPrNH_2$	Neat[i]	25	++	+	+	+
NH_3	MeOH	25	+	+	++	+
Et_2NH	Neat[i]	80	**-**	**-**	+++	+
BnNHMe	Neat	90	+	**-**	+++	+
$^iPr_2NH$	Neat[i]	75	**(+)**	**-**	+++	+
LiOH	Water/ Dioxane[i]	120	+	++	++	+++
NaOMe	Dry MeOH[i]	25	+	+	++	+++

[i] The mixture of **1**, **M1** and **M2** was washed with pet. ether to remove some **1** before treatment with nucleophile, but this does not change the conclusions of these qualitative screening experiments.

d. HPLC Kinetics Study

Compound **1** was dissolved in methyl iodide (0.8 mL) and DMF (0.2 mL) in a sealed vial and stirred at room temperature for three days. An aliquot of the reaction mixture was flushed with a stream of N_2 for 1 h to remove the DMF and methyl iodide, then redissolved in CH_2Cl_2 (0.2 mL) before being flushed with a stream of N_2 for 1 h again. The reaction mixture was redissolved in $MeNH_2$ solution (33 wt% in EtOH, 1.0 mL) to mark the start of the reaction, after which the reaction mixture was analysed periodically by HPLC until no further change was observed.

HPLC details

The HPLC method for each run is described in Figure S23.

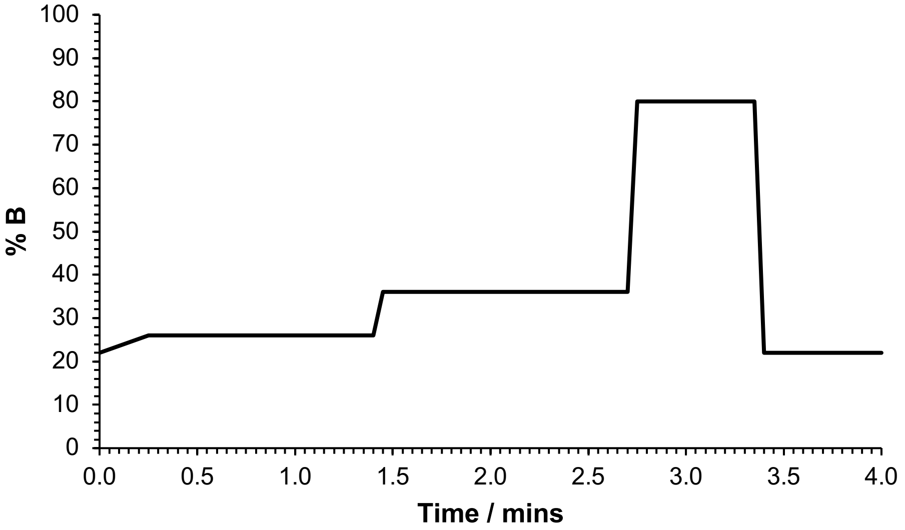

Figure S23. HPLC method for each injection during the kinetic study of the fragmentation of **1**, **M1** and **M2** in methylation solution. The A solvent was water, the B solvent was acetonitrile, the stationary phase was a CORTECS C8 column at 22 °C, the flow rate was 1.7 mL min^{-1}, and the injection volume was 1 μL.

Kinetic Modelling details

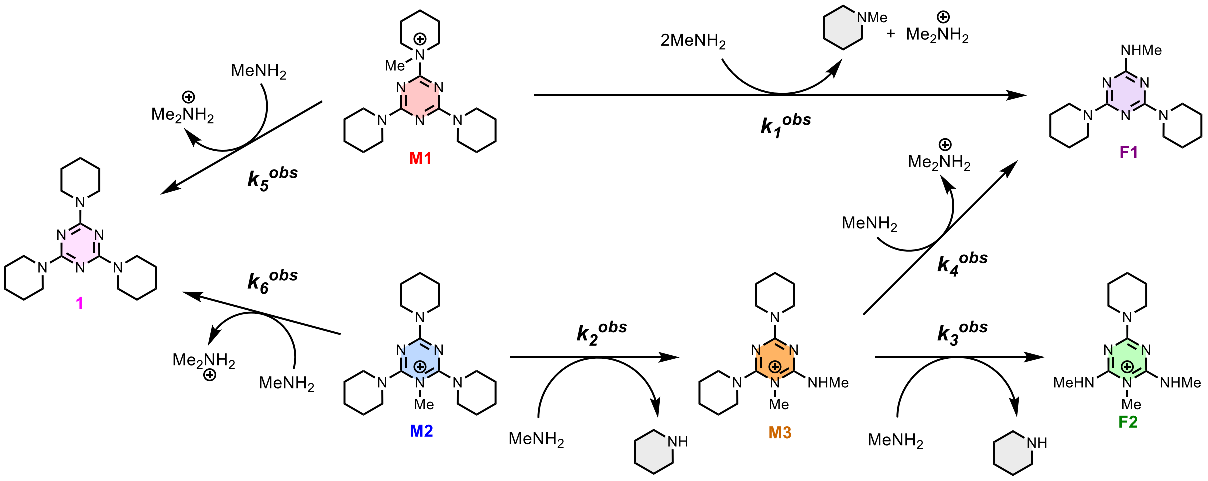

Figure S24. Reaction pathways occurring when a mixture of **1**, **M1** and **M2** is dissolved in methylamine solution (33 wt.% in ethanol) at 298 K. Each reaction arrow is labelled with its observed pseudo-first-order rate constant.

Derivation of Equations

All of the reactions (shown in Figure S24) are pseudo-first-order because methylamine is present in a large excess ($k_i^{obs} = k_i\,[\mathrm{MeNH_2}]$). Therefore, the following differential equations are established:

$$\frac{\mathrm{d}[\mathbf{1}]}{\mathrm{d}t} = k_5^{\mathrm{obs}}[\mathbf{M1}] + k_6^{\mathrm{obs}}[\mathbf{M2}] \qquad ((1))$$

$$\frac{\mathrm{d}[\mathbf{M1}]}{\mathrm{d}t} = -(k_1^{\mathrm{obs}} + k_5^{\mathrm{obs}})[\mathbf{M1}] \qquad ((2))$$

$$\frac{\mathrm{d}[\mathbf{M2}]}{\mathrm{d}t} = -(k_2^{\mathrm{obs}} + k_6^{\mathrm{obs}})[\mathbf{M2}] \qquad ((3))$$

$$\frac{\mathrm{d}[\mathbf{M3}]}{\mathrm{d}t} = k_2^{\mathrm{obs}}[\mathbf{M2}] - (k_3^{\mathrm{obs}} + k_4^{\mathrm{obs}})[\mathbf{M3}] \qquad ((4))$$

$$\frac{\mathrm{d}[\mathbf{F1}]}{\mathrm{d}t} = k_1^{\mathrm{obs}}[\mathbf{M1}] + k_4^{\mathrm{obs}}[\mathbf{M3}] \qquad ((5))$$

$$\frac{\mathrm{d}[\mathbf{F2}]}{\mathrm{d}t} = k_3^{\mathrm{obs}}[\mathbf{M3}] \qquad ((6))$$

Equations ((2)) and ((3)) can be solved to give expressions for [**M1**] and [**M2**]:

$$[\mathbf{M1}] = [\mathbf{M1}]_0 \exp\{-(k_1^{\mathrm{obs}} + k_5^{\mathrm{obs}})t\} \qquad ((7))$$

$$[\mathbf{M2}] = [\mathbf{M2}]_0 \exp\{-(k_2^{\mathrm{obs}} + k_6^{\mathrm{obs}})t\} \qquad ((8))$$

Where $[\mathbf{M1}]_0$ and $[\mathbf{M2}]_0$ are the initial concentrations of **M1** and **M2**, respectively, and t is the reaction duration.

Equations ((7)) and ((8)) are substituted into equation ((1)) to give an expression for [**1**]:

$$[\mathbf{1}] = [\mathbf{1}]_0 + \frac{k_5^{\mathrm{obs}}[\mathbf{M1}]_0}{k_1^{\mathrm{obs}}+k_5^{\mathrm{obs}}}\{1 - \exp\{-(k_1^{\mathrm{obs}} + k_5^{\mathrm{obs}})t\}\} + \frac{k_6^{\mathrm{obs}}[\mathbf{M2}]_0}{k_2^{\mathrm{obs}}+k_6^{\mathrm{obs}}}\{1 - \exp\{-(k_2^{\mathrm{obs}} + k_6^{\mathrm{obs}})t\}\} \quad ((9))$$

Equation ((8)) is substituted into equation ((4)), which is then solved to give an expression for [**M3**]:

$$[\mathbf{M3}] = \frac{k_2^{\mathrm{obs}}[\mathbf{M2}]_0}{(k_3^{\mathrm{obs}} + k_4^{\mathrm{obs}}) - (k_2^{\mathrm{obs}} + k_6^{\mathrm{obs}})}\{\exp\{-(k_2^{\mathrm{obs}} + k_6^{\mathrm{obs}})t\} - \exp\{-(k_3^{\mathrm{obs}} + k_4^{\mathrm{obs}})t\}\} \quad ((10))$$

Finally, substitution of equations ((7)) and ((10)) into ((5)) and ((6)) gives expressions for [**F1**] and [**F2**]:

$$[\mathbf{F1}] = \frac{k_1^{\mathrm{obs}}[\mathbf{M1}]_0}{k_1^{\mathrm{obs}}+k_5^{\mathrm{obs}}}\{1 - \exp\{-(k_1^{\mathrm{obs}} + k_5^{\mathrm{obs}})t\}\} + \frac{k_2^{\mathrm{obs}}k_4^{\mathrm{obs}}[\mathbf{M2}]_0}{(k_3^{\mathrm{obs}}+k_4^{\mathrm{obs}})-(k_2^{\mathrm{obs}}+k_6^{\mathrm{obs}})}\left(\frac{1}{k_2^{\mathrm{obs}}+k_6^{\mathrm{obs}}} - \frac{1}{k_3^{\mathrm{obs}}+k_4^{\mathrm{obs}}} + \frac{\exp\{-(k_3^{\mathrm{obs}}+k_4^{\mathrm{obs}})t\}}{k_3^{\mathrm{obs}}+k_4^{\mathrm{obs}}} - \frac{\exp\{-(k_2^{\mathrm{obs}}+k_6^{\mathrm{obs}})t\}}{k_2^{\mathrm{obs}}+k_6^{\mathrm{obs}}}\right) \quad ((11))$$

$$[\mathbf{F2}] = \frac{k_2^{\mathrm{obs}}k_3^{\mathrm{obs}}[\mathbf{M2}]_0}{(k_3^{\mathrm{obs}}+k_4^{\mathrm{obs}})-(k_2^{\mathrm{obs}}+k_6^{\mathrm{obs}})}\left(\frac{1}{k_2^{\mathrm{obs}}+k_6^{\mathrm{obs}}} - \frac{1}{k_3^{\mathrm{obs}}+k_4^{\mathrm{obs}}} + \frac{\exp\{-(k_3^{\mathrm{obs}}+k_4^{\mathrm{obs}})t\}}{k_3^{\mathrm{obs}}+k_4^{\mathrm{obs}}} - \frac{\exp\{-(k_2^{\mathrm{obs}}+k_6^{\mathrm{obs}})t\}}{k_2^{\mathrm{obs}}+k_6^{\mathrm{obs}}}\right) \quad ((12))$$

Fitting of Equations to Data

The peaks in each HPLC run were integrated at 254 nm. When two peaks overlapped substantially, the data was exported into Excel, where a linear correction was applied to the baseline and the two peaks were fitted to two Gaussian curves. It was assumed that the absorbance of each compound follows the Beer-Lambert Law such that the concentrations are linearly proportional to the integral of the HPLC peaks *i.e.* $\mathrm{I}_{\mathbf{X}} = A_{\mathbf{X}}[\mathbf{X}]$ where $\mathrm{I}_{\mathbf{X}}$ is the peak integral, $A_{\mathbf{X}}$ is the constant of proportionality and [**X**] is the concentration of species **X**).

Firstly, equations ((7)) and ((8)) were solved graphically, as shown in Figure S25.

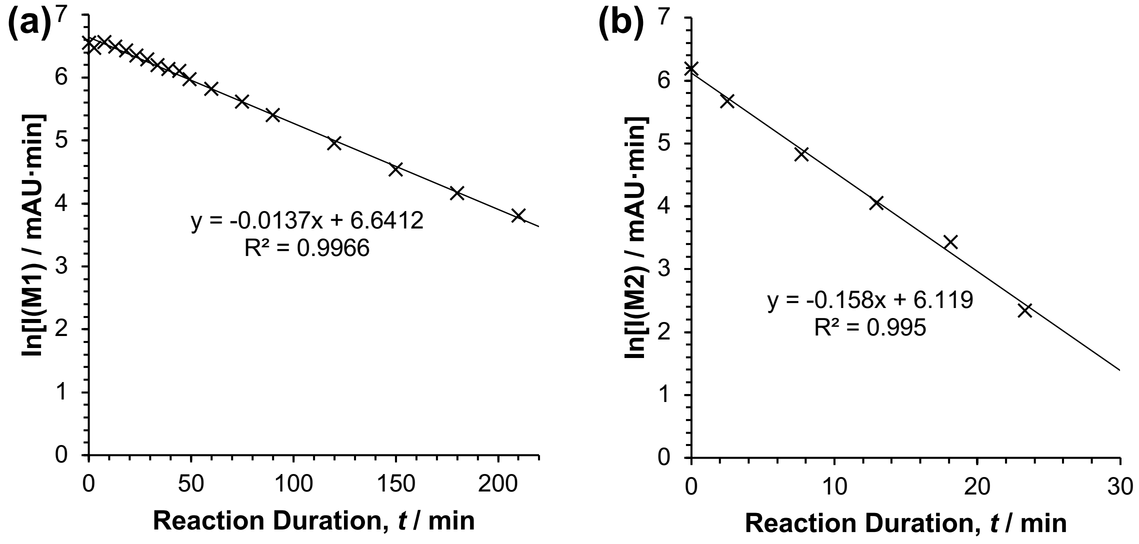

Figure S25. Plots of the natural logarithm of the integral of (a) the **M1** HPLC peak and (b) the **M2** HPLC peak against the reaction duration, *t*. The lines of best fit give the values $(k_1^{\mathrm{obs}} + k_5^{\mathrm{obs}})$ = 0.0137 min^{-1} and $(k_2^{\mathrm{obs}} + k_6^{\mathrm{obs}})$ = 0.0158 min^{-1}.

The rest of the parameters were obtained by iteratively fitting equations ((9 – 12)) to the experimental HPLC peak areas using the scipy.optimize() function in Python. The values of the fitted parameters are summarised in Table S3, along with their respective errors and the data used to determine them.

Table S3. The values of the fitted parameters, their errors and the data used for their acquisition, as defined in Figure S24.

Parameter	Units	Value	Error	Data Used
$A_{\mathbf{1}}[\mathbf{1}]_0$	mAU·min	368	24	**1**
$A_{\boldsymbol{M1}}[\mathbf{M1}]_0$	mAU·min	791	-	**M1**
$A_{\boldsymbol{M2}}[\mathbf{2b}]_0$	mAU·min	428	-	**M2**
$A_{\mathbf{1}}[\mathbf{M1}]_0 k_5^{\mathrm{obs}}$	mAU	2.6	0.2	**1**
$A_{\mathbf{1}}[\mathbf{M2}]_0 k_6^{\mathrm{obs}}$	mAU	66	5	**1**
$A_{\boldsymbol{F1}}[\mathbf{M1}]_0 k_1^{\mathrm{obs}}$	mAU	17.2	0.4	**F1**
$A_{\mathbf{I}}[\mathbf{M2}]_0 k_2^{\mathrm{obs}}$	mAU	74	6	**M3**
$A_{\boldsymbol{F1}}[\mathbf{M2}]_0 k_2^{\mathrm{obs}} k_4^{\mathrm{obs}}$	mAU min^{-1}	2.1	0.2	**F1**
$A_{\boldsymbol{F2}}[\mathbf{M2}]_0 k_2^{\mathrm{obs}} k_3^{\mathrm{obs}}$	mAU min^{-1}	5.08	0.08	**F2**
$k_1^{\mathrm{obs}} + k_5^{\mathrm{obs}}$	min^{-1}	0.0137	0.0002	**M1**
$k_2^{\mathrm{obs}} + k_6^{\mathrm{obs}}$	min^{-1}	0.158	0.006	**M2**
$k_3^{\mathrm{obs}} + k_4^{\mathrm{obs}}$	min^{-1}	0.0748	0.0012	**F2**

Evidence for Demethylation of the Intermediate

To confirm that demethylation of the intermediate (**M3**) was occurring (process with rate constant k_4^{obs}), the equations were again fitted to the experimental HPLC peak areas but with the constraint of $k_4^{obs} = 0$ min^{-1}. The resulting fit was significantly worse than when the intermediate demethylation was included in the model – this was particularly evident in the fit of the species **F1**, as shown in Figure S26.

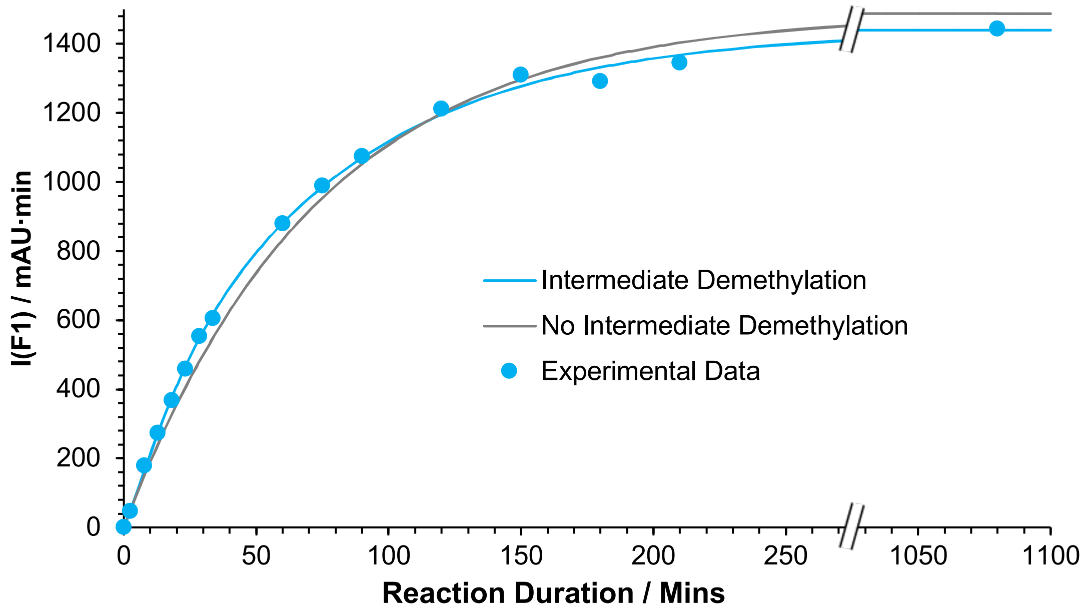

Figure S26. Comparison of the experimental HPLC peak areas ($\mathbf{I}_{\mathbf{F1}}$) for species **F1** with the prediction of the kinetic modelling, with and without the inclusion of an intermediate demethylation process into the model.

e. General Sequencing Procedure

The general procedure followed for all sequencing experiments, unless otherwise noted, is as follows:

The REMO was dissolved in methyl iodide solution (500 µL, 80% in DMF by volume) and stirred in a sealed vial for three days. The excess methyl iodide and DMF were removed by flushing under a stream of nitrogen, redissolving in CH_2Cl_2 then again flushing with nitrogen. Then the residues were redissolved in methylamine solution (500 µL, 33 wt.% in EtOH) and the solution was stirred overnight. The excess methylamine and EtOH were removed by flushing under a stream of nitrogen. The residues were then dissolved in THF/MeOH and further diluted to approx. 20 µM concentration in THF. This solution was then subjected to UPLC-MS (ESI+) analysis.

UPLC conditions:
ACQUITY UPLC BEH C4 Column, 300 Å, 1.7 µm, 2.1 mm × 50 mm;
Solvent A: Water + 0.1% Formic acid; Solvent B: THF + 0.1% Formic acid;
Gradient of 0 - 4 minutes 30% - 100% B then 2 minutes 100% B;
Flow rate: 0.4 ml/min;
Column temperature of 40 °C;
Injection volume of 1-10 µL.

f. Additional Investigations

Methylation duration for a trimer

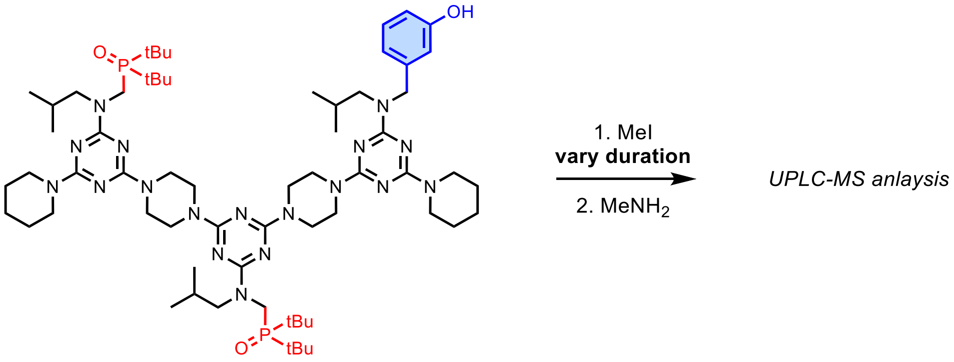

To check that three days of methylation was optimal for the sequencing of REMO, the 3-mer **pAADp** (7.9 mg) was subjected to the general sequencing procedure, using 1.5 mL of methyl iodide solution. Periodically, 50 µL aliquots were removed, flushed with nitrogen and stirred in methylamine solution overnight.. These samples were then flushed with nitrogen, diluted and analysed by UPLC-MS, as per the general procedure.

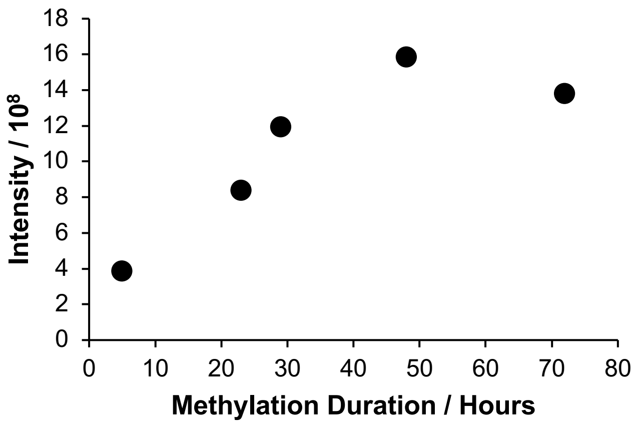

Figure S27. The effect of varying the duration of the methylation step on the total fragment intensity in the sequence determination of REMO 3-mer **pAADp**. Intensity is the sum of the extracted-ion intensities of each of the expected fragments of **pAADp**.

Extracted-ion chromatograms for each of the expected fragments were produced (m/z ± 0.5) and the intensities of each of these chromatograms was summed together to obtain a metric for the confidence in the sequence determination. The results are shown in Figure S27, which confirm that a methylation period of approximately 2-3 days is optimal for sequencing REMO, which is in agreement with the studies performed on the model compound **1** (see Figure S22).

Detection limit investigation

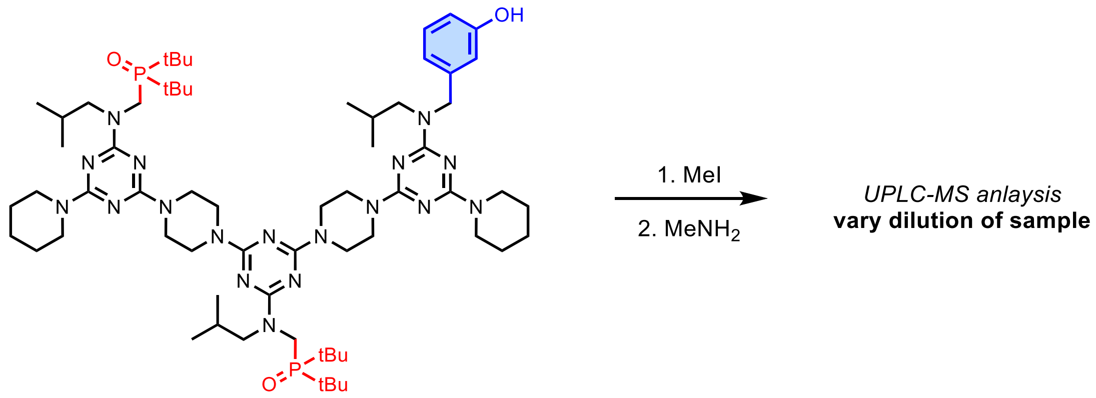

To investigate the lower limit of material needed for REMO sequencing, the 3-mer **pAADp** (4.35 mg, 3.50 µmol) was stirred in methyl iodide solution (500 µL, 80% in DMF by volume) and stirred in a sealed vial for three days. The excess methyl iodide and DMF were removed by flushing under a stream of nitrogen, redissolving in CH_2Cl_2 then again flushing with nitrogen. then the residues were redissolved in methylamine solution (500 µL, 33 wt.% in EtOH) and the solution was stirred overnight. The excess methylamine and EtOH were removed by flushing under a stream of nitrogen. The residues were dissolved in CH_2Cl_2 and 20% of the volume was removed (0.70 µmol) and dried under a stream of nitrogen. The residues were redissolved in MeOH (1.0 mL), H_2O (0.3 mL) and THF (0.1 mL). This solution (0.5 mM REMO) was further diluted 10-fold with THF (50 µM REMO). This solution was analysed by UPLC-MS (1 µL injection = 50 pmol of REMO). The solution was further dilution 10-fold with THF (5 µM REMO) and analysed twice by UPLC-MS (2 µL injection = 10 pmol of REMO, 1 µL injection = 5 pmol). The solution was further dilution 10-fold with THF (0.5 µM REMO) and analysed by UPLC-MS (2 µL injection = 1 pmol of REMO).

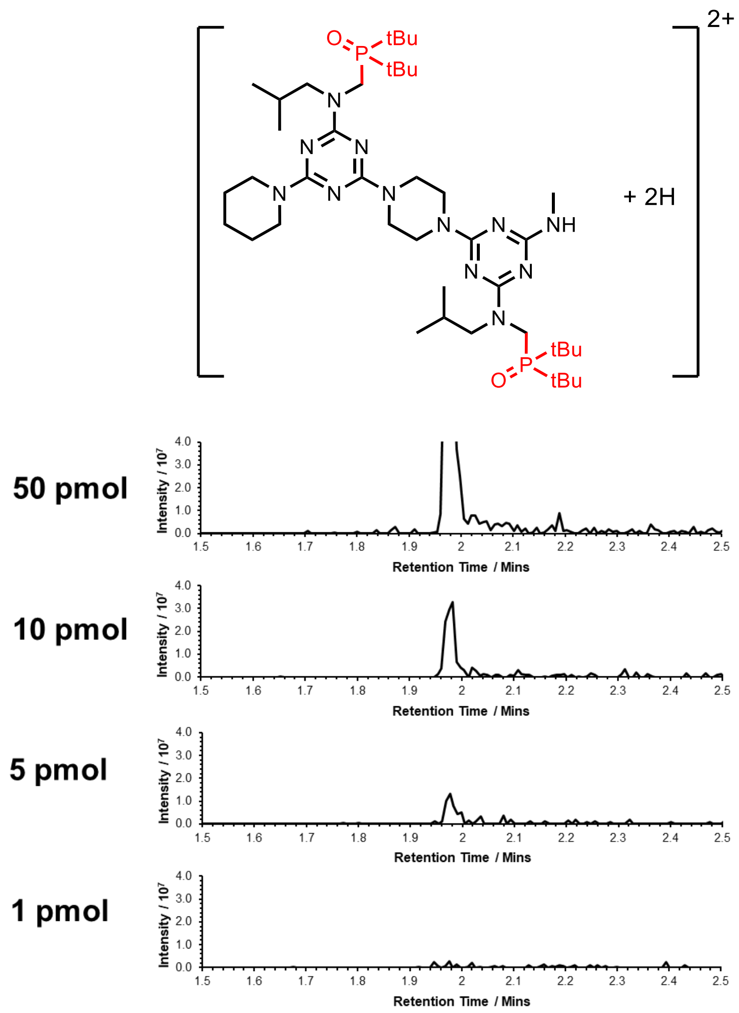

Figure S28. Determination of the detection limit for REMO sequencing. Top: structure of the diagnostic ion used to determine the sequence of **pAADp**. Bottom: extracted-ion chromatograms for the diagnostic ion (m/z ± 0.2) in the UPLC-MS analyses of different quantities of the fragment mixture of **pAADp**.

Figure S28 shows the structure of one of the **pAA** fragments that is observed in the fragment mixture of REMO **pAADp**. Note that the observation of the **pAA** fragment is sufficient information to determine the sequence of this REMO, as the only structural isomer (**pADAp**) would not contain this fragment. Also shown is the extracted-ion chromatograms for this fragment ($[M+2H]^{2+}$) in the UPLC-MS analyses of the fragment mixture, which each contained a different quantity of REMO. The **pAA** fragment can be confidently detected at 5 pmol of material, but not at 1 pmol of material. The data show that REMO can be sequenced with as little as 5 pmol of material.

Behaviour of triazoles

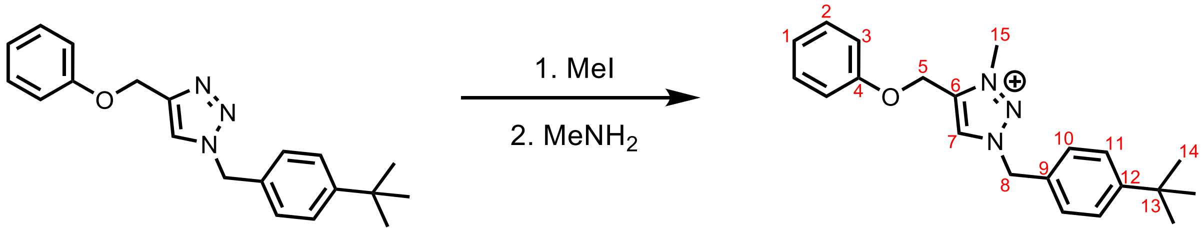

Compound **7** was stirred in methyl iodide (2 mL) and DMF (0.5 mL) for three days then the solvents were removed under a stream of nitrogen. The residue was redissolved in methylamine solution (33 wt.% in EtOH, 3 mL) and stirred overnight. The solvents were removed under a stream of nitrogen. The crude was azeotroped with acetonitrile three times and then analysed by UPLC-MS and NMR spectroscopy. The data is consistent with quantitative *N*-methylation of the triazole (structure shown above, named **[7+Me]**$^+$).

^{1}H NMR (400 MHz, CD_3CN): δ_H = 8.62 (s, 1H, H_7), 7.51 (d, *J* = 8.5 Hz, 2H, H_{11}), 7.42 (d, *J* = 8.4 Hz, 2H, H_{10}), 7.36 (dd, *J* = 8.8, 7.1 Hz, 2H, H_2), 7.11 – 7.02 (m, 3H, $H_{1,3}$), 5.74 (s, 2H, H_8), 5.33 (s, 2H, H_5), 4.26 (s, 3H, H_{15}), 1.31 (s, 9H, H_{14});

^{13}C NMR (101 MHz, CD_3CN): δ_H = 158.2 (C_4), 153.9 (C_{12}), 141.1 (C_6), 130.8 (C_2), 130.4 (C_7), 130.1 (C_9), 129.9 (C_{10}), 127.2 (C_{11}), 123.3 (C_1), 115.9 (C_3), 59.3 (C_5), 57.8 (C_8), 39.8 (C_{15}), 35.4 (C_{13}), 31.4 (C_{14});

LRMS (ESI+): calc. for $C_{21}H_{26}N_3O^+$ is 336.2, found 336.4.

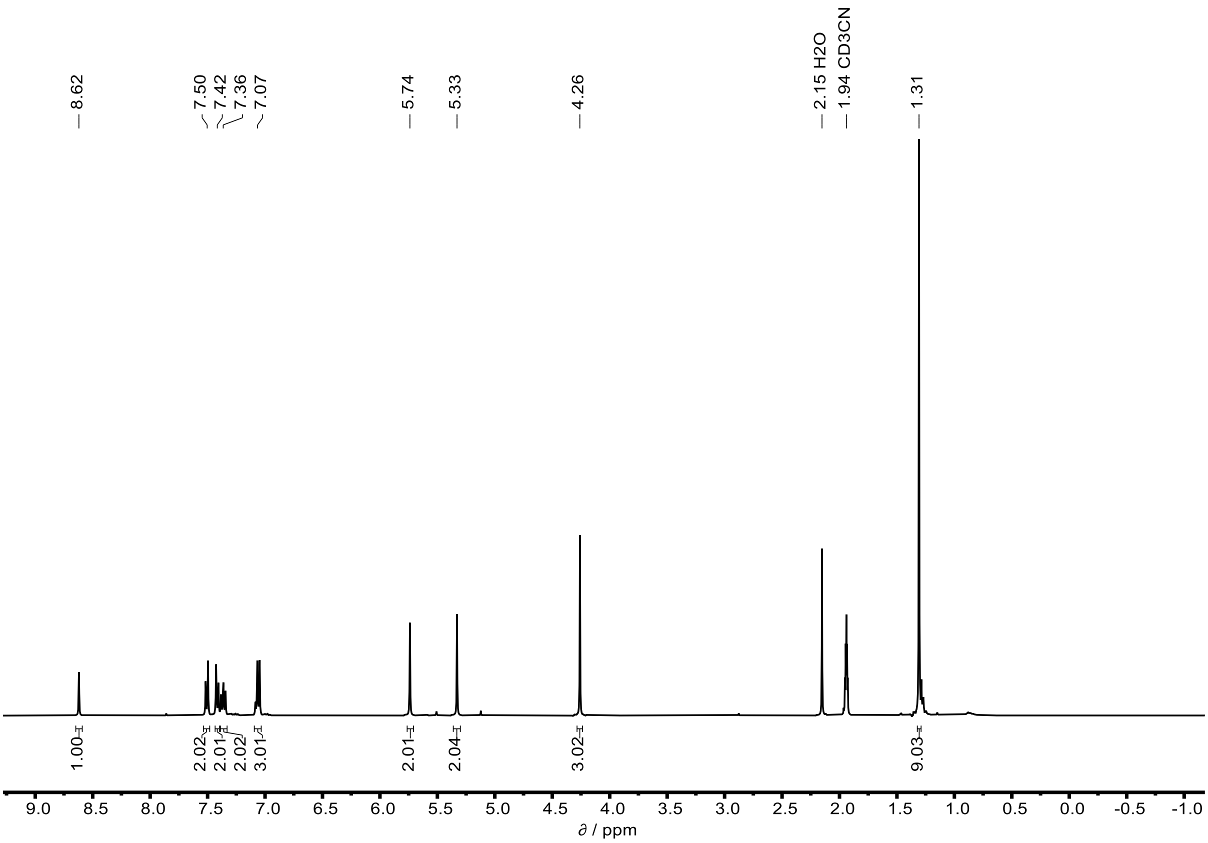

Figure S29. ^{1}H NMR spectrum (400 MHz, CD_3CN, 298 K) of crude **[7+Me]⁺**.

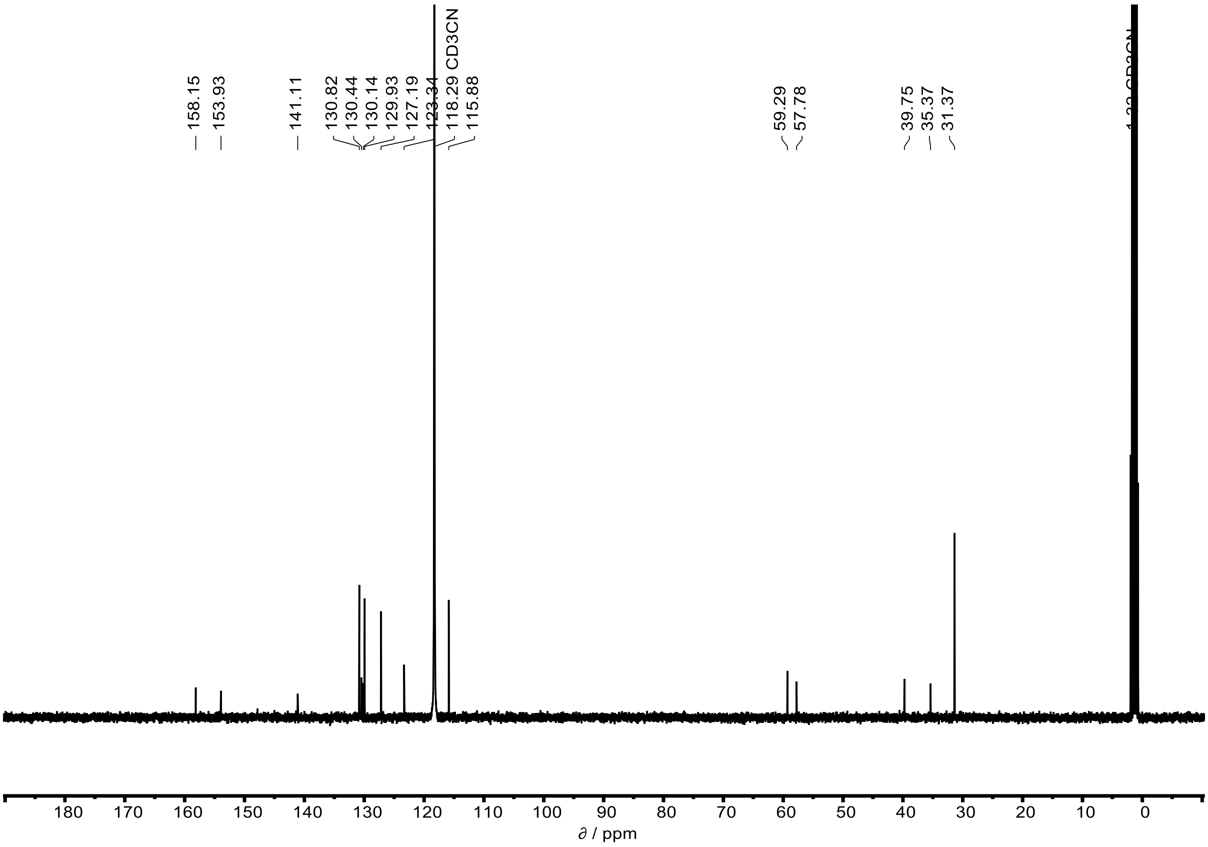

Figure S30. ^{13}C NMR spectrum (101 MHz, CD_3CN, 298 K) of crude **[7+Me]⁺**.

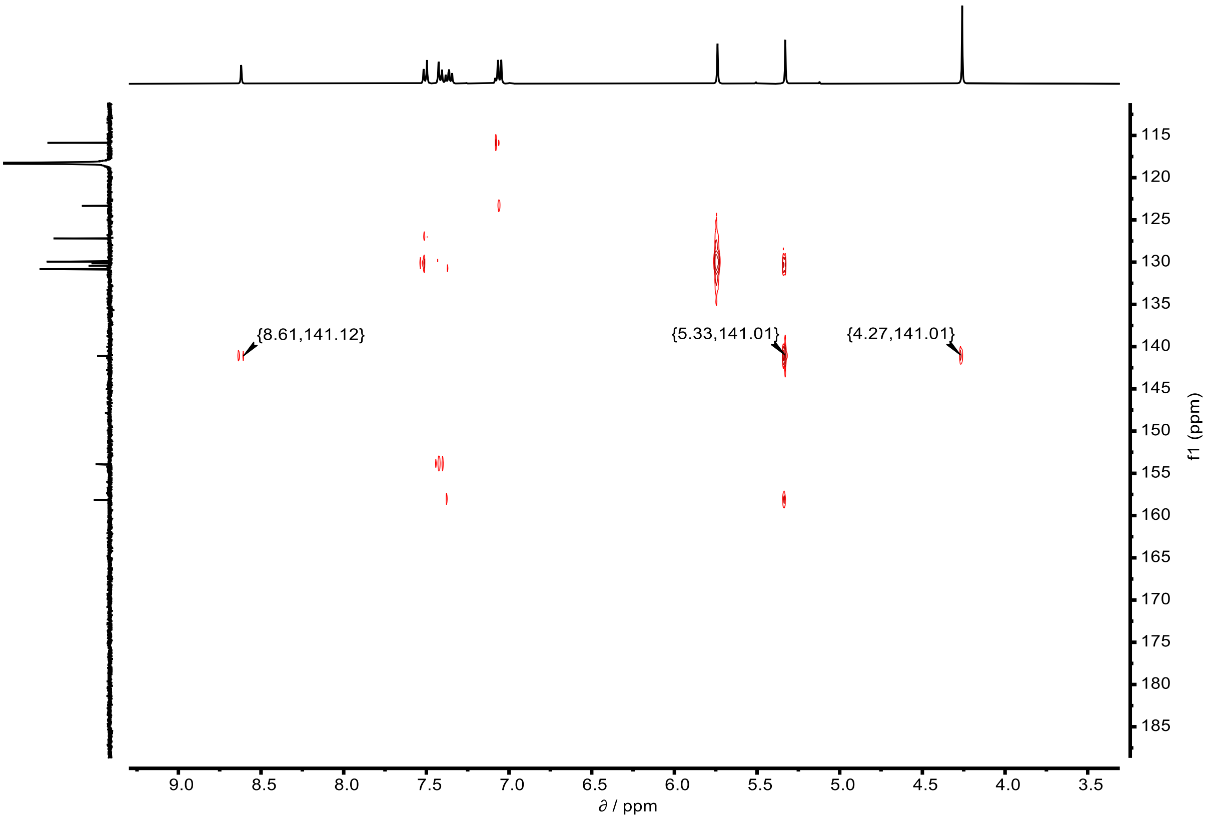

Figure S31. Partial ^{1}H-^{13}C HMBC NMR spectrum (400/101 MHz, CD_3CN, 298 K) of crude methylated compound **[7+Me]**$^+$. The highlighted correlations (H_{15}-C_6, H_5-C_6, and H_7-C_6) confirm the location of the methyl group.

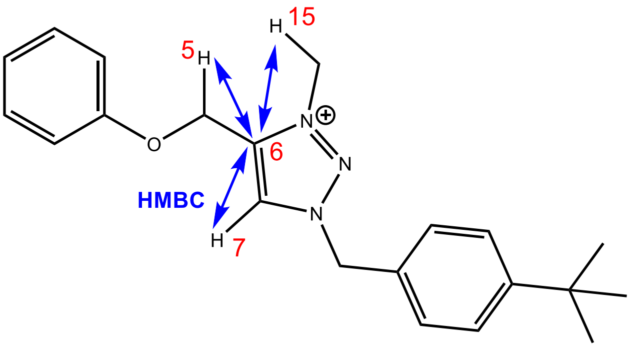

Figure S32. Key ^{1}H-^{13}C NMR correlations proving the regiochemistry of the triazole methylation in **[7+Me]**$^+$.

Tandem Mass Spectrometry

The REMO 3-mer **pAADp** was dissolved in THF + 0.1 % formic acid (approx.. 1mg/mL) and directly infused into the mass spectrometer of an Agilent HP-1200 Series LC/MSD XCT Iontrap, at a rate of 300 µL/min. The singly-charged [M+H]+ peak was isolated and subjected to collision-induced dissociation (CID) with various collision potentials using a helium collision gas.

Two new ions are observed with m/z = $[M + H]^+$ - 162 Da and m/z = $[M + H]^+$ - 324 Da fragments (Figure S33). These corresponds to the loss of one and two phosphine oxide side chains respectively. No fragments corresponding to backbone cleavage are observed.

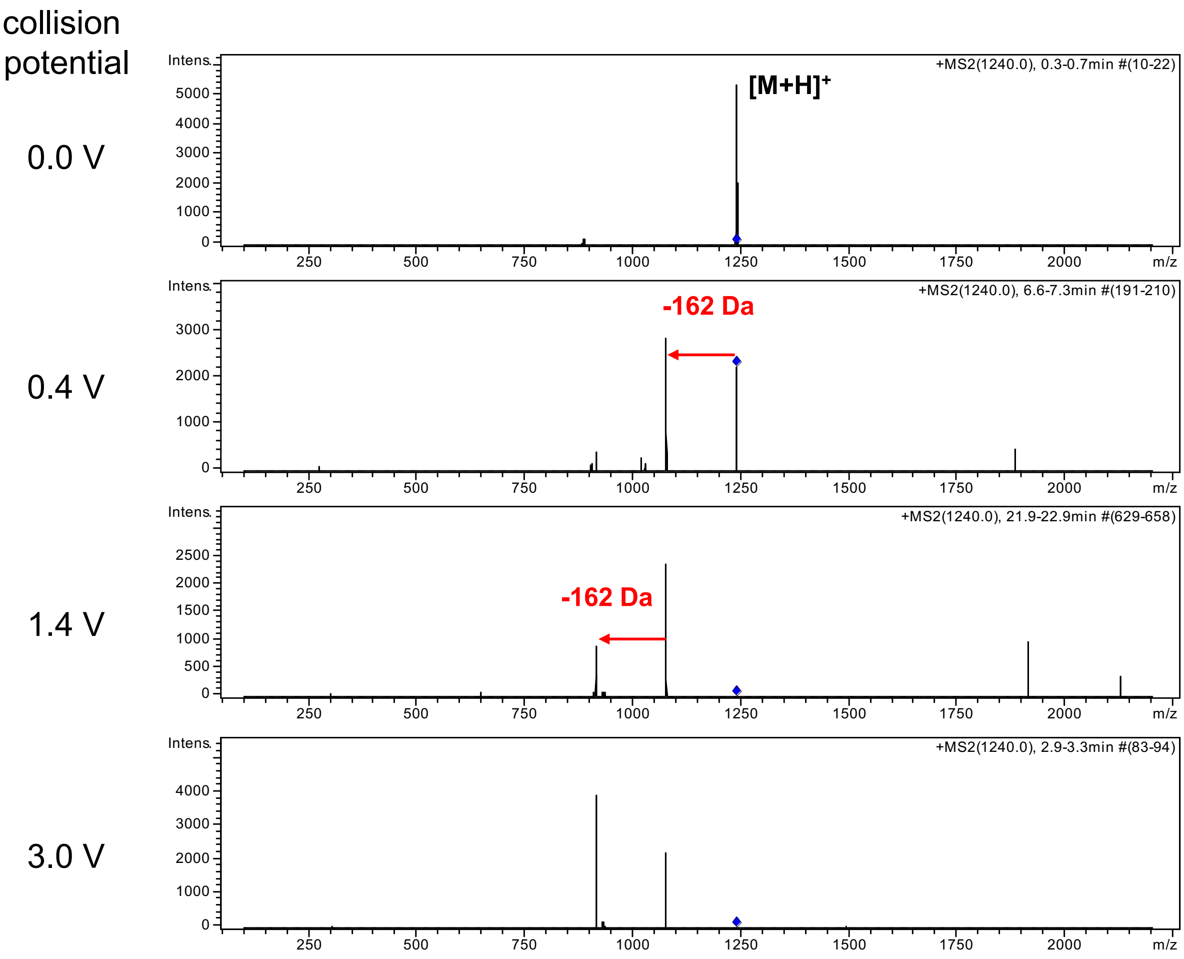

Figure S33. Tandem MS fragmentation of REMO 3-mer **pAADp** with increasing collision-induced dissociation potential.

3. Base-Filling

a. Synthesis and Characterisation

Synthesis of 10

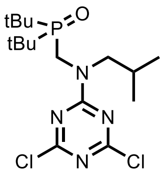

Compound **10** was synthesised according to the literature procedure.[2]

Synthesis of 11

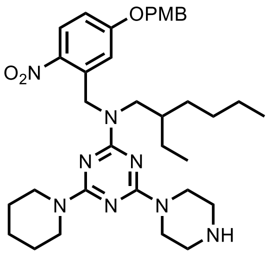

Compound **11** was synthesised according to the literature procedure.[4]

Synthesis of 12

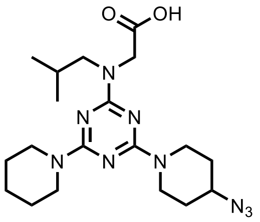

Compound **12** was synthesised according to the literature procedure.[4]

Synthesis of 13

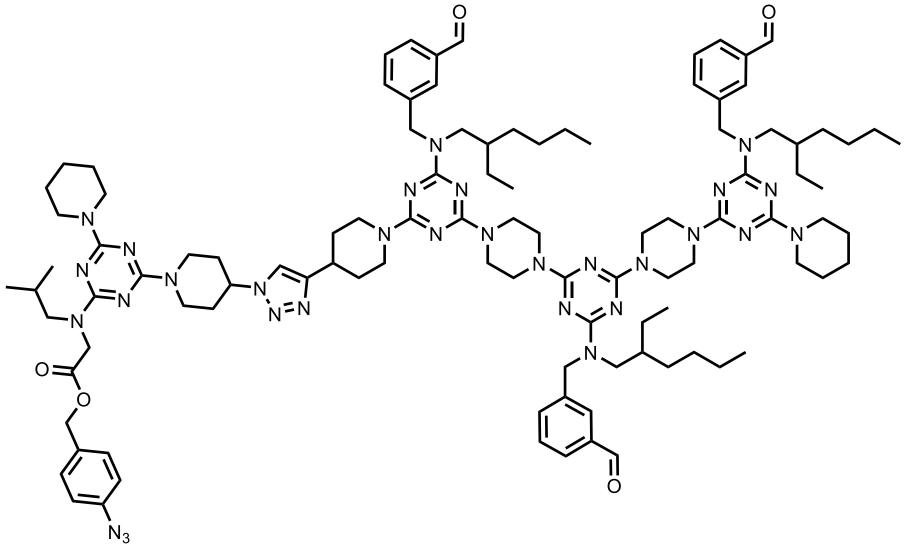

Compound **13** was synthesised according to the literature procedure.[4]

Synthesis of 14

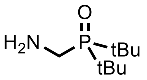

Compound **14** was synthesised according to the literature procedure.[4]

Synthesis of 15

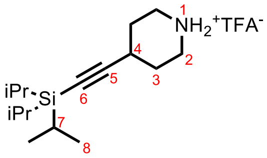

Tert-butyl 4-ethynylpiperidine-1-carboxylate (19.0 g, 91.0 mmol, 1.0 eq.) was dissolved in dry THF (180 mL) and cooled to -78 °C. Lithium bis(trimethylsilyl)amide (100 mL, 1.0 M in THF, 100 mmol, 1.1 eq.) was added dropwise to the solution under an atmosphere on nitrogen at -78 °C. The resulting solution was stirred at -78 °C for 2 hours. Trimethylsilyl chloride (30.5 mL, 99.9, 1.1 eq.) was added at -78 °C then the reaction was stirred for 15 hours at 22 °C. The solvent was removed *in vacuo* and resulting oil was dissolved in EtOAc (100 mL). The organic layer was washed with water (3 x 30 mL). The organic layer was dried with $MgSO_4$ and the solvent was removed *in vacuo*. The crude product was loaded onto a silica plug and the impurities were eluted with hexane (100 mL). The product was eluted from the plug by flushing with EtOAc (100 mL). The solvent was removed *in vacuo* and intermediate isolated as an orange oil.

The resulting intermediate was dissolved in DCM (450 mL). Trifluoroacetic acid (104 mL, 1.36 mol, 15 eq.) was added dropwise to the solution. The resulting mixture was stirred at room temperature for 2 hours then the solvent was removed *in vacuo*. Residual trifluoroacetic acid was removed by flushing the reaction mixture under N_2 and the resulting residue was recrystalised in hexane. **15** was obtained as white crystals (25.5 g, 67.1 mmol, 74 %).

^{1}H NMR (400 MHz, $CDCl_3$): δ_H = 9.37 (s, 2H, H_1), 3.32 (m, 2H, H_2), 3.15 (m, 2H, H_2), 2.91 (p, J = 4.8 Hz, 1H, H_4), 2.10 (m, 2H, H_3), 1.91 (m, 2H, H_3), 1.06 (s, 21H, $H_{7,8}$);

^{13}C NMR (101 MHz, $CDCl_3$): δ_C = 107.8 (C_5), 84.5 (C_6), 41.3 (C_2), 28.1 (C_3), 25.6 (C_4), 18.7 (C_8), 11.3 (C_7);

HRMS (ESI+): calc. for $C_{16}H_{32}NSi + H^+$ is 267.2377, found 266.2447;

FT-IR (ATR): ν_{max} /cm^{-1} 2941, 2864, 2168, 1462, 1242, 1071, 996, 919, 882, 800, 672, 598, 526.

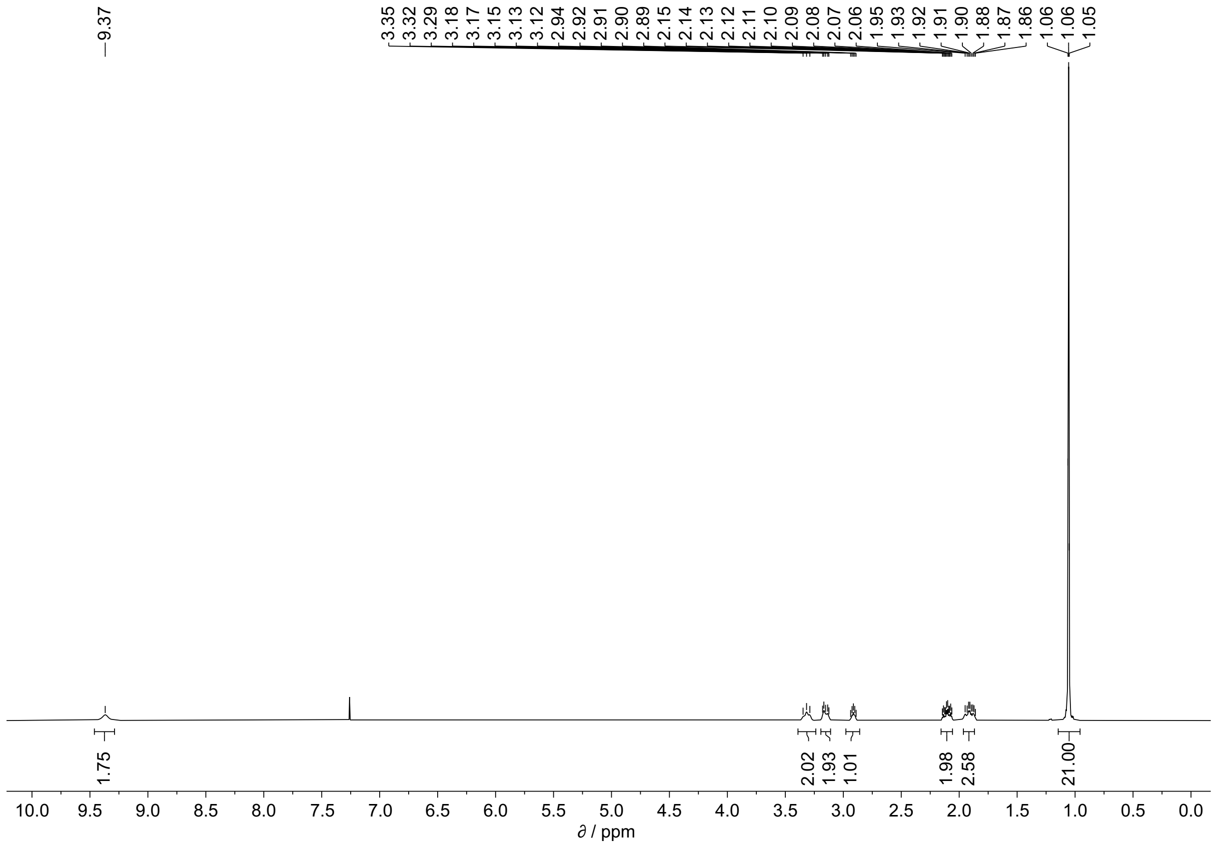

Figure S34. ^{1}H NMR spectrum (400 MHz, $CDCl_3$, 298 K) of **15**.

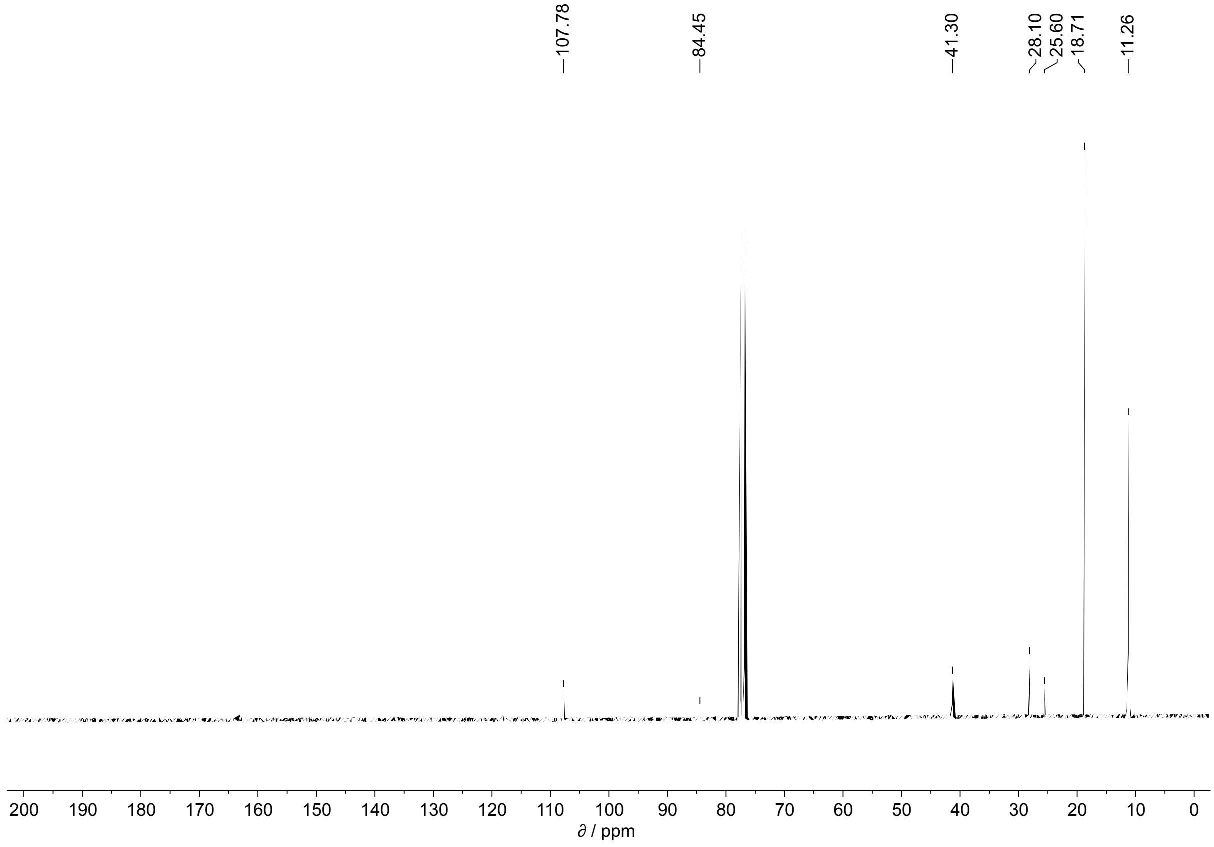

Figure S35. ^{13}C NMR spectrum (101 MHz, $CDCl_3$, 298 K) of **15**.

Synthesis of 16

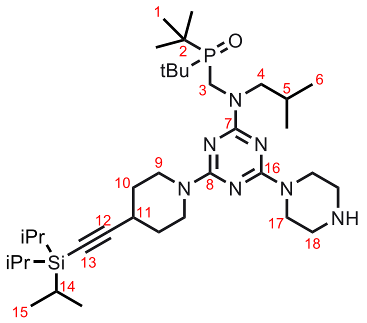

10 (0.630 g, 1.60 mmol, 1 eq.) was dissolved in THF (16 mL, 100 mM) and cooled to -78 °C. **15** (1.50 mL, 1.07 M in THF, 1.60 mmol, 1 eq.) and DIPEA (0.83 mL, 4.8 mmol, 3 eq.) were added dropwise then the temperature of the resulting mixture was raised to 15 °C and the mixture was stirred for 2 hours. A saturated solution of piperazine (5.49 mL, 2.3 M in THF, 12.8 mmol, 8 eq.) was added to the solution. The mixture was heated under microwave irradiation at 60 °C for 60 minutes. The solvent was removed *in vacuo*. The remaining residue was dissolved in EtOAc (30 mL) and washed with water (3 x 30 mL). The organic layer was dried with $MgSO_4$ and the solvent was removed *in vacuo*. The crude product was purified by flash chromatography (SiO_2, 0 – 20 % gradient of MeOH in DCM). **16** was obtained as a white foam (0.537 g, 0.797 mmol, 50%).

^{1}H NMR (400 MHz, $CDCl_3$): δ_H = 4.36 (br s, 2H, H_3), 3.93 (br, 2H, H_{17}), 3.79 (d, J = 7.3 Hz, 2H, H_4), 3.72 (t, J = 5.0 Hz, 4H, H_9), 3.65 (br, 2H, H_{17}), 2.86 (t, J = 5.0 Hz, 4H, H_{18}), 2.72 (br, 1H, H_{11}), 2.57 (br s, 1H, NH), 2.17 (hept, J = 6.9 Hz, 1H, H_5), 1.77 and 1.62 (rotamers, br, 4H ,H_{10}), 1.25 (d, $^3J_{HP}$ = 12.9 Hz, 18H, H_1), 1.09 – 0.92 (m, 21H, $H_{14,15}$), 0.85 (d, J = 6.7 Hz, 6H, H_6);

^{13}C NMR (101 MHz, $CDCl_3$): δ_C =165.6 (C_7), [165.3, 164.8 ($C_{8,16}$)], 111.2 (C_{12}), 81.7 (C_{13}), 53.1 (C_4), 46.0 (C_{18}), 44.2 (C_{17}), 41.3 (C_9), 38.1 (d, $^1J_{CP}$ = 59.8 Hz, C_3), 35.8 (d, $^1J_{CP}$ = 55.5 Hz, C_2), 31.6 (C_{10}), 26.9 (C_1), 26.5 (C_5), 20.6 (C_6), 18.8 (C_{15}), 11.3 (C_{14});

^{31}P NMR (162 MHz, $CDCl_3$): δ_P = 59.0, 58.9;

HRMS (ESI+): Calculated for $C_{36}H_{69}N_7OPSi^+$, 674.5065; found 674.5188;

FT-IR (ATR): ν_{max} /cm^{-1} 2945, 2865, 2212, 2167, 1529, 1482, 1432, 1368, 1314, 1240, 1207, 1151, 1074, 996, 907, 883, 834, 807, 726, 673, 643.

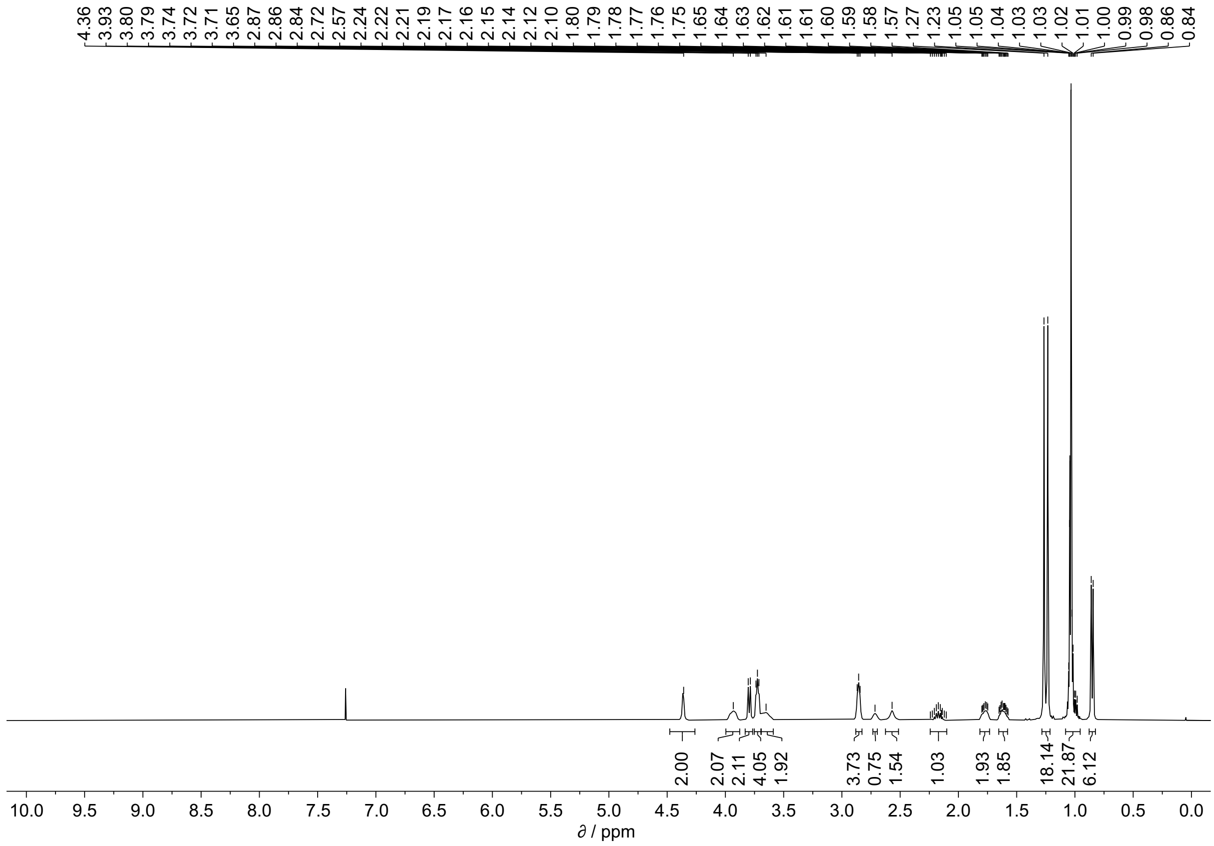

Figure S36. ^{1}H NMR spectrum (400 MHz, $CDCl_3$, 298 K) of **16**.

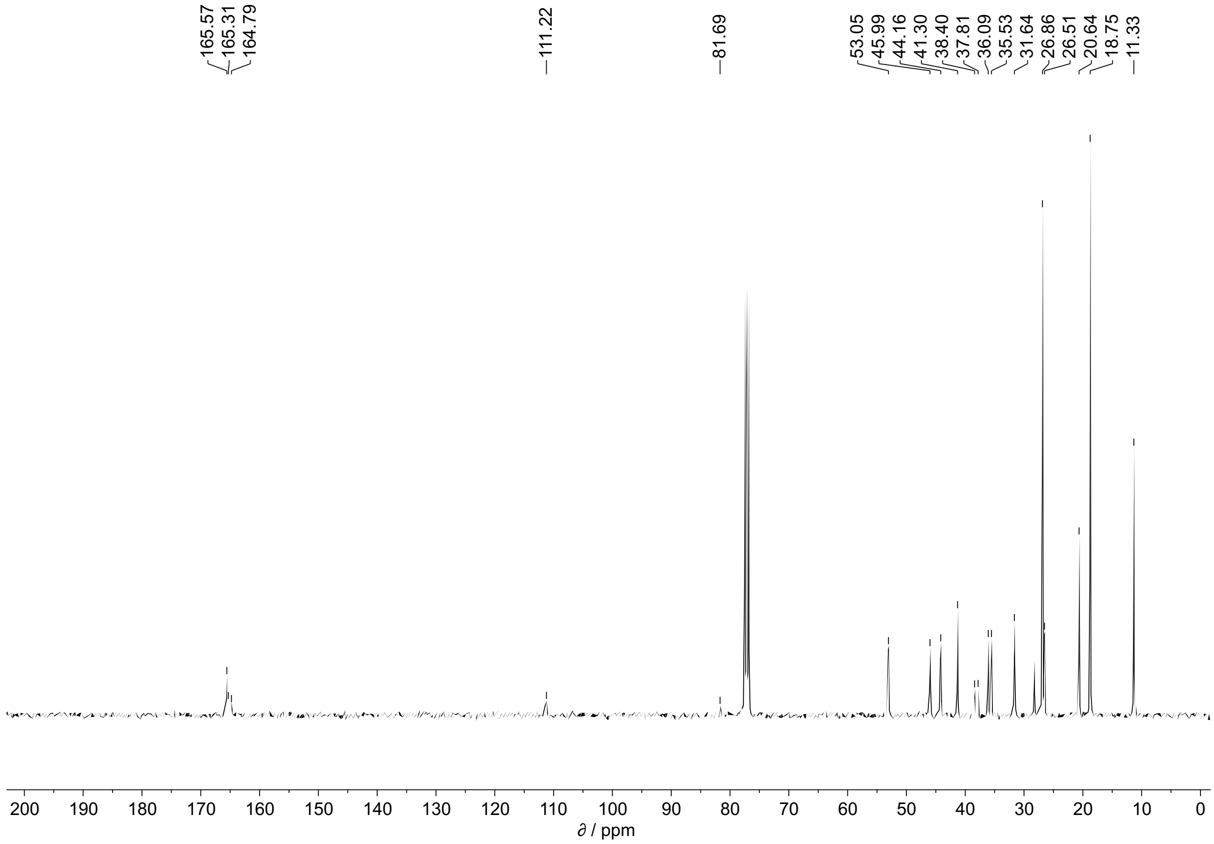

Figure S37. ^{13}C NMR spectrum (101 MHz, $CDCl_3$, 298 K) of **16**.

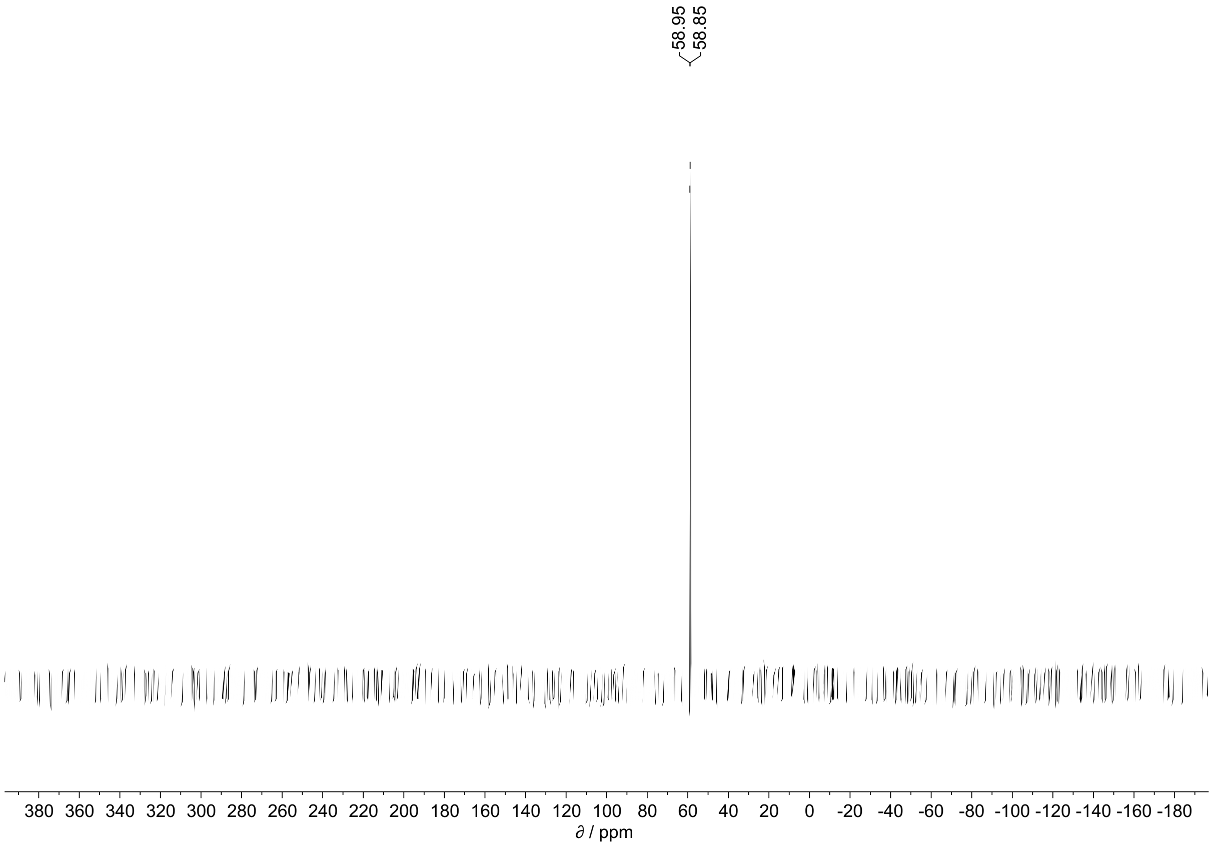

Figure S38. ^{31}P NMR spectrum (162 MHz, $CDCl_3$, 298 K) of **16**.

Synthesis of 17

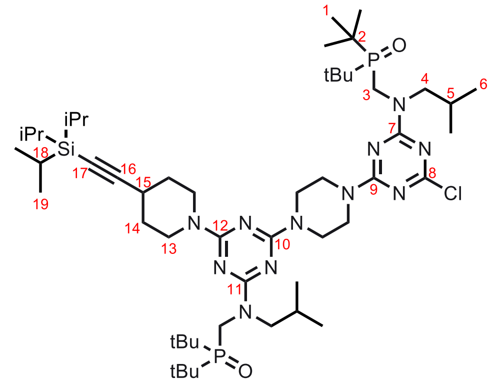

10 (0.800 g, 2.03 mmol, 1 eq.) was dissolved in THF (7 mL) and cooled to -78 °C. A solution of **16** (1.27 g, 2.03 mmol, 1 eq.) and DIPEA (1.06 mL, 6.09 mmol, 3 eq) in THF (7 mL) was added dropwise to the solution of **10** at -78 °C. The solution was stirred at room temperature for 16 hours. The solvent was removed *in vacuo.* The remaining residue was dissolved in EtOAc (30 mL) and washed with water (3 × 30 mL). The organic layer was dried with $MgSO_4$ and the solvent was removed *in vacuo*. The crude product was purified by flash chromatography (SiO_2, 0 – 6 % gradient of MeOH in DCM). **17** was obtained as a white foam (1.27 g, 1.23 mmol, 61 %).

^{1}H NMR (700 MHz, $CDCl_3$): δ_H =[4.38, 4.32 (both br s, 2H, H_3)], 3.95 (br , 2H, H_{13}), [3.85, 3.83 (both d, J = 7.5 Hz, H_4)], 3.79 (br, 8H, protons of piperazine ring), 3.69 (br, 2H, H_{13}), 2.75 (br, 1H, H_{15}), 2.19 (m, 2H, H_5), 1.80 and 1.64 (rotamers, br, 4H, H_{14}), [1.28, 1.26 (both d, $^{3}J_{HP}$ = 13.0, 18H, H_1)], [1.06, 1.05 (s, 21H, $H_{18,19}$)], [0.90, 0.87 (both d, J = 6.9 Hz, 6H, H_6)];

^{13}C NMR (176 MHz, $CDCl_3$): δ_C = [169.7, 169.2 (rotamers, C_8), [165.6, 165.4, 165.4, 165.2, 165.1, 164.9, 164.7, 164.5, 164.3 ($C_{7,9,10,11,12}$)], [111.3, 111.1 (rotamers, C_{16})], [81.8, 81.6 (rotamers, C_{17})], [53.8, 53.4, 53.1 (C_4)], [43.5, 43.1, 42.8 (carbons of piperazine ring)], [41.4, 41.4 (rotamers, C_{13})], [38.4, 38.2 (both d, $^{1}J_{CP}$ = 56.2 Hz, C_3)], [36.0, 35.9 (both d, $^{1}J_{CP}$ = 55.8 Hz, C_2)], [31.7, 31.7 (rotamers, C_{14})], 28.2 (C_{15}), [26.9, 26.9, 26.8 (C_1)], [26.4, 26.4 (C_5)], [20.7, 20.6, 20.2 (C_6)], 18.8 (C_{19}), 11.4 (C_{18});

^{31}P NMR (203 MHz, $CDCl_3$): δ_P = 58.8, 58.7;

HRMS (ESI+): Calculated for $C_{52}H_{97}ClN_{11}O_2P_2Si^+$, 1032.6754; found 1032.6936;

FT-IR (ATR): ν_{max} /cm^{-1} 2953, 2866, 1566, 1532, 1486, 1434, 1368, 1314, 1233, 1159, 988, 832, 808, 654, 503.

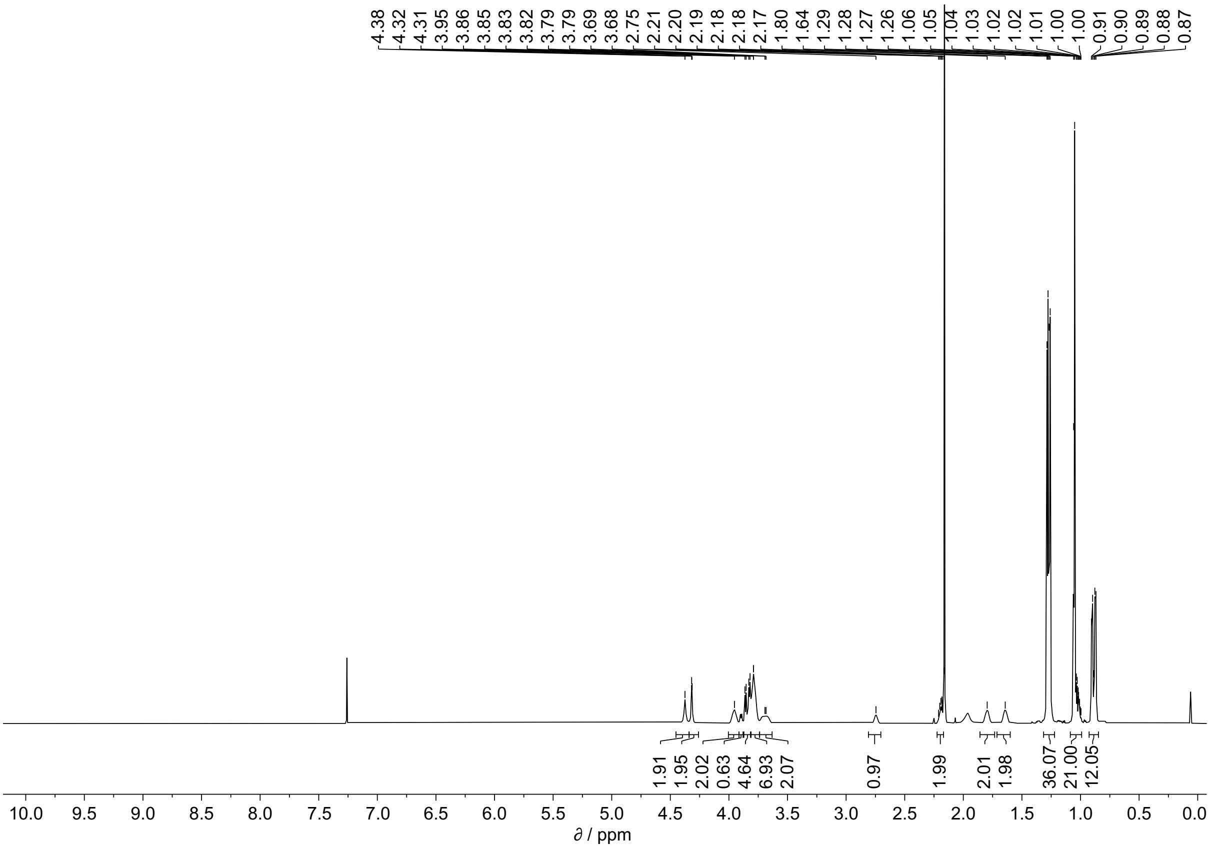

Figure S39. ^{1}H NMR spectrum (700 MHz, $CDCl_3$, 298 K) of **17**.

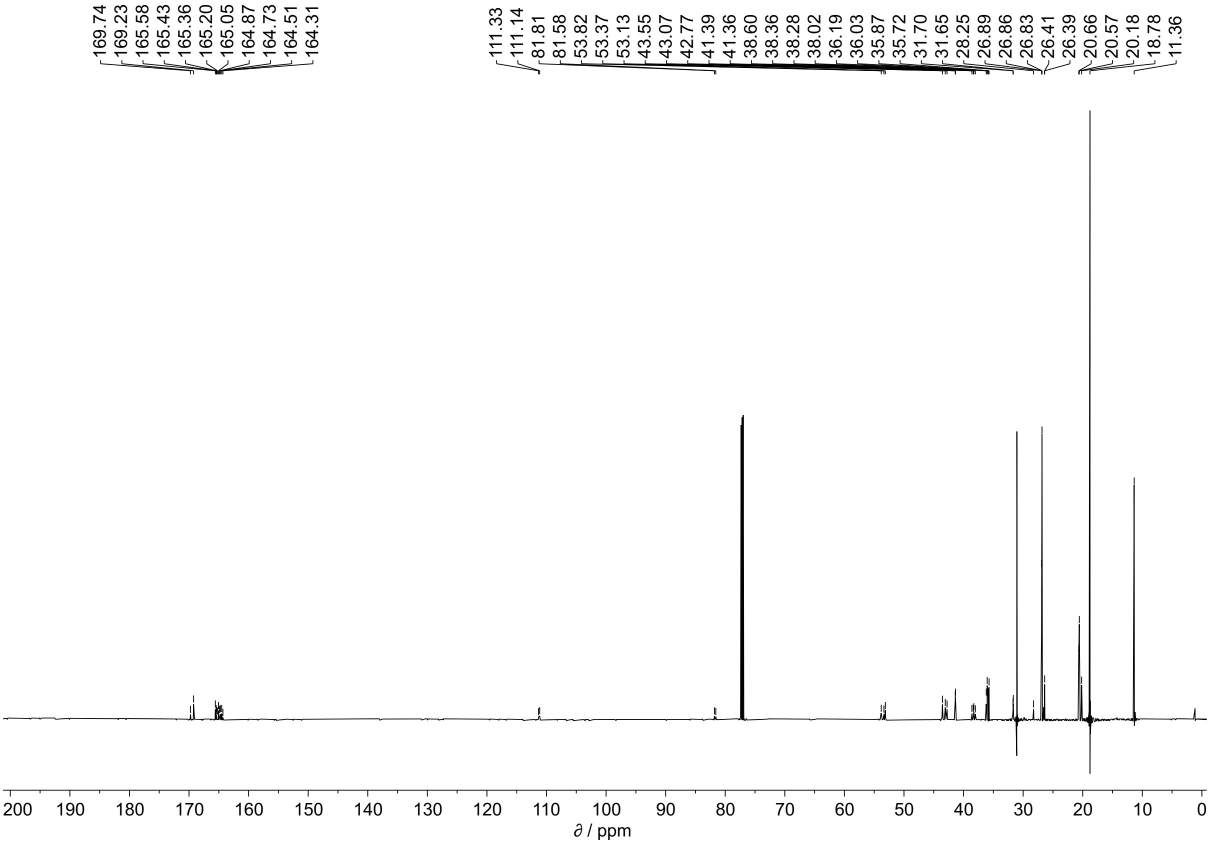

Figure S40. ^{13}C NMR spectrum (176 MHz, $CDCl_3$, 298 K) of **17**.

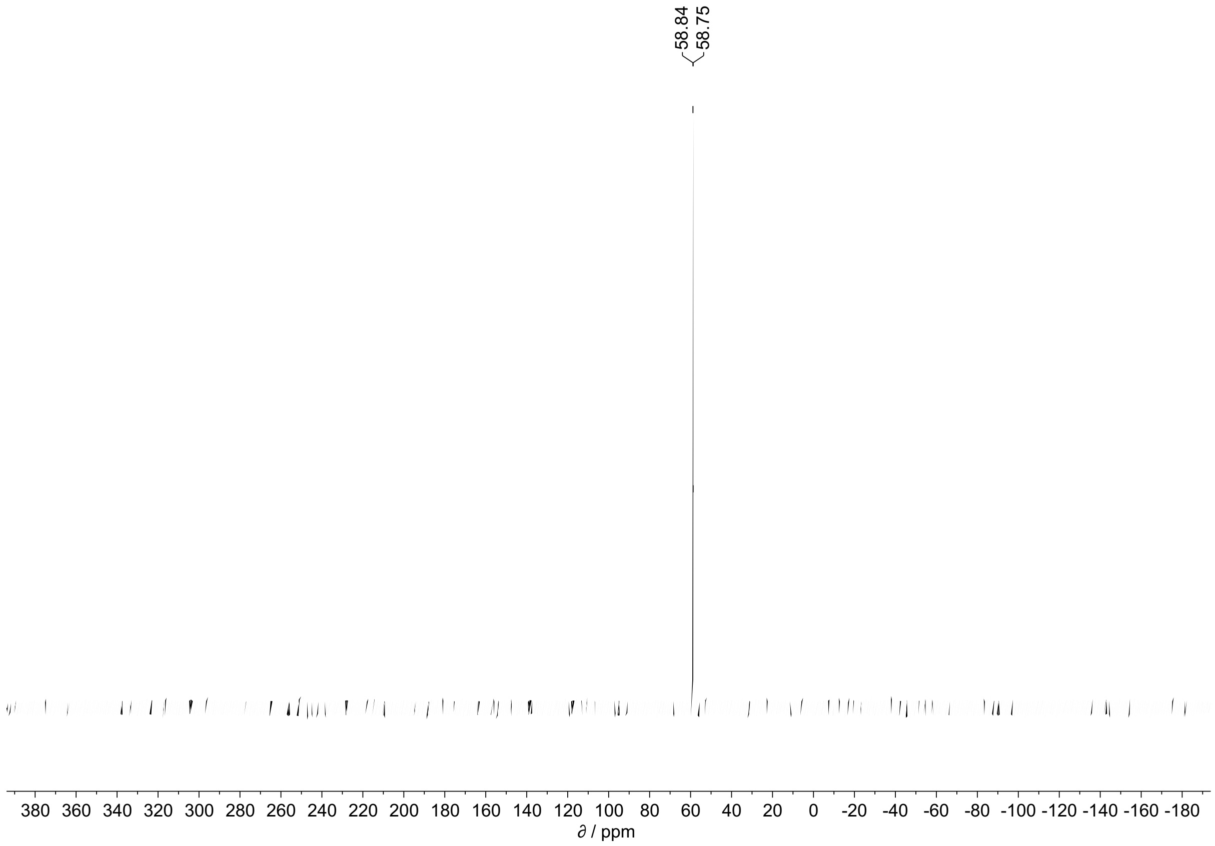

Figure S41. ^{31}P NMR spectrum (203 MHz, $CDCl_3$, 298 K) of **17**.

Synthesis of 18

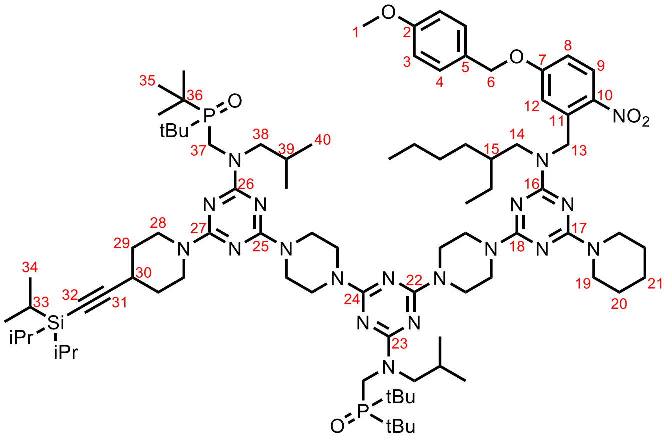

To a solution of **17** (40 mg, 0.039 mmol, 1.0 eq.) in THF (0.5 mL), **11** (125 mg, 0.19 mmol, 5.0 eq.) and DIPEA (34 µL, 0.19 mmol, 5.0 eq.) were added. The solution was heated at 65 °C under microwave irradiation for 30 minutes then cooled to room temperature. The solvent was removed under reduced pressure and the obtained residue was redissolved in ethyl acetate (10 mL). The organic layer was washed with water (10 mL), dried with magnesium sulphate and the solvent removed under reduced pressure. The obtained residue was purified by silica flash chromatography (DCM/methanol (95:5)). **18** was obtained as a yellow oil (54 mg, 0.033 mmol, 85%).

^{1}H NMR (700 MHz, CDCl$_3$): δ_H = 8.10 (br, 1H, H_9), 7.27 (br, 2H, H_4), 6.86 (m, 3H, $H_{3,8}$), 6.78 and 6.76 (rotamers, s, 1H, H_{12}), 5.10 (br, 2H, H_{13}), 4.97 (s, 2H, H_6), 4.39 (br, 4H, H_{37}), 3.97 (br, 2H, H_{28}), 3.90 – 3.30 (br, 31H, $H_{1,14,19,28,38}$ and protons of piperazine rings), 2.75 (br, 1H, H_{30}), 2.20 (br, 2H, H_{39}), 1.80 (br, 3H, $H_{15,29}$), 1.65 (br, 4H, $H_{20,21,29}$), 1.57 (br, 4H, $H_{20,21}$), 1.32 – 1.17 (br, 44H, H_{35} and CH_2 protons of alkyl chain), 1.05 (s, 21H, $H_{33,34}$), 0.89 (br, 18H, H_{40} and methyl protons of alkyl chain);

^{13}C NMR (176 MHz, CDCl$_3$): δ_C = 166.2 ($C_{16,23,26}$), [165.6, 165.5, 165.3, 165.2, 165.2, 165.0, 164.9, 164.8 ($C_{17,18,22,24,25,27}$)], [163.0, 162.9 (rotamers, C_7)], 159.8 (C_2), 141.5 (C_{10}), 139.6 (C_{11}), 129.6 (C_4), 127.8 (C_9), 127.7 (C_5), 114.2 ($C_{3,12}$), [112.9, 112.8 (rotamers, C_8)], [111.4, 111.2 (rotamers, C_{31})], [81.8, 81.5 (rotamers, C_{32})], 70.4 (C_6), 55.4 (C_1), 53.1 (C_{38}), 51.1 (C_{14}), 49.0 (C_{13}), [44.3, 44.1, 43.3, 43.2, 43.1, 43.0 ($C_{19,28}$ and carbons of piperazine rings)], 41.3 (C_{28}), 38.3 (C_{15}), 38.0 (C_{37}), 35.9 (d, $^1J_{CP}$ = 56.1 Hz, C_{36}), 31.6 (C_{29}), [30.8, 29.8, 28.9 (CH_2 carbons of alkyl chain)], 28.3 (C_{30}), 26.9 (C_{35}), 26.6 (C_{39}), [26.0, 25.8, 25.2, 25.0 ($C_{20,21}$)], [24.0, 23.3 (CH_2 carbons of alkyl chains)], 20.7 (C_{40}), 18.8 (C_{34}), [14.3, 14.2 (methyl carbons of alkyl chain)], 11.4 (C_{33}), [10.9, 10.8 (methyl carbons of alkyl chain)];

^{31}P NMR (162 MHz, CDCl$_3$): δ_P = 58.8, 58.8, 58.7;

HRMS (ESI+): Calculated for $C_{87}H_{146}N_{19}O_6P_2Si^+$, 1643.0942; found 1643.0953;

FT-IR (ATR): ν_{max} /cm^{-1} 2953, 2926, 2863, 1612, 1525, 1477, 1430, 1367, 1350, 1314, 1286, 1253, 1174, 1145, 1074, 1027, 997, 884, 833, 807, 734, 674, 652, 587, 505, 450.

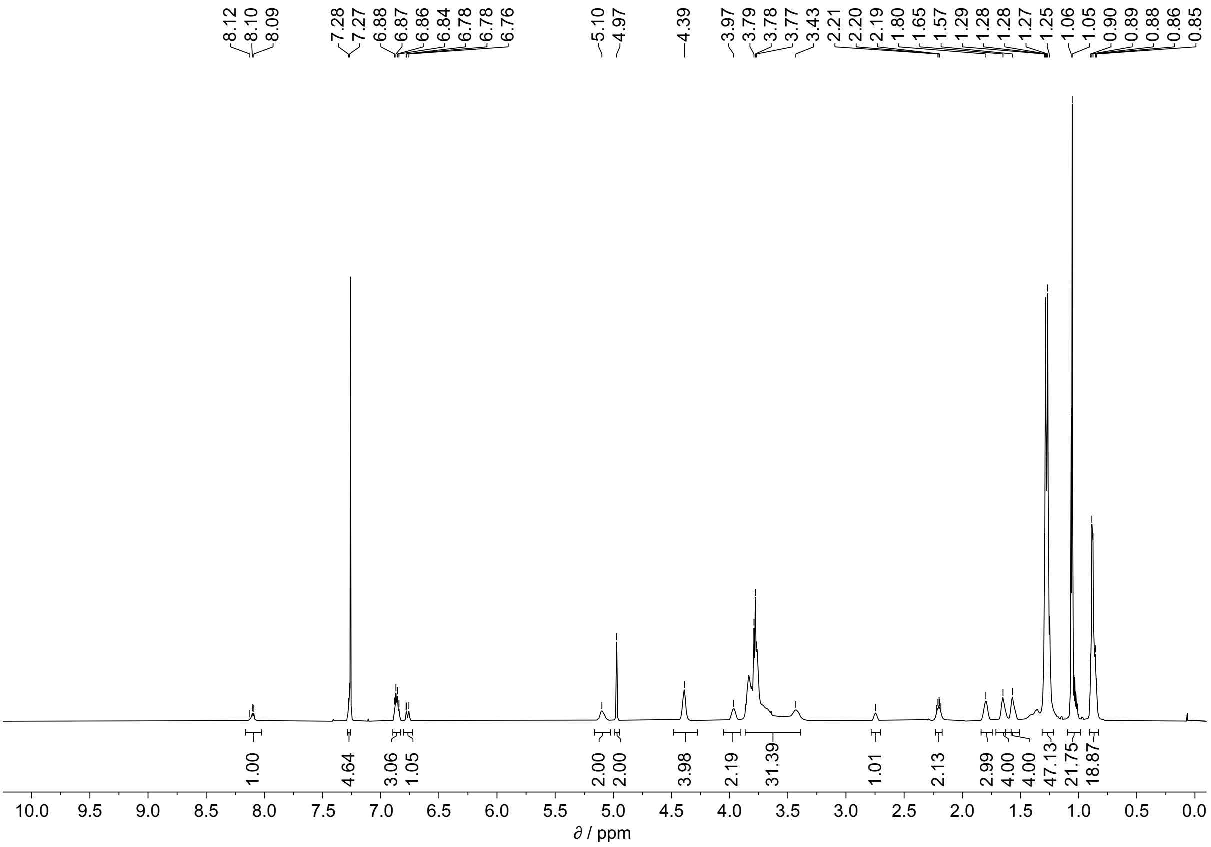

Figure S42. ^{1}H NMR spectrum (700 MHz, CDCl$_3$, 298 K) of **18**.

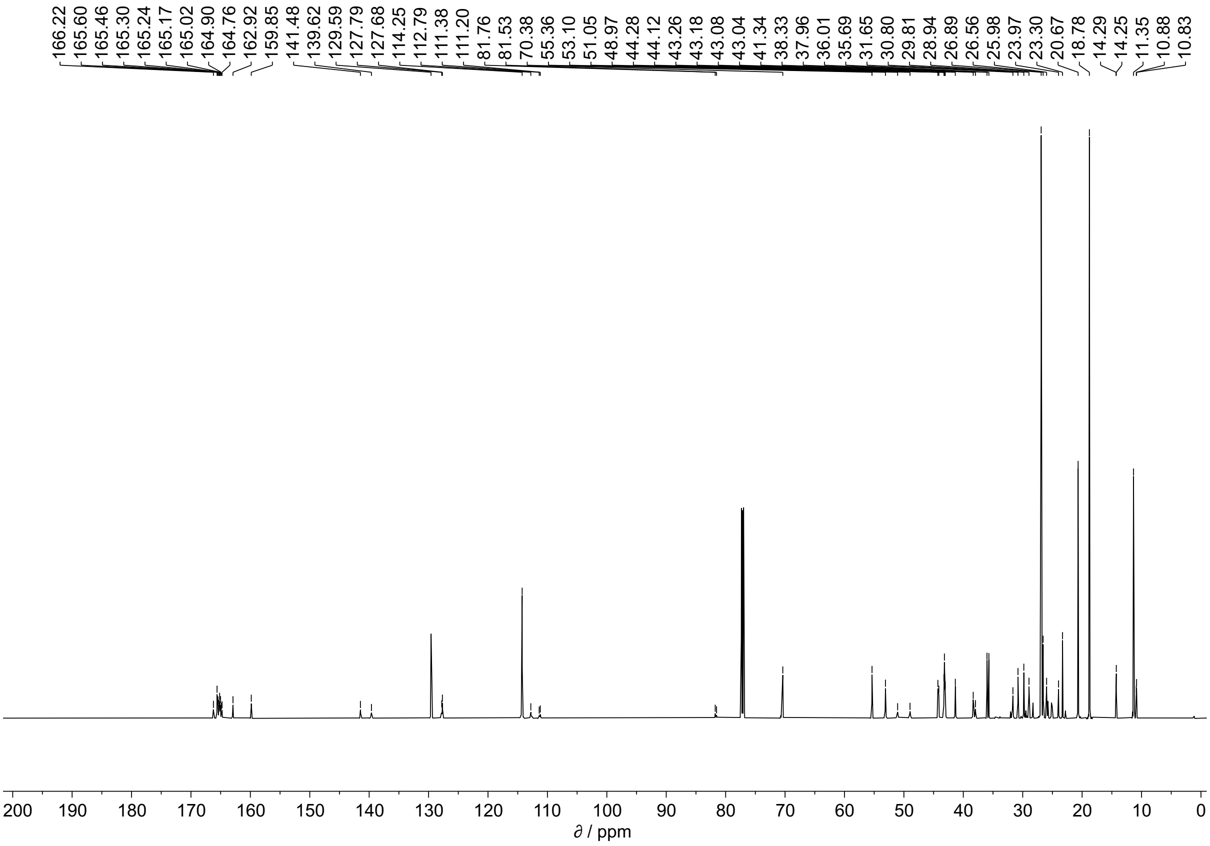

Figure S43. ^{13}C NMR spectrum (176 MHz, $CDCl_3$, 298 K) of **18**.

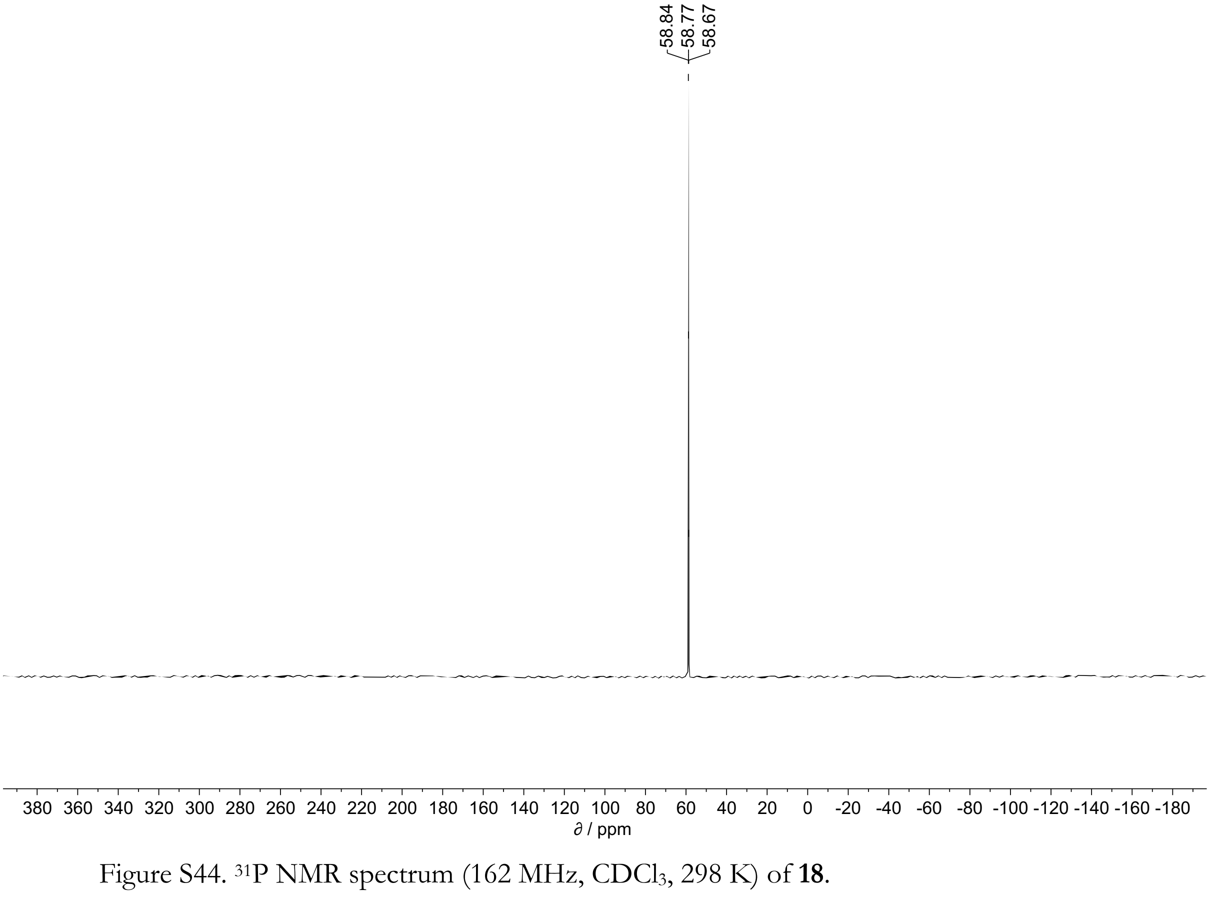

Figure S44. ^{31}P NMR spectrum (162 MHz, $CDCl_3$, 298 K) of **18**.

Synthesis of 19

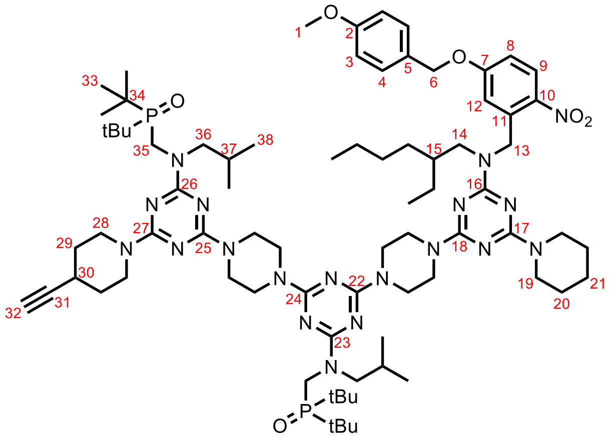

To a solution of **18** (54 mg, 0.033 mmol) in THF (1 mL), a solution of TBAF in THF (0.1 mL, 0.1 mmol) was added and the reaction stirred at room temperature for 30 minutes. The solution was diluted with ethyl acetate (10 mL) and washed with saturated ammonium chloride (2 x 10 mL). The organic phase was dried over magnesium carbonate and the solvent removed under reduced pressure. **19** was obtained as a yellow oil and used without further purification (48 mg, 0.032 mmol, 97%).

^{1}H NMR (700 MHz, CDCl$_3$): δ_H = 8.10 (br, 1H, H$_9$), 7.27 (br, 2H, H$_4$), 6.86 (m, 3H, H$_{3,8}$), 6.78 and 6.76 (rotamers, s, 1H, H$_{12}$), 5.09 (br, 2H, H$_{13}$), 4.97 (s, 2H, H$_6$), 4.39 (br, 4H, H$_{35}$), 4.16 (br, 2H, H$_{28}$), 3.90 – 3.31 (br, 31H, H$_{1,14,19,28,36}$ and protons of piperazine rings), 2.65 (br, 1H, H$_{30}$), 2.20 (br, 2H, H$_{37}$), 2.11 (s, 1H, H$_{32}$), 1.82 (br, 2H, H$_{29}$), 1.79 (br, 1H, H$_{15}$), 1.65 (br, 2H, H$_{20,21}$), 1.62 (br, 2H, H$_{29}$), 1.57 (br, 4H, H$_{20,21}$), 1.25 (br, 44H, H$_{33}$ and CH$_2$ protons of alkyl chain), 0.88 (br, 18H, H$_{38}$ and methyl protons of alkyl chain);

^{13}C NMR (176 MHz, CDCl$_3$): δ_C = 166.2 (C$_{16,23,26}$), [165.6, 165.6, 165.4, 165.3, 165.2, 165.2, 165.0, 165.0, 164.8 (C$_{17,18,22,24,25,27}$)], [163.0, 162.9 (rotamers, C$_7$)], 159.8 (C$_2$), [141.5, 141.4 (rotamers, C$_{10}$)], 139.6 (C$_{11}$), 129.6 (C$_4$), 127.8 (C$_9$), 127.7 (C$_5$), 114.2 (C$_{3,12}$), [112.9, 112.8, 112.6 (rotamers, C$_8$)], [87.0, 86.9 (rotamers, C$_{31}$)], 70.4 (C$_6$), [69.5, 69.4 (rotamers, C$_{32}$)], 55.4 (C$_1$), 53.1 (C$_{36}$), 51.1 (C$_{14}$), 49.0 (C$_{13}$), [44.3, 44.1, 43.2, 43.2, 43.1, 43.0 (C$_{19,28}$ and carbons of piperazine rings)], 41.7 (C$_{28}$), 38.3 (C$_{15}$), 38.0 (C$_{35}$), 35.9 (d, $^1J_{CP}$ = 55.2 Hz, C$_{34}$), 31.4 (C$_{29}$), [30.8, 29.8, 28.9 (CH$_2$ carbons of alkyl chain)], 27.4 (C$_{30}$), 26.9 (C$_{33}$), 26.5 (C$_{37}$), [26.0, 25.8, 25.2, 25.0 (C$_{20,21}$)], [24.0, 23.3 (CH$_2$ carbons of alkyl chain)], 20.7 (C$_{38}$), [14.3, 14.2, 10.9, 10.8 (methyl carbons of alkyl chain)];

^{31}P NMR (162 MHz, CDCl$_3$): δ_P = 59.0, 58.8;

HRMS (ESI+): Calculated for $C_{78}H_{126}N_{19}O_6P_2^+$, 1486.9608; found 1486.9607;

FT-IR (ATR): ν_{max} /cm^{-1} 3289, 2953, 2926, 2858, 1612, 1578, 1525, 1476, 1427, 1387, 1367, 1349, 1314, 1285, 1250, 1175, 1143, 1074, 1024, 996, 932, 909, 885, 833, 806, 730, 672, 643, 580, 504, 448.

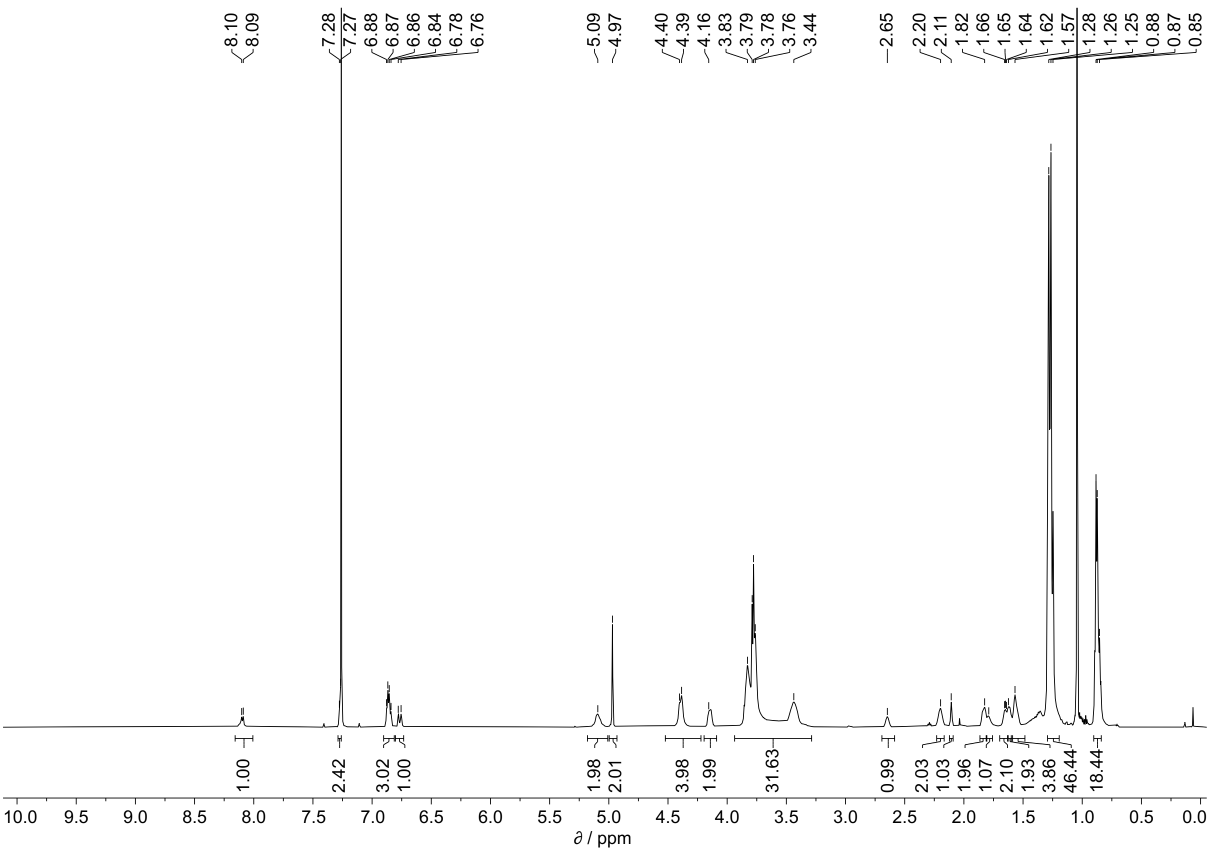

Figure S45. ^{1}H NMR spectrum (700 MHz, $CDCl_3$, 298 K) of **19**.

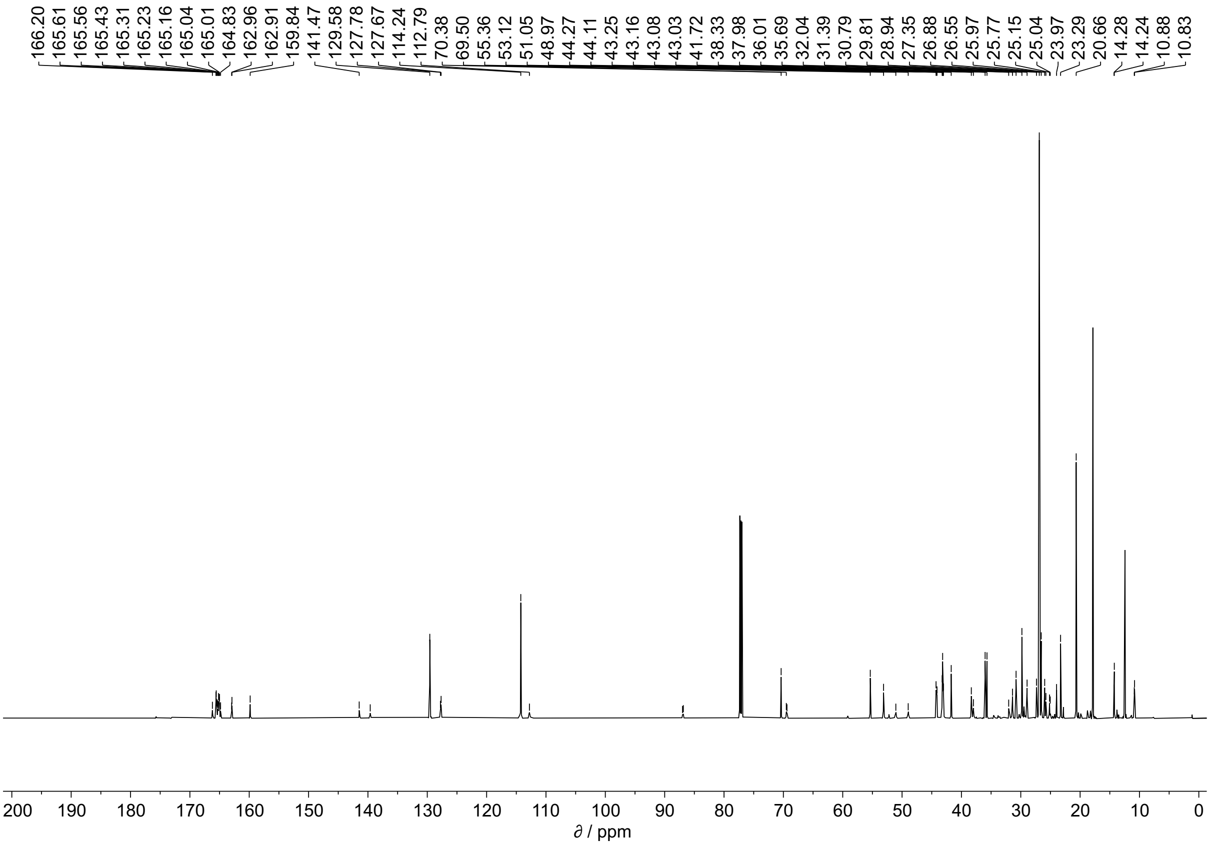

Figure S46. ^{13}C NMR spectrum (176 MHz, $CDCl_3$, 298 K) of **19**.

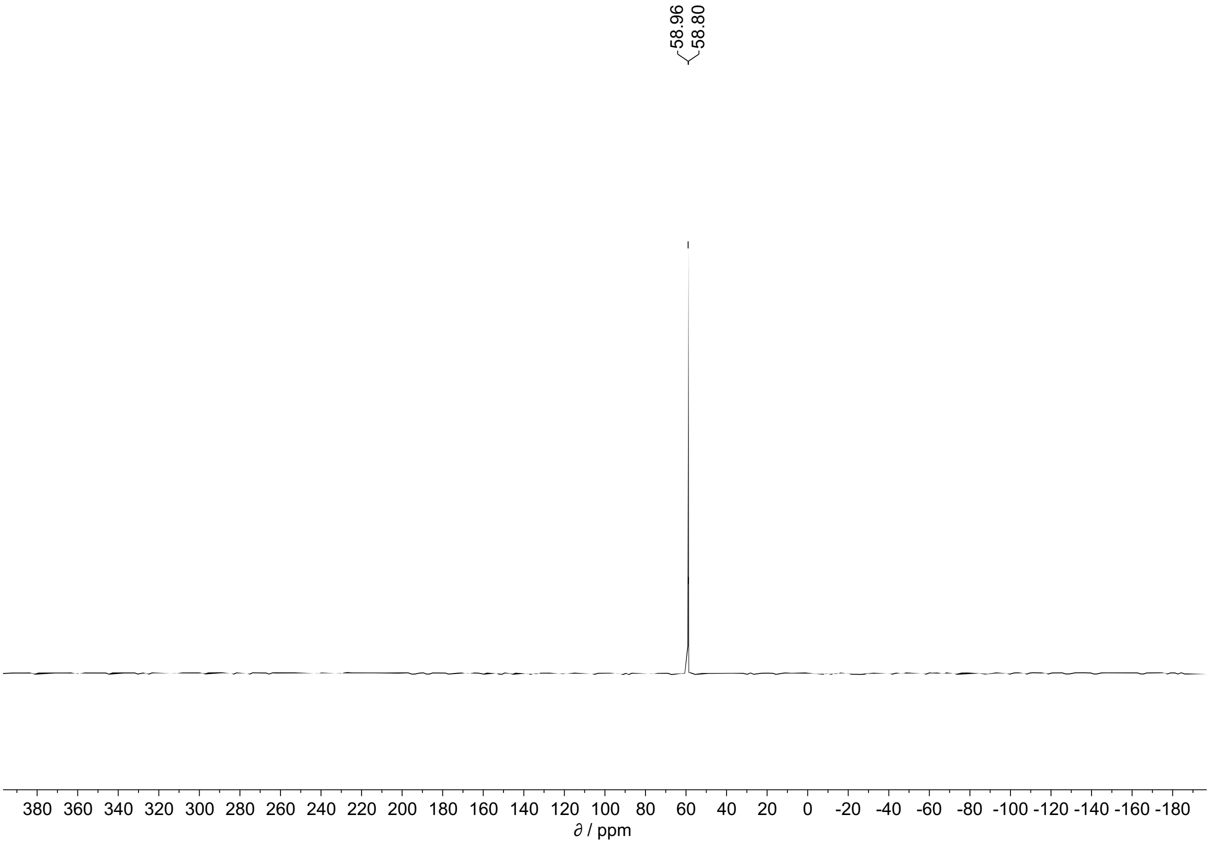

Figure S47. ^{31}P NMR spectrum (162 MHz, $CDCl_3$, 298 K) of **19**.

Synthesis of 20

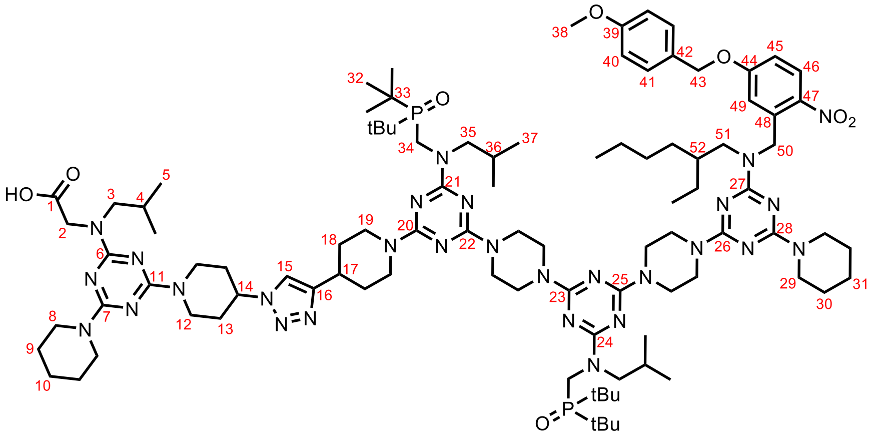

To a solution of **19** (57 mg, 0.038 mmol, 1 eq.) in DMF (4 mL), **12** (16 mg, 0.038 mmol, 1 eq.) and tetrakis(acetonitrile)copper(I) hexafluorophosphate (3 mg, 0.0076 mmol, 0.2 eq.) were added. The solution was heated at 90 °C under microwave irradiation and an atmosphere of nitrogen for 20 minutes. The solution was cooled to room temperature and diluted with ethyl acetate (20 mL) then washed with water (2 x 20 mL) and 5% lithium chloride solution (20 mL). The organic phase was dried over magnesium sulphate and the solvent removed under reduced pressure. The obtained residue was purified by flash chromatography (C4, 10-100% THF in H_2O). **20** was obtained as a white foam (50 mg, 0.026 mmol, 69%).

^{1}H NMR (700 MHz, CDCl$_3$): δ_H = 8.09 (br, 1H, H_{46}), 7.26 (br, 3H, $H_{15,41}$), 6.86 (m, 3H, $H_{40,45}$), 6.78 and 6.75 (rotamers, br, 1H, H_{49}), 5.09 (br, 2H, H_{50}), 4.97 (s, 2H, H_{43}), 4.93 – 4.73 (br, 4H, $H_{8,12,19,29}$ and protons of piperazine rings), 4.70 (br, 1H, H_{14}), 4.40 (br, 4H, H_{34}), 4.16 (s, 2H, H_2), 3.91 – 3.20 (br, 35H, $H_{3,8,12,19,29,35,38,51}$ and protons of piperazine rings), 3.02 (br, 1H, H_{17}), 2.96 (br, 4H, $H_{8,12,19,29}$ and protons of piperazine rings), 2.19 (br, 4H, $H_{13,36}$), 2.05 (br, 2H, H_{18}), 2.02 (m, 1H, H_4), 1.90 (br, 2H, H_{13}), 1.82 (br, 1H, H_{52}), 1.64 (br, 4H, $H_{9,10,18,30,31}$), 1.55 (br, 10H, $H_{9,10,30,31}$), 1.27 (br, 44H, H_{32} and CH_2 protons of alkyl chain), 0.88 (br, 24H, $H_{5,37}$ and methyl protons of alkyl chain);

^{13}C NMR (176 MHz, CDCl$_3$): δ_C = 172.2 (C_1), [166.2, 165.8 ($C_{6,21,24,27}$)], [165.6, 165.5, 165.5, 165.3, 165.2, 165.1, 165.0, 164.8, 164.5, 164.2 ($C_{7,11,20,22,23,25,26,28}$)], [163.0, 162.9 (rotamers, C_{44})], 159.8 (C_{39}), [152.1, 152.0 (rotamers, C_{16})], 141.5 (C_{47}), 139.6 (C_{48}), 129.6 (C_{41}), 127.8 (C_{46}), 127.7 (C_{42}), 117.1 (C_{15}), 114.2 ($C_{40,49}$), [112.8, 112.5 (rotamers, C_{45})], 70.4 (C_{43}), 58.6 (C_{14}), 56.4 (C_3), 55.4 (C_{38}), 53.1 (C_{35}), 52.2 (C_2), 51.0 (C_{51}), 49.0 (C_{50}), [44.5, 44.3, 44.1, 43.4, 43.2, 43.2, 43.1, 43.0, 42.4 ($C_{8,12,19,29}$ and carbons of piperazine rings)], 38.3 (C_{52}), 38.1 (d, $^1J_{CP}$ = 55.3 Hz, C_{34}), 35.8 (d, $^1J_{CP}$ = 55.2 Hz, C_{33}), 34.2 (C_{17}), 32.5 (C_{13}), 31.9 (C_{18}), [30.8, 29.8,

29.3, 28.9 (CH_2 carbons of alkyl chain)], 27.8 (C_4), 26.9 (C_{32}), 26.5 (C_{36}), [26.0, 25.8, 25.8, 25.1, 25.0, 25.0 ($C_{9,10,30,31}$)], [20.7, 20.6 (rotamers, $C_{5/37}$)], 20.4 ($C_{5/37}$), [14.3, 14.2, 10.9, 10.8 (methyl carbons of alkyl chain)];

^{31}P NMR (162 MHz, CDCl$_3$): δ_P = 59.7;

HRMS (ESI+): Calculated for $C_{97}H_{157}N_{28}O_8P_2^+$, 1904.2209; found 1904.2197;

FT-IR (ATR): ν_{max} /cm^{-1} 2951, 2928, 2854, 1731, 1612, 1527, 1480, 1431, 1387, 1368, 1345, 1287, 1253, 1208, 1175, 1134, 1023, 996, 937, 893, 834, 807, 735, 701, 644, 586, 505, 448.

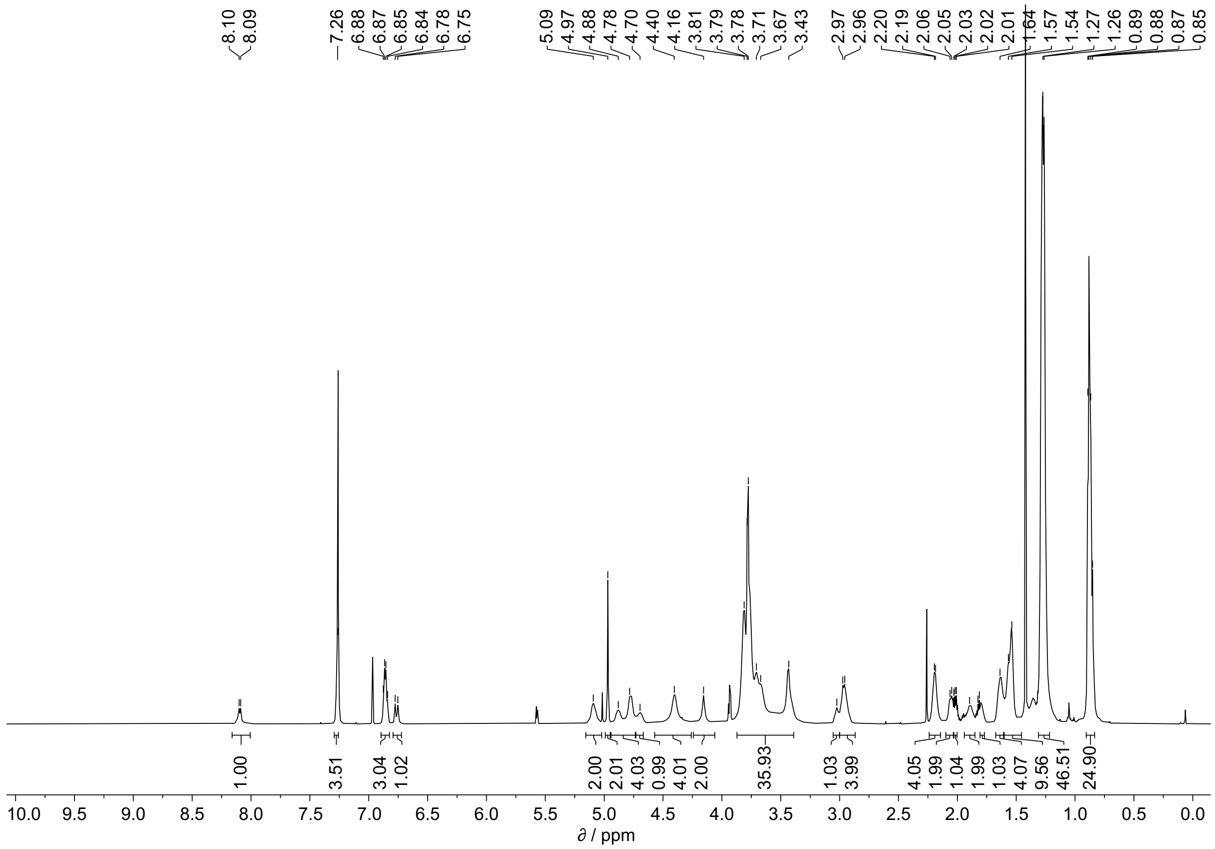

Figure S48. ^{1}H NMR spectrum (700 MHz, CDCl$_3$, 298 K) of **20**.

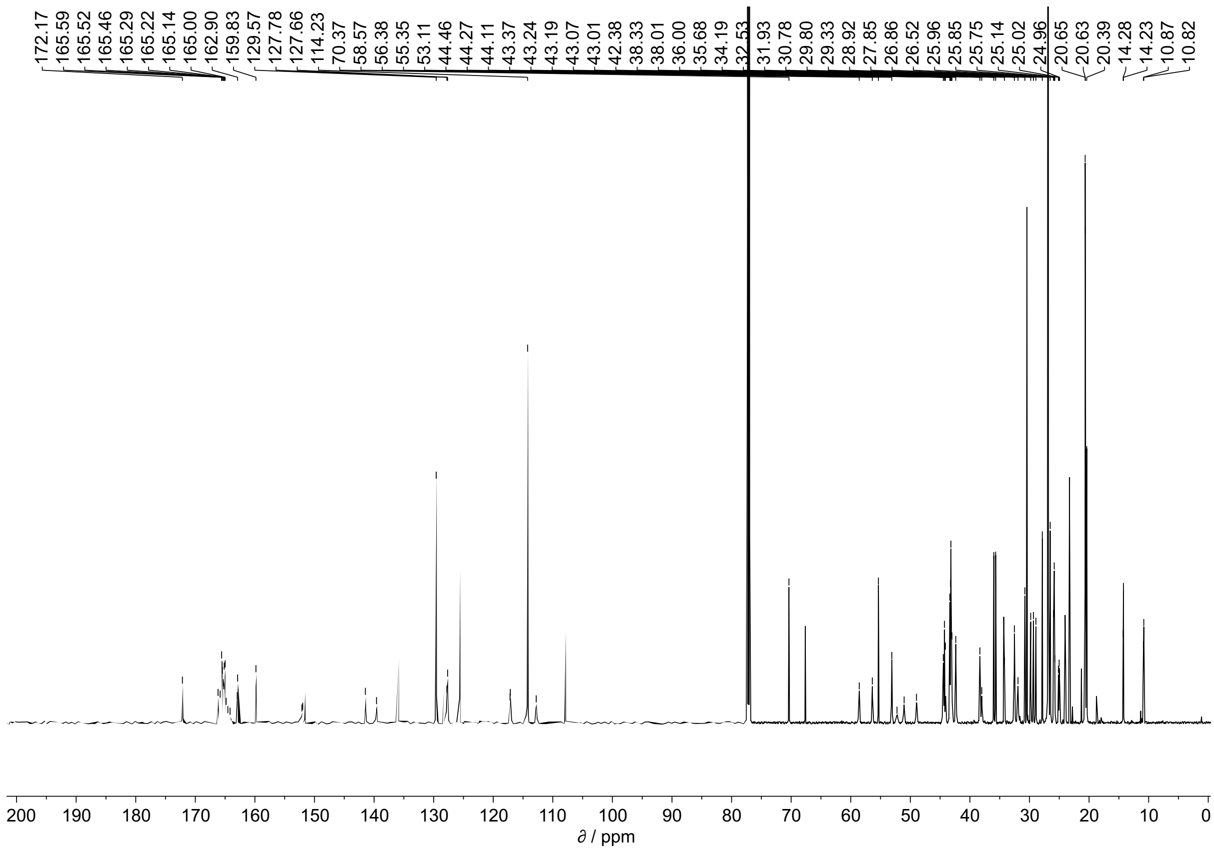

Figure S49. ^{13}C NMR spectrum (176 MHz, $CDCl_3$, 298 K) of **20**.

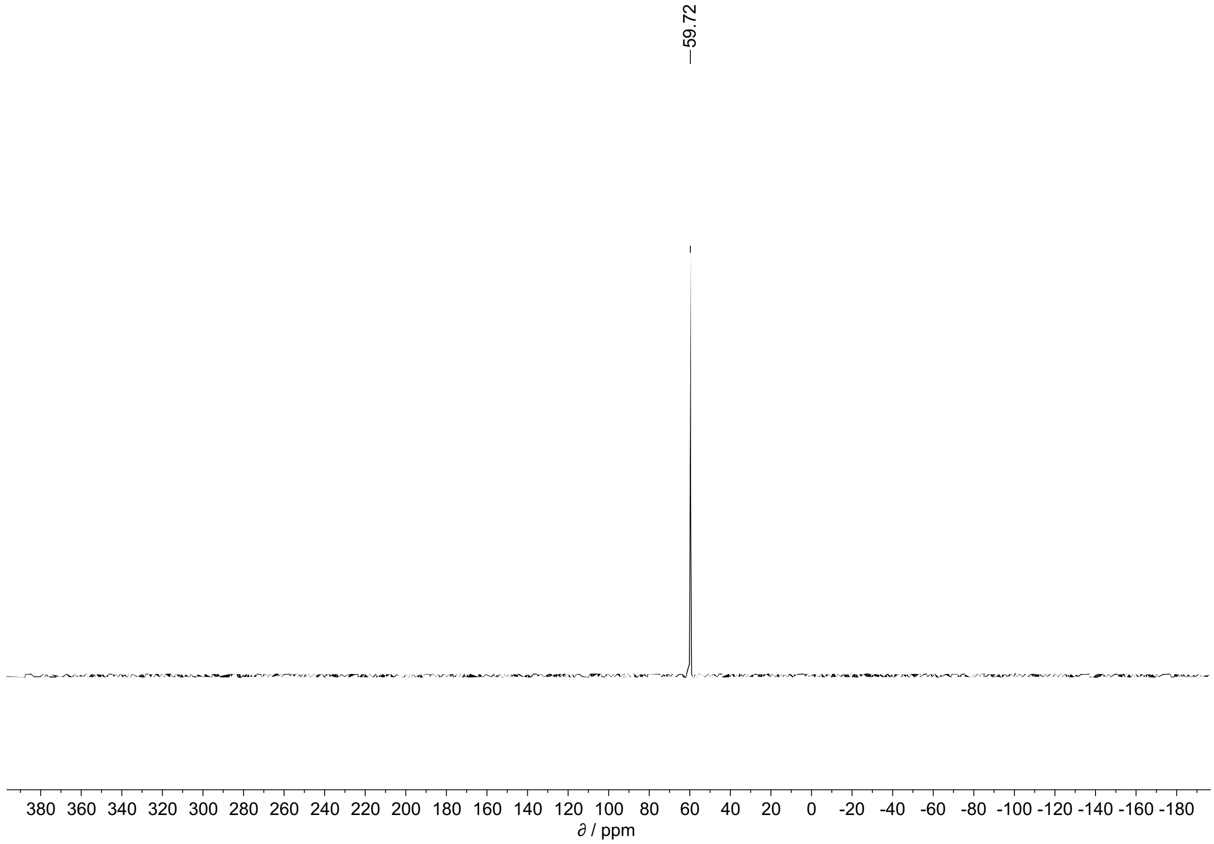

Figure S50. ^{31}P NMR spectrum (162 MHz, $CDCl_3$, 298 K) of **20**.

Synthesis of 21

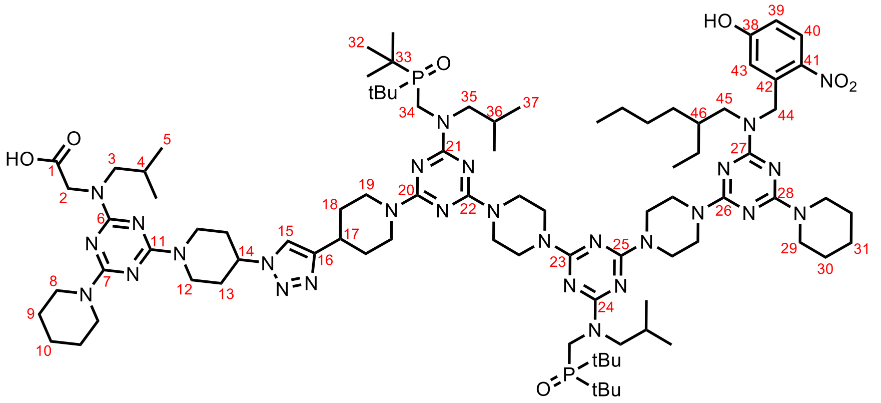

To a solution of **20** (50 mg, 0.026 mmol, 1.0 eq.) in DCM (4 mL), trifluoroacetic acid (4 mL) was added and the solution was stirred at room temperature for 5 minutes. The solvent was removed under reduced pressure and the residue was redissolved in ethyl acetate (10 mL). The organic phase was washed with water (10 mL) and the aqueous phase was extracted with ethyl acetate (10 mL). The combined organic phases were dried over magnesium sulphate and the solvent was removed under reduced pressure. The obtained residue was purified by flash chromatography (C4, 10-100% THF in H_2O). **21** was obtained as a white foam (37 mg, 0.021 mmol, 78%).

^{1}H NMR (700 MHz, CDCl$_3$): δ_H = 11.57 (br s, 1H, OH), 8.04 (br, 1H, H_{40}), 7.27 (br, 1H, H_{15}), 6.92 (br, 1H, H_{39}), 6.65 (br, 1H, H_{43}), 5.11 (br, 2H, H_{44}), 4.94 – 4.72 (br, 4H, $H_{8,12,19,29}$ and protons of piperazine rings), 4.70 (br, 1H, H_{14}), 4.43 (br, 4H, H_{34}), 4.15 (br, 2H, H_2), 3.91 – 3.29 (br, 32H, $H_{3,8,12,19,29,35,45}$ and protons of piperazine rings), 2.99 (br, 5H, $H_{8,12,17,19,29}$ and protons of piperazine rings), 2.19 (br, 4H, $H_{13,36}$), 2.05 (br, 2H, H_{18}), 2.02 (m, 1H, H_4), 1.90 (br, 2H, H_{13}), 1.83 (br, 1H, H_{46}), 1.64 (br, 4H, $H_{9,10,18,30,31}$), 1.54 (br, 10H, $H_{9,10,30,31}$), 1.28 (br, 44H, H_{32} and CH_2 protons of alkyl chain), 0.89 (d, J = 6.7 Hz, 6H, H_5), 0.85 (br, 18H, H_{37} and methyl protons of alkyl chain);

^{13}C NMR (176 MHz, CDCl$_3$): δ_C = 172.3 (C_1), 166.2 ($C_{6,21,24,27}$), [165.8, 165.6, 165.2, 165.0, 164.8 ($C_{7,11,20,22,23,25,26,28}$)], 163.9 ($C_{38}$), 152.0 ($C_{16}$), 139.9 ($C_{42}$), 128.1 ($C_{40}$), 117.2 ($C_{15}$), 115.0 ($C_{43}$), 114.0 ($C_{39}$), 58.6 ($C_{14}$), 56.5 ($C_3$), 53.2 ($C_{35}$), 52.3 ($C_2$), 51.0 ($C_{45}$), 49.0 ($C_{44}$), [44.5, 44.2, 44.1, 43.4, 43.2, 42.4 ($C_{8,12,19,29}$ and carbons of piperazine rings)], 38.3 (C_{46}), 38.1 (d, $^1J_{CP}$ = 54.6 Hz, C_{34}), 35.8 (d, $^1J_{CP}$ = 54.8 Hz, C_{33}), 34.2 (C_{17}), 32.5 (C_{13}), 31.9 (C_{18}), [30.8, 30.4, 29.8, 29.0 (CH_2 carbons of alkyl chain)], 27.9 (C_4), 26.9 (C_{32}), 26.5 (C_{36}), [26.0, 25.9, 25.8, 25.2, 25.1, 25.0 ($C_{9,10,30,31}$)], [24.03, 23.30 (CH_2 carbons of alkyl chain)], [20.6, 20.6 (C_{37})], 20.4 (C_5), [14.3, 14.3, 10.9, 10.9 (methyl carbons of alkyl chain)]; (C_{41} unassigned);

^{31}P NMR (162 MHz, CDCl$_3$): δ_P = 61.5;

HRMS (ESI+): Calculated for $C_{89}H_{149}N_{28}O_7P_2^+$, 1784.1634; found 1784.1647;

FT-IR (ATR): ν_{max} /cm^{-1} 2953 (br), 2928, 2868, 2854, 1730, 1530, 1481, 1431, 1368, 1339, 1305, 1255, 1208, 1129, 1023, 995, 836, 808, 736, 646, 497.

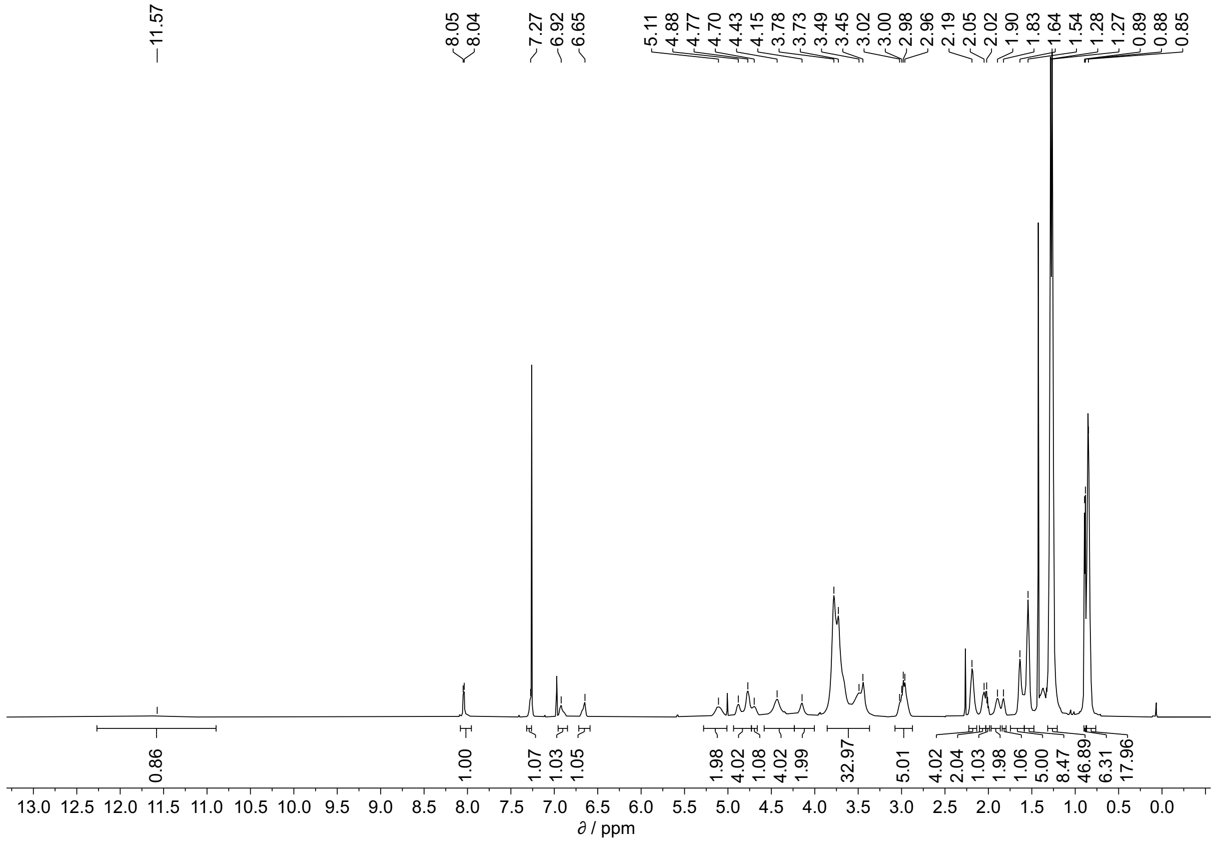

Figure S51. ^{1}H NMR spectrum (700 MHz, CDCl$_3$, 298 K) of **21**.

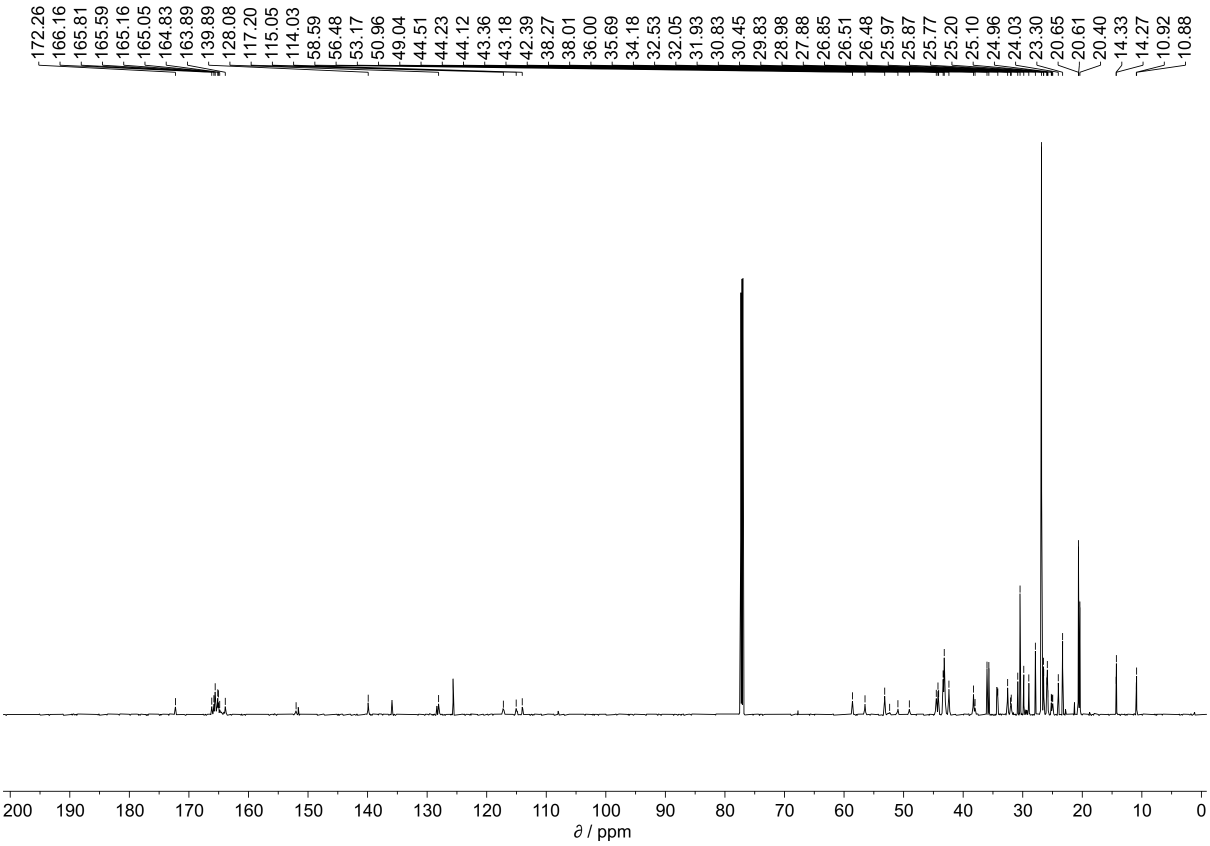

Figure S52. ^{13}C NMR spectrum (176 MHz, $CDCl_3$, 298 K) of **21**.

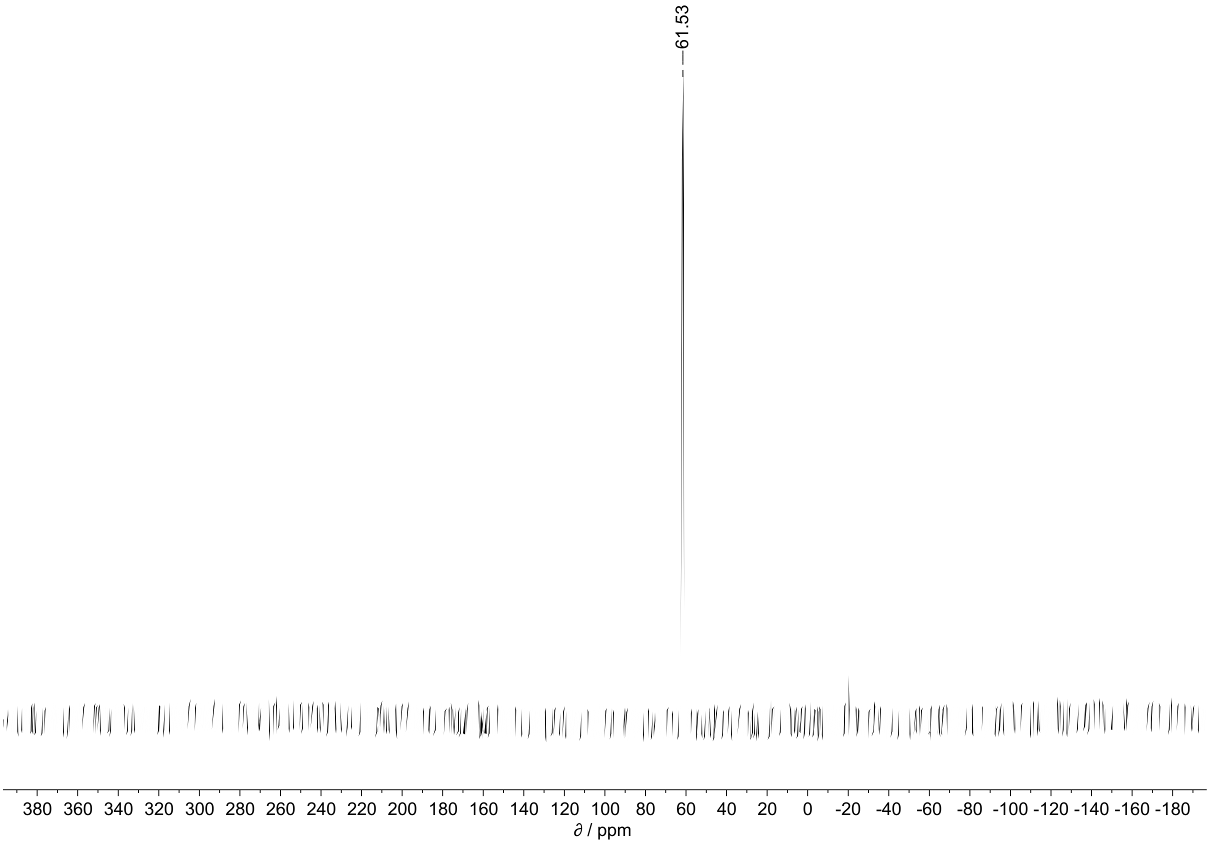

Figure S53. ^{31}P NMR spectrum (162 MHz, $CDCl_3$, 298 K) of **21**.

Synthesis of 22

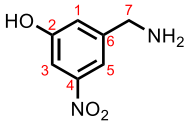

To a solution of 3-hydroxy-5-nitrobenzonitrile (710 mg, 4.3 mmol, 1 eq.) in dry THF (14 mL) at 0 °C, a solution of borane in THF (13 mmol, 13 mL, 3 eq.) was added dropwise. The reaction was stirred overnight at room temperature under an atmosphere of nitrogen. The solution was cooled to 0 °C and a solution of hydrogen chloride in methanol (1 M, 13 mL) was added dropwise. The solution was azeotroped and the obtained residue was purified by silica flash chromatography (DCM / methanol (8:2)). **22** was obtained as orange crystals (640 mg, 3.8 mmol, 88%).

^{1}H NMR (400 MHz, MeOD): δ_H = 7.78 (t, J = 1.8 Hz, 1H, H_5), 7.58 (t, J = 2.2 Hz, 1H, H_3), 7.24 (t, J = 1.9 Hz, 1H, H_1), 4.07 (s, 2H, H_7);

^{13}C NMR (101 MHz, MeOD): δ_C = 160.7 (C_2), 150.9 (C_4), 139.9 (C_6), 123.1 (C_1), 114.7 (C_5), 111.1 (C_3), 44.3 (C_7);

HRMS (ESI+): Calculated for $C_7H_9N_2O_3^+$, 169.0608; found 169.0607;

FT-IR (ATR): ν_{max} /cm^{-1} 3411 (br), 3239 (br), 3081, 2976, 2898, 1612, 1588, 1522, 1342, 1291, 1234, 1170, 1093, 1011, 962, 892, 780, 747, 666.

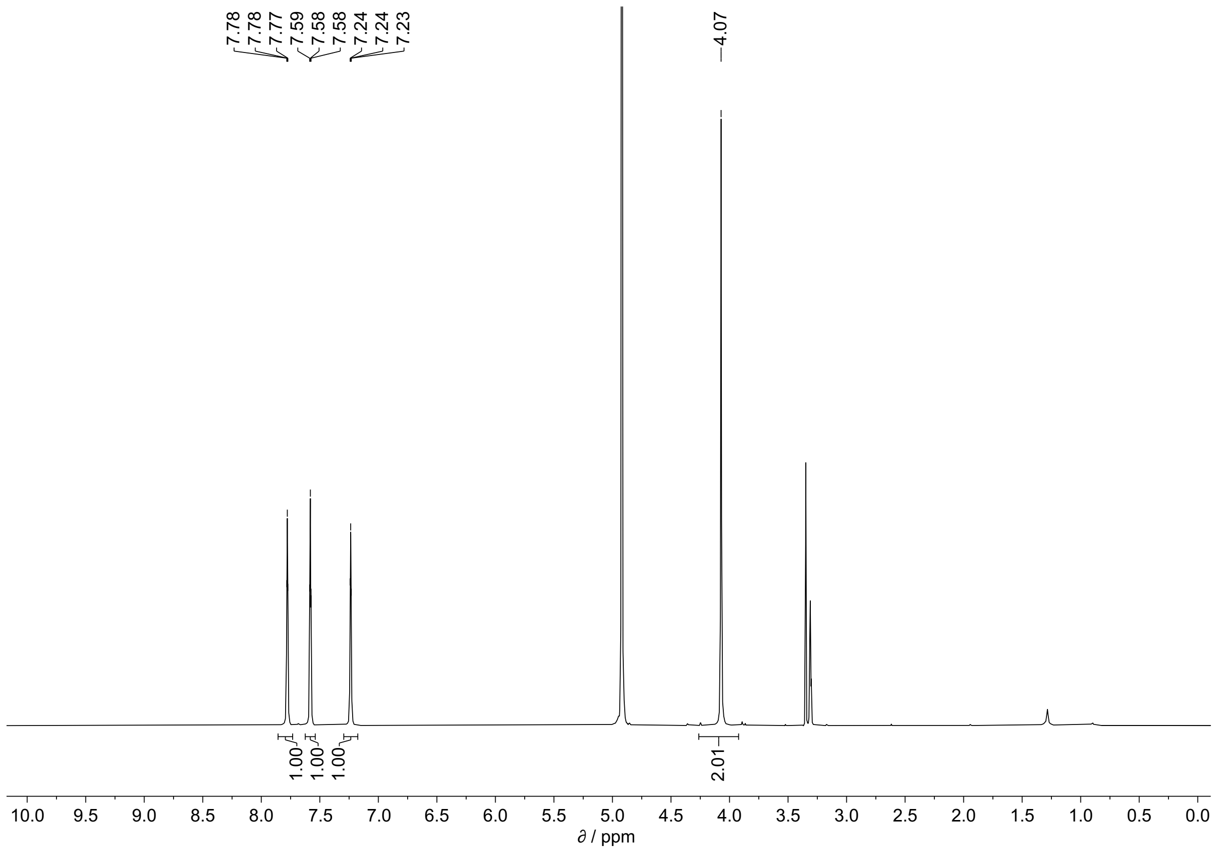

Figure S54. ^{1}H NMR spectrum (400 MHz, MeOD, 298 K) of **22**.

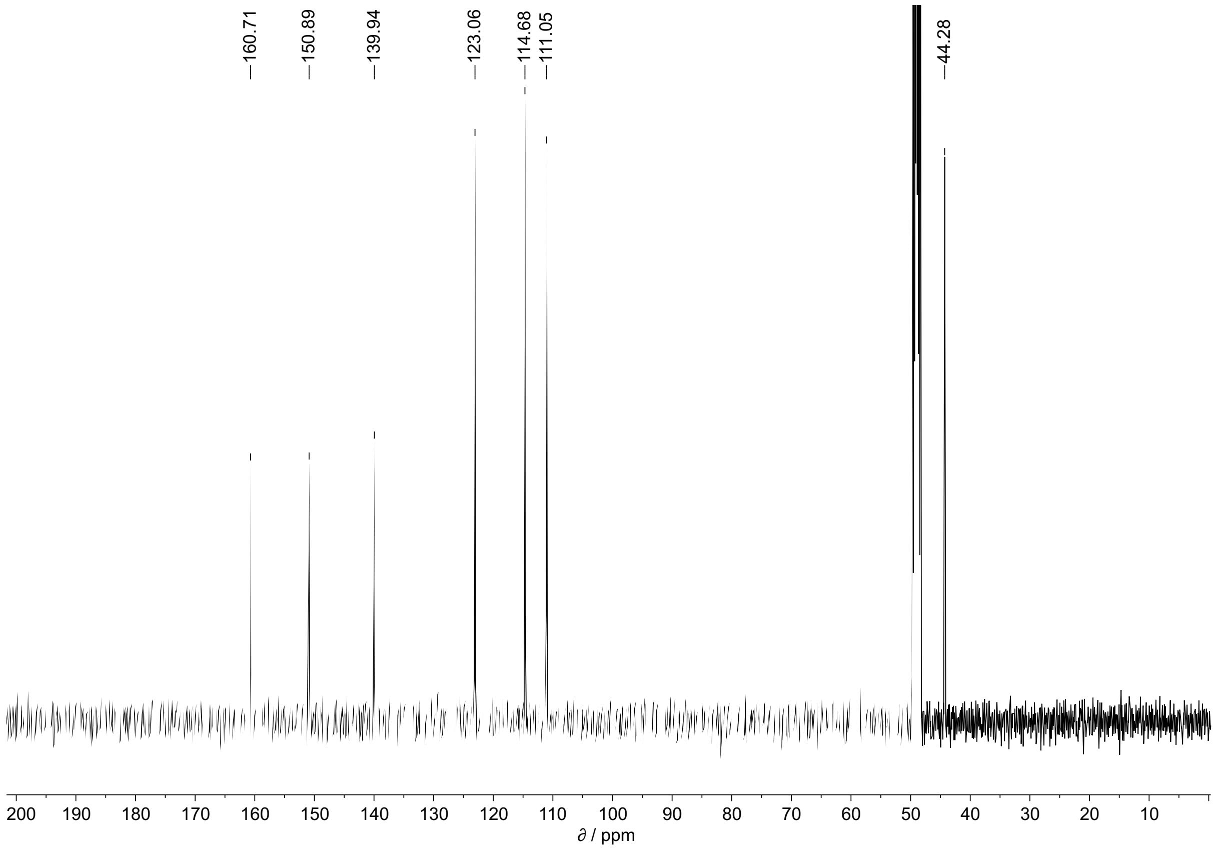

Figure S55. ^{13}C NMR spectrum (101 MHz, MeOD, 298 K) of **22**.

Synthesis of 23

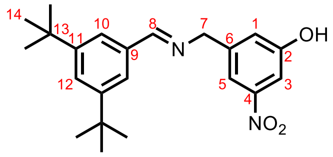

To a solution of 3,5-di-*tert*-butylbenzyaldehyde (50 mg, 0.23 mmol, 1 eq.) in ethanol (6 mL), **22** (190 mg, 1.2 mmol, 5 eq.) and molecular sieves (4 Å) were added, and the solution was heated under reflux overnight. The solution was cooled to room temperature and filtered, then the solvent was removed under reduced pressure. The obtained residue was suspended in toluene (5 mL) and filtered. The filtrate was collected and the solvent was removed under reduced pressure. **23** was obtained as an orange foam (34 mg, 0.092 mmol, 40%).

^{1}H NMR (400 MHz, CDCl$_3$): δ_H = 8.45 (s, 1H, H_8), 7.64 (s, 3H, $H_{5,10}$), 7.56 (s, 1H, H_{12}), 7.36 (s, 1H, H_3), 7.03 (s, 1H, H_1), 4.75 (s, 2H, H_7), 1.29 (s, 18H, H_{14});

^{13}C NMR (101 MHz, CDCl$_3$): δ_C = 166.7 (C_8), 157.7 (C_2), 151.9 (C_{11}), 149.4 (C_4), 140.8 (C_6), 133.9 (C_9), 126.6 (C_{12}), 123.3 (C_{10}), 122.2 (C_1), 114.7 (C_5), 110.3 (C_3), 64.0 (C_7), 35.1 (C_{13}), 31.4 (C_{14});

HRMS (ESI+): Calculated for $C_{22}H_{29}N_2O_3^+$, 369.2173; found 369.2167;

FT-IR (ATR): ν_{max} /cm^{-1} 3000 (br), 2962, 2904, 2867, 1639, 1595, 1530, 1477, 1450, 1393, 1349, 1289, 1248, 1204, 1156, 1089, 1038, 1006, 968, 934, 897, 874, 820, 784, 747, 739, 703, 686.

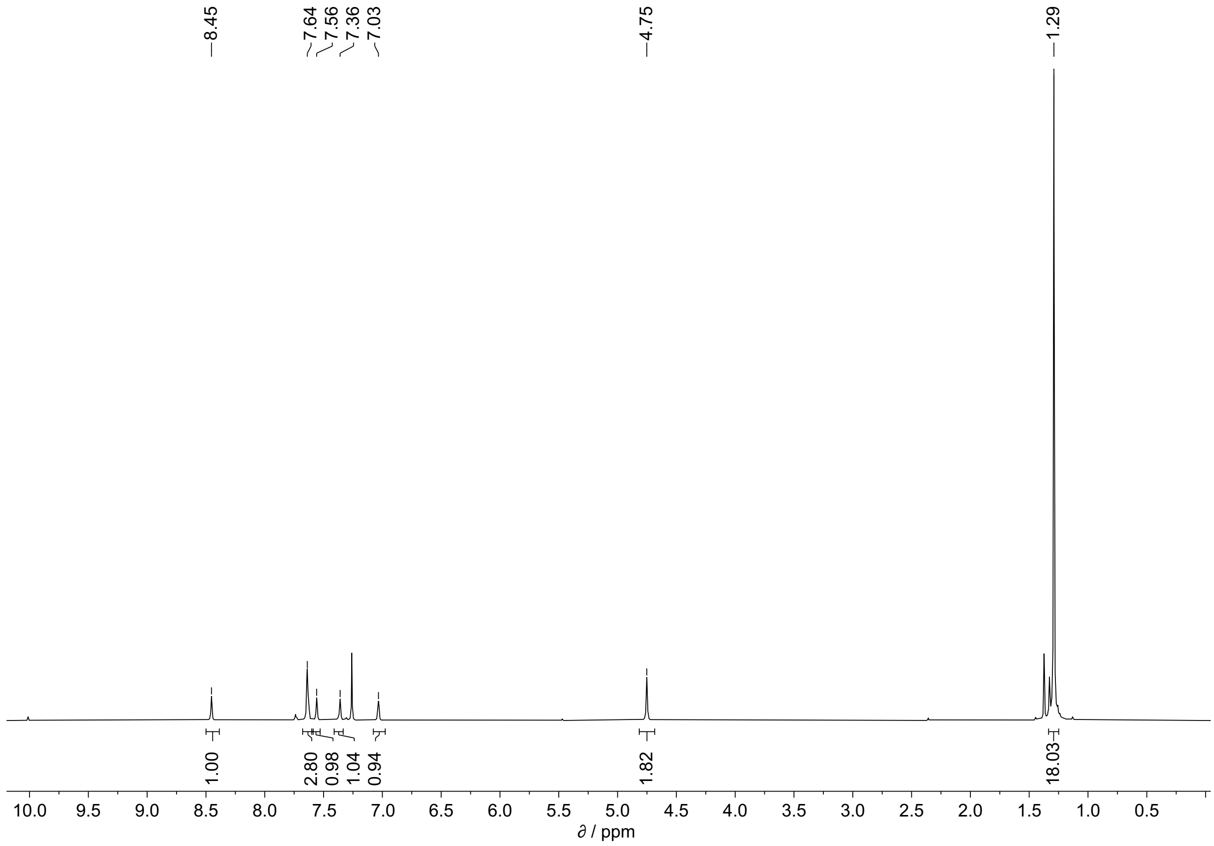

Figure S56. ^{1}H NMR spectrum (400 MHz, $CDCl_3$, 298 K) of **23**.

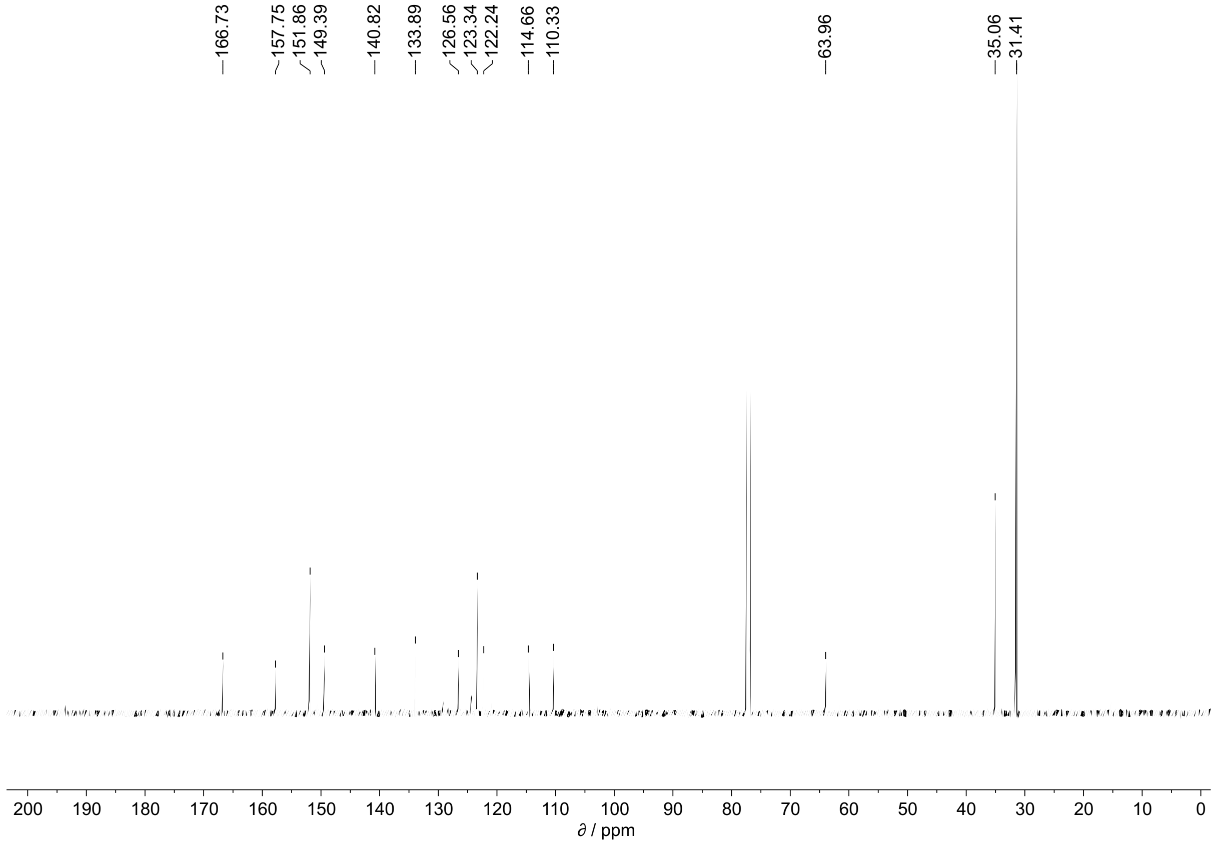

Figure S57. ^{13}C NMR spectrum (101 MHz, $CDCl_3$, 298 K) of **23**.

Synthesis of 24

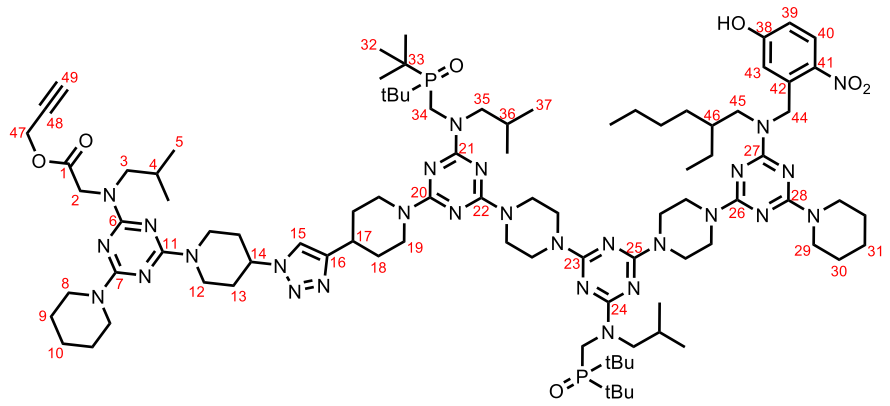

To a solution of **21** (36.9 mg, 0.0207 mmol, 1 eq.) in dry DCM (2 mL), propargyl alcohol (24 µL, 0.414 mmol, 20 eq.), EDC (19.8 mg, 0.104 mmol, 5 eq.) and DMAP (25.3 mg, 0.207 mmol, 10 eq.) were added and the solution was stirred at room temperature overnight under nitrogen. The solution was diluted with DCM (5 mL) and washed with hydrochloric acid solution (0.1 M, 3 x 5 mL). The organic layer was dried over magnesium sulphate and the solvent was removed under reduced pressure. The obtained residue was purified by silica flash chromatography (DCM/methanol (95:5)). **24** was obtained as a white foam (26.5 mg, 0.0145 mmol, 70%).

^{1}H NMR (700 MHz, CDCl$_3$): δ_H = 11.57 (br s, 1H, OH), 8.04 (br, 1H, H_{40}), 7.27 (br, 1H, H_{15}), 6.93 (br, 1H, H_{39}), 6.68 and 6.65 (rotamers, s, 1H, H_{43}), 5.12 (br, 2H, H_{44}), 4.91 and 4.98 (rotamers, br, 1H, H_{12}), 4.87 – 4.72 (br, 3H, $H_{8,12,19,29}$ and protons of piperazine rings), 4.69 (br, 1H, H_{14}), 4.69 and 4.67 (rotamers, d, J = 2.5 Hz, 2H, H_{47}), 4.43 (br s, 4H, H_{34}), 4.15 and 4.14 (rotamers, s, 2H, H_2), 3.91 – 3.46 (br, 30H, $H_{8,12,19,29,35,45}$ and protons of piperazine rings), 3.44 and 3.43 (rotamers, d, J = 7.2 Hz, 2H, H_3) 3.01 (br, 1H, H_{17}), 2.99 – 2.86 (br, 4H, $H_{8,12,19,29}$ and protons of piperazine rings), 2.46 and 2.43 (rotamers, t, J = 2.5 Hz, 1H, H_{49}), 2.19 (br, 4H, $H_{13,36}$), 2.06 (br, 2H, H_{18}), 1.98 (non, J = 6.7 Hz, 1H, H_4), 1.85 (br, 3H, $H_{13,46}$), 1.62 (br, 6H, $H_{9,10,18,30,31}$), 1.53 (br, 8H, $H_{9,10,30,31}$), 1.27 (br, 44H, H_{32} and CH_2 protons of alkyl chain), 0.92 and 0.91 (rotamers, d, J = 5.6 Hz, 6H, H_5), 0.86 (br, 18H, H_{37} and methyl protons of alkyl chain);

^{13}C NMR (176 MHz, CDCl$_3$): δ_C = [170.4, 170.4 (rotamers, C_1)], [166.2, 166.2, 166.1 ($C_{6,21,24,27}$)], [165.6, 165.5, 165.3, 165.3, 165.2, 165.1, 165.0, 164.8 ($C_{7,11,20,22,23,25,26,28}$)], 163.9 ($C_{38}$), 151.9 ($C_{16}$), 139.9 ($C_{42}$), 128.1 ($C_{40}$), 117.1 ($C_{15}$), [115.0, 114.9 (rotamers, C_{43})], [114.0, 113.9 (rotamers, C_{39})], 77.7 (C_{48}), [75.2, 75.1 (rotamers, C_{49})], 58.9 (C_{14}), 55.9 (C_3), 53.2 (C_{35}), 52.2 (C_{47}), 51.0 (C_{45}), 50.5 (C_2), 49.0 (C_{44}), [44.3, 44.2, 44.1, 43.3, 43.2, 42.2, 42.2 ($C_{8,12,19,29}$

and carbons of piperazine rings)], 38.3 (C_{46}), 38.1 (d, $^1J_{CP}$ = 58.1 Hz, C_{34}), 35.9 (d, $^1J_{CP}$ = 55.0 Hz, C_{33}), 34.2 (C_{17}), [32.6, 32.6 (rotamers, C_{13})], 32.0 (C_{18}), [30.8, 29.8, 29.0 (CH_2 carbons of alkyl chain)], [28.2, 28.2 (rotamers, C_4)], 26.9 (C_{32}), [26.5, 26.5 (C_{36})], [26.0, 25.9, 25.9, 25.8, 25.2, 25.1, 25.1 ($C_{9,10,30,31}$)], [24.0, 23.3 (CH_2 carbons of alkyl chain)], 20.7 (C_{37}), [20.5, 20.5 (rotamers, C_5)], [14.3, 14.3, 10.9, 10.9 (methyl carbons of alkyl chain)]; (C_{41} unassigned);

^{31}P NMR (162 MHz, $CDCl_3$): δ_P = 60.7;

HRMS (ESI+): Calculated for $C_{92}H_{151}N_{28}O_7P_2^+$, 1822.1790; found 1822.1807;

FT-IR (ATR): ν_{max} 3293, 2952, 2927, 2853, 1759, 1529, 1481, 1431, 1368, 1339, 1305, 1255, 1209, 1171, 1127, 1023, 995, 833, 807, 735, 650, 502, 451.

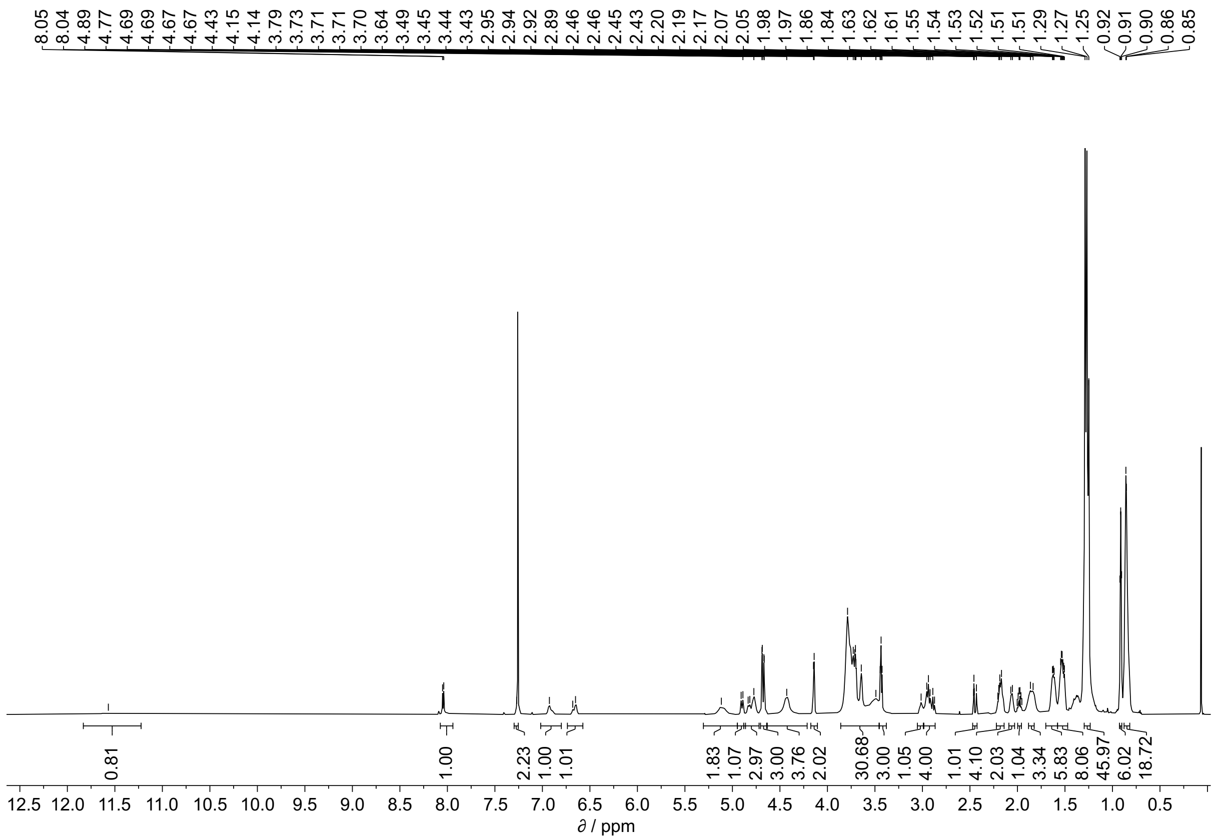

Figure S58. ^{1}H NMR spectrum (700 MHz, $CDCl_3$, 298 K) of **24**.

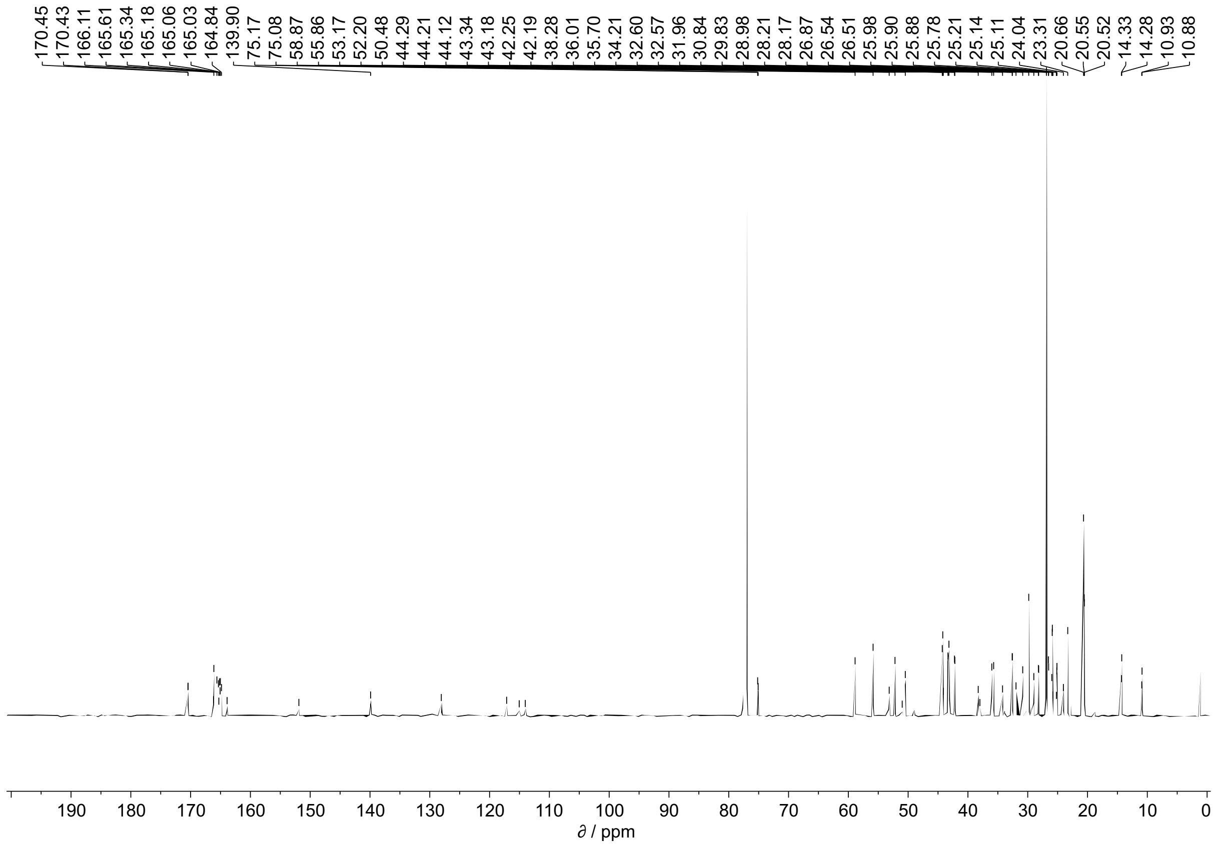

Figure S59. ^{13}C NMR spectrum (176 MHz, $CDCl_3$, 298 K) of **24**.

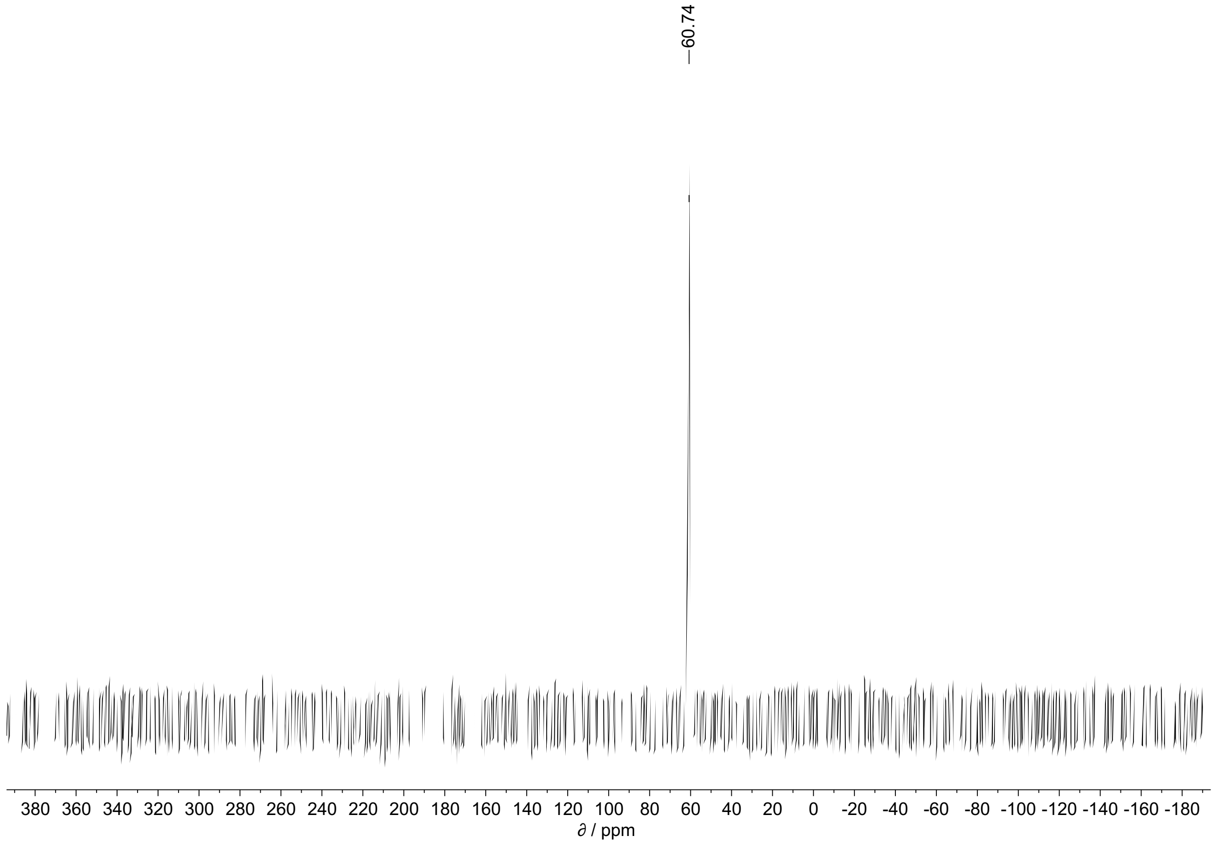

Figure S60. ^{31}P NMR spectrum (162 MHz, $CDCl_3$, 298 K) of **24**.

Synthesis of 25

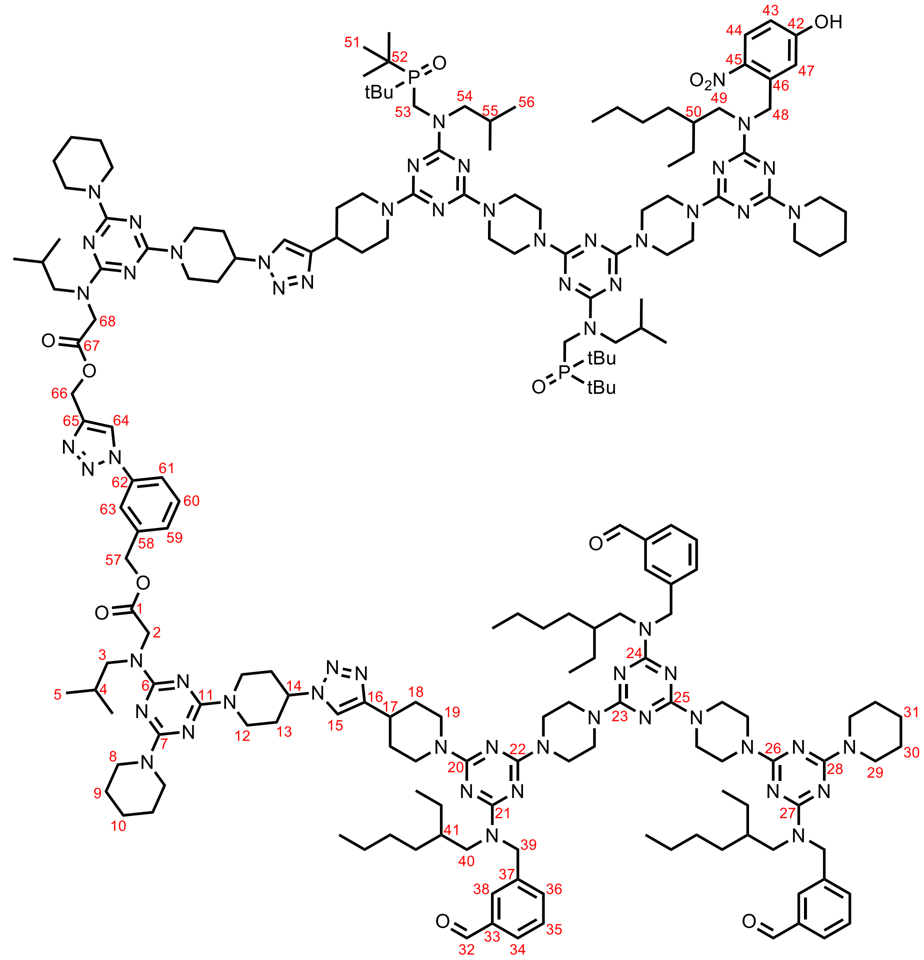

To a solution of **24** (18.0 mg, 0.00989 mmol, 1.00 eq.) in dry THF (1 mL), **13** (18.6 mg, 0.00989 mmol, 1.00 eq.), tetrakis(acetonitrile)copper(I) hexafluorophosphate (3.7 mg, 0.0099 mmol, 1.0 eq.) and TBTA (10 mg, 0.020 mmol, 2.0 eq.) were added. The solution was stirred at room temperature overnight under an atmosphere of nitrogen. The solvent was removed under reduced pressure and the obtained residue was redissolved in ethyl acetate (5 mL). The organic layer was washed with water (5 mL) and EDTA solution (0.01 M, 5 mL), dried with magnesium sulphate and the solvent removed under reduced pressure. **25** was obtained as a pale yellow foam and used without further purification (57.5 mg, quantitative; containing some TBTA as impurity).

^{1}H NMR (700 MHz, CDCl$_3$): δ_H = [9.97, 9.95 (s, 3H, H_{32})], 8.04 (br, 1H, H_{44}), [7.95, 7.94 (rotamers, s, 1H, H_{64})], 7.75 (br, 6H, $H_{34,38}$), 7.52 (br, 3H, H_{36}), 7.47 (br, 1H, H_{60}), 7.43 (br, 3H, H_{35}), 7.39 (br, 1H, H_{59}), 6.91 (br, 1H, H_{43}), 6.67 (br, 1H, H_{47}), [5.33, 5.32 (rotamers, s, 2H, H_{66})], [5.20, 5.18 (rotamers, s, 2H, H_{57})], 5.12 (br, 2H, H_{48}), 4.93 – 4.50 (br, 16H, $H_{8,12,14,19,29,39}$ and protons of piperazine rings), 4.43 (br, 4H, H_{53}), 4.18 (s, 2H, H_{68}), 4.15 (s, 2H, H_2), 3.89 –

3.37 (br, 64H, $H_{3,8,12,19,29,40,49,54}$ and protons of piperazine rings), 3.05 – 2.62 (br, 10H, $H_{8,12,17,19,29}$ and protons of piperazine rings), 2.17 (br, 6H, $H_{13,55}$), 2.06 (br, 6H, H_{18}), 1.98 (br, 2H, H_3), 1.91 (br, 4H, H_{13}), 1.79 (br, 4H, $H_{41,50}$), 1.60 (br, 10H, $H_{9,10,18,30,31}$), 1.26 (br, 16H, $H_{9,10,30,31}$), 1.26 (br, 68H, H_{51} and CH_2 protons of alkyl chains), [0.90, 0.85 (br, 48H, $H_{5,56}$ and methyl protons of alkyl chains); (H_{15}, H_{61}, H_{63} obscured by TBTA peaks);

^{13}C NMR (176 MHz, $CDCl_3$): δ_C = [192.6, 192.5 (C_{32})], [171.3, 171.2, 171.2 (rotamers, C_{67})], [170.9, 170.9 (rotamers, C_1)], [166.3, 166.1 ($C_{6,21,24,27}$)], [165.6, 165.3, 165.1, 165.0, 165.0, 164.8, 164.8 ($C_{7,11,20,22,23,25,26,28}$)], 163.7 ($C_{42}$), [152.1, 151.8 ($C_{16}$)], 143.8 ($C_{65}$), [141.3, 141.2, 141.2, 141.1, 141.0 (C_{37})], 139.9 (C_{46}), [138.3, 138.3, 138.2 (rotamers, C_{58})], [137.1, 137.0 (rotamers, C_{62})], 136.6 (C_{33}), [133.8, 133.8 (C_{36})], [130.1, 130.1 (rotamers, C_{60})], [129.1, 129.1, 129.0 (C_{35})], [128.7, 128.5, 128.5, 128.3 ($C_{34,38}$)], [122.2, 122.1 (rotamers, C_{64})], [120.1, 120.1 (rotamers, C_{61})], [120.0, 119.9 (rotamers, C_{63})], 117.3 (C_{15}), 115.0 (C_{47}), 114.0 (C_{43}), 65.4 (C_{57}), [58.8, 58.7 (C_{14})], 57.9 (C_{66}), 55.9 (C_3), 53.2 (C_{54}), 51.0 (C_{49}), 50.7 (C_{68}), 50.5 (C_2), 50.0 ($C_{39,40}$), 49.0 (C_{48}), [44.3, 44.3, 44.2, 44.2, 44.1, 43.4, 43.2, 42.2, 42.0 ($C_{8,12,19,29}$ and carbons of piperazine rings)], 38.1 (d, $^1J_{CP}$ = 50.9 Hz, C_{53}), 38.0 ($C_{41,50}$), 35.8 (d, $^1J_{CP}$ = 55.1 Hz, C_{52}), 34.2 (C_{17}), [32.5, 32.4 (C_{13})], [32.0, 31.9 (C_{18})], [30.8, 30.3, 29.8, 29.7, 29.5, 29.0 (CH_2 carbons of alkyl chains)], [28.2, 28.1 (C_4)], [26.8, 26.5 (CH_2 carbons of alkyl chains)], [25.9, 25.9, 25.8, 25.8, 25.1, 25.1, 25.0, 25.0 ($C_{9,10,30,31}$)], [24.0, 23.3, 23.3, 22.8 (CH_2 carbons of alkyl chains)], [20.6, 20.6, 20.5 ($C_{5,56}$)], [14.3, 14.3, 14.2, 10.9 (methyl carbons of carbon chains)]; (C_{45} unassigned, C_{44} obscured by TBTA peak);

^{31}P NMR (162 MHz, $CDCl_3$): δ_P = 60.9;

HRMS (ESI+): Calculated for $C_{195}H_{296}N_{58}O_{12}P_2^{2+}$, 1853.1933; found 1853.1981;

FT-IR (ATR): ν_{max} /cm^{-1} 2954, 2924, 2853, 1750, 1700, 1529, 1483, 1433, 1368, 1306, 1255, 1178, 1132, 1104, 1047, 1025, 996, 844, 807, 722, 698.

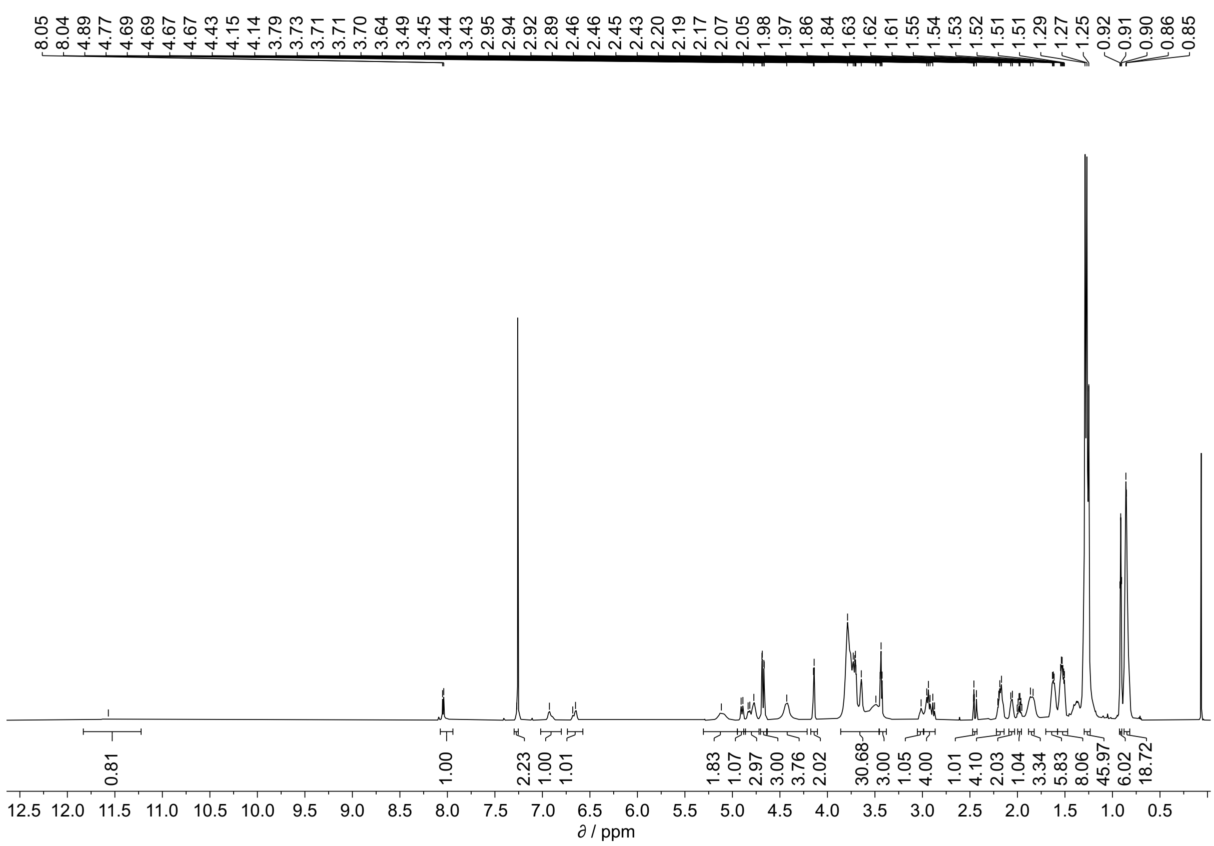

Figure S61. ^{1}H NMR spectrum (700 MHz, $CDCl_3$, 298 K) of **25**.

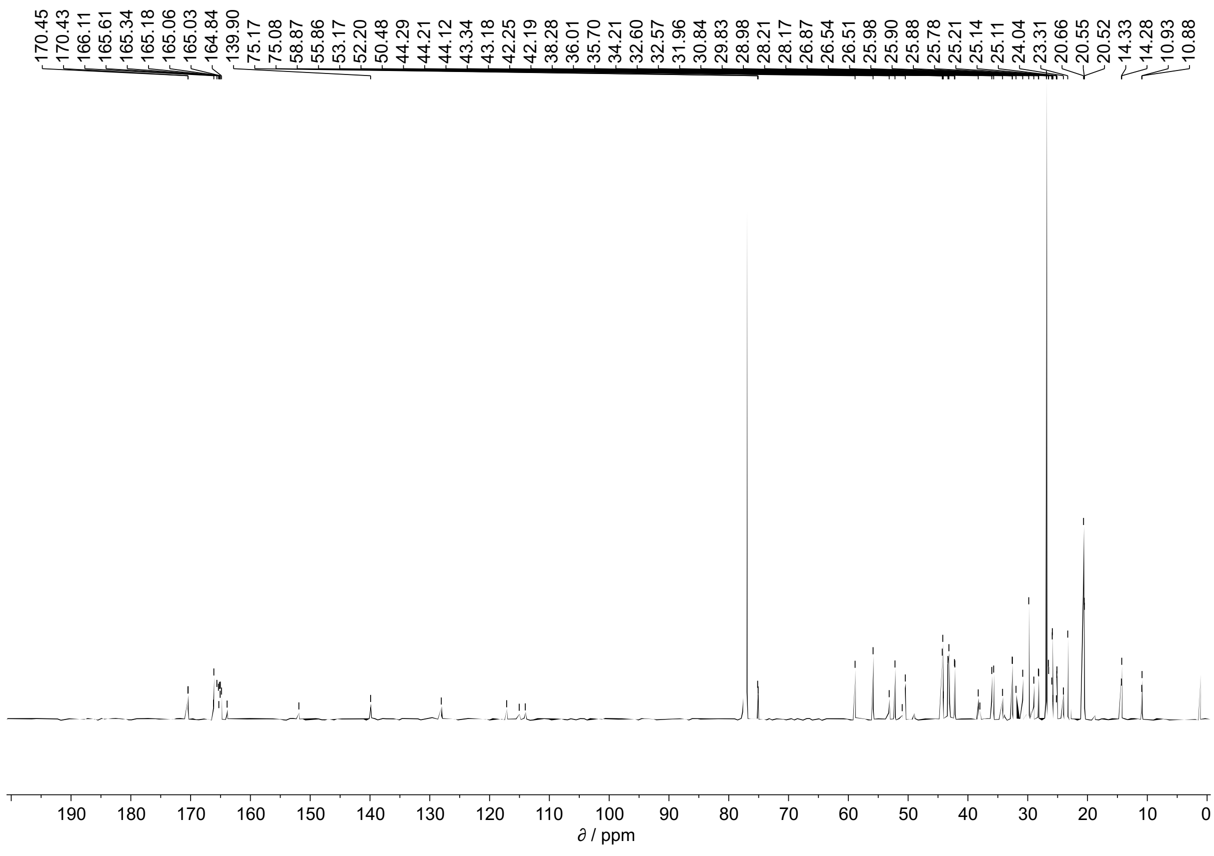

Figure S62. ^{13}C NMR spectrum (176 MHz, $CDCl_3$, 298 K) of **25**.

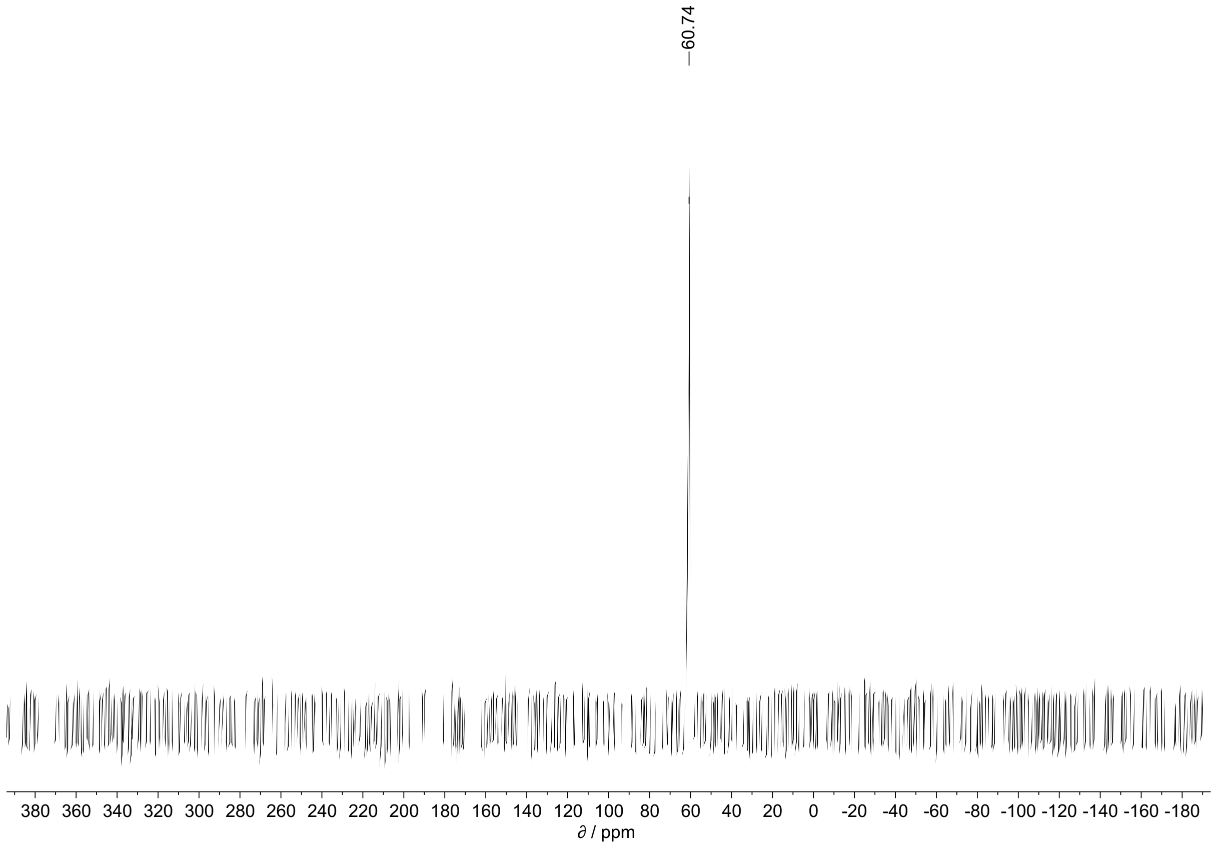

Figure S63. ^{31}P NMR spectrum (162 MHz, $CDCl_3$, 298 K) of **25**.

b. Imine attachment, trapping and cleavage

Stock solutions of **14**, **23** and **25** were made up in DCM-d_2 with a TCE spike of known concentration, and stock concentrations were determined by ^{1}H NMR spectroscopy using the spike as a reference. Calculated amounts of **25** (1 eq.), **14** (12 eq.) and **23** (12 eq.) were mixed and diluted with a calculated volume of DCM-d_2 to give a concentration of **25** of 1.0 mM in 250 µL. The reactions were left for 1 day to equilibrate and a ^{1}H NMR spectrum was recorded to verify complete imine formation (Figure S63). The DCM was removed by flushing under a stream of nitrogen. The residue was redissolved in toluene (2.5 mL) and re-equilibrated for 1 day. To aliquots (1 mL) of the equilibrated mixture, trichlorosilane (0.5 M) and DMF (0.5 M) were added and the solution was stirred vigorously for 5 minutes. The solvent was removed under a stream of nitrogen and the obtained residue was redissolved in ethyl acetate (1 mL). Sodium hydrogen carbonate (1 mL) was added and the solution shaken vigorously. To the emulsion, potassium fluoride (5 mg) was added and the solution was stirred vigorously for 1 hour. The organic phase was collected and solvent was removed under a stream of nitrogen. The sample was redissolved in DMSO (200 µL) and the copy strands separated by semi-preparative HPLC.

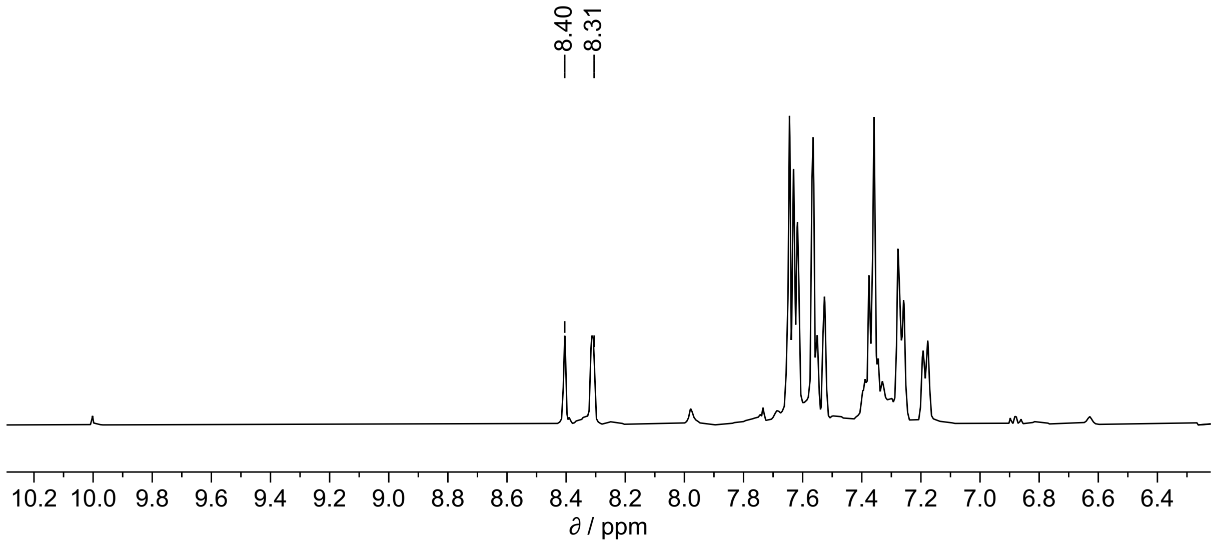

Figure S64. Partial ^{1}H NMR spectrum (400 MHz, CD_2Cl_2, 298 K) of the equilibrated mixture of **25** (1.0 mM) and **14** and **23** (each 12 mM).

c. Relative imine stabilities

14 (1.0 mM) was mixed with **23** (1.0 mM) in $CDCl_3$. The mixture was left for two days to reach equilibrium before the equilibrium between the two species was determined by ^1H NMR (Figures S64-65).

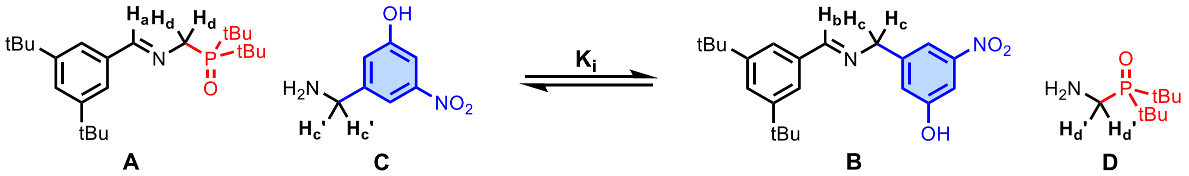

Figure S65. The equilibrium established by the mixing of **23** (**B**) with **14** (**D**) to yield imine **A** and amine **C**. The protons used to determine the equilibrium constant (K_i) are labelled.

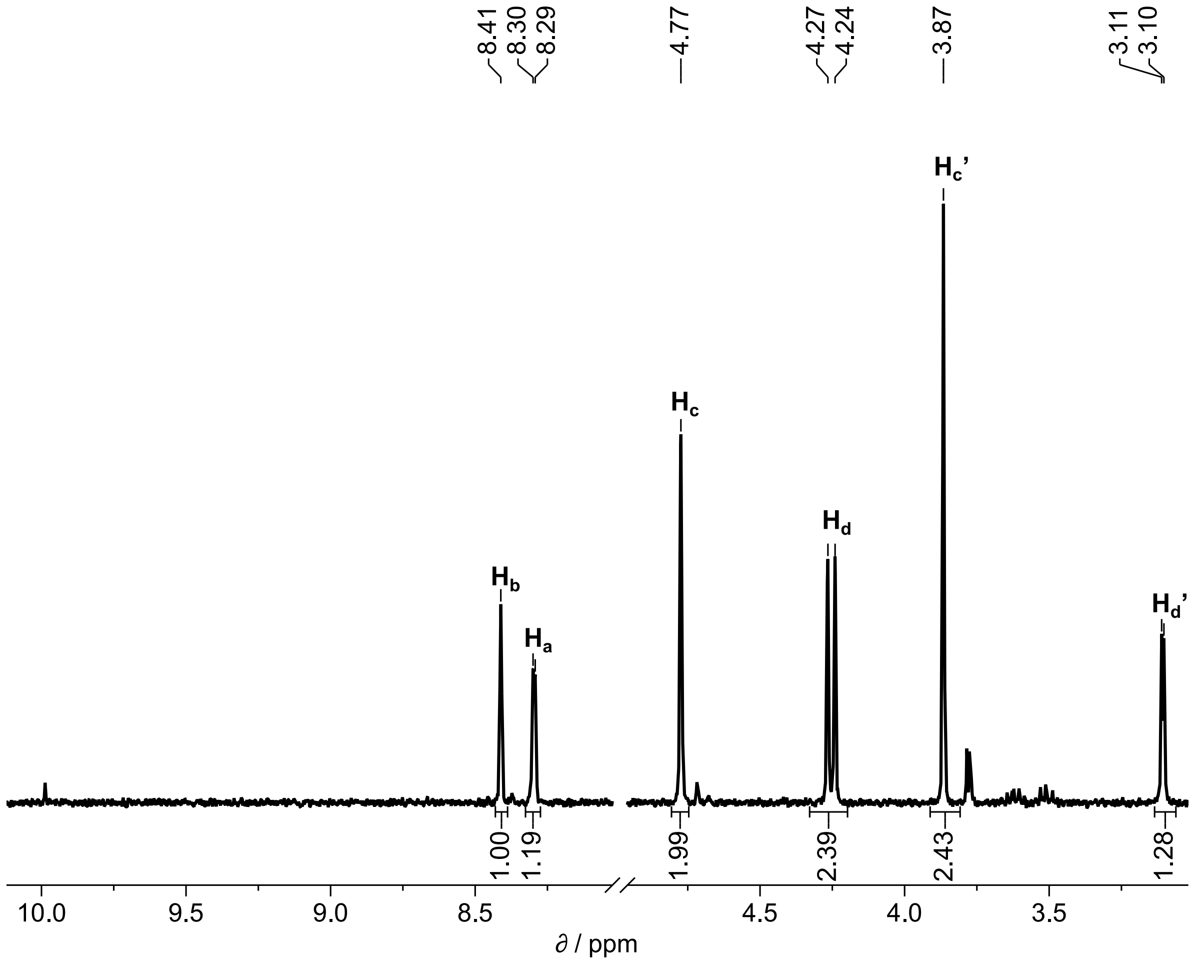

Figure S66. ^1H NMR spectrum (400 MHz, $CDCl_3$, 298 K) of the equilibrated imine mixture. The protons are labelled according to Figure S64.

The equilibrium constant is defined as:

$$K_i = \frac{[\boldsymbol{B}][\boldsymbol{D}]}{[\boldsymbol{A}][\boldsymbol{C}]}$$

so can be expressed as:

$$K_i = \frac{[I_b][I_{d'}]}{[I_a][I_{c'}]}$$

where I_x is the integral of proton x.

Evaluating the equation gives a value for K_i of 0.44, which indicates a slightly preference for the phosphine oxide side-chain to be incorporated into the imines.

Using this value, the stability constant of each composition of possible base-filling products onto a aldehyde trimer REMO can be calculated (relative to the **A3** strand).

$K_{\boldsymbol{A3}} = 1$

$K_{\boldsymbol{A2D}} = 3 \times 0.44$

$K_{\boldsymbol{AD2}} = 3 \times 0.44^2$

$K_{\boldsymbol{D3}} = 0.44^3$

These stabilities can be used to calculate the yield of each of these products in a base-filling experiment onto a trimeric blank strand in the absence of a template. These calculated yields are shown in Table S4.

d. Analysis of final copy mixture

The area of each of the copy strand peaks (254 nm) was integrated using Agilent Chemstation software. The obtained area integrals were used to calculate the yield of each copy (Table S4).

Table S4. The peak area and estimated yield for each product.

Product	Peak area (arbitrary units)	Expt. Yield (%)	Calc. Yield without template (%)
**CAD2**	2620	68	19
**CA2D**	954	25	44
**CD3**	289	7	3
**CA3**	0	0	33

e. Product sequencing

To the sample of purified **CAD2** in THF (1 mL), acetic anhydride (3 drops) was added and the solution was stirred at room temperature for 1 hour. The solvent was removed under a flow of nitrogen and the residue was redissolved in methanol (1 mL). The solution was stirred at room temperature overnight. The solvent was removed under a flow of nitrogen and the obtained residue was then subjected to the general sequencing procedure (see above) and analysed by UPLC-MS.

4. References

(1) Katritzky, A. R.; Oniciu, D. C.; Ghiviriga, I.; Barcock, R. A. 4,6-Bis- and 2,4,6-Tris-(N,N-Dialkylamino)-s-Triazines: Synthesis, NMR Spectra and Restricted Rotations. *J. Chem. Soc., Perkin Trans. 2* **1995**, No. 4, 785–792. https://doi.org/10.1039/P29950000785.

(2) Troselj, P.; Bolgar, P.; Ballester, P.; Hunter, C. A. High-Fidelity Sequence-Selective Duplex Formation by Recognition-Encoded Melamine Oligomers. *J. Am. Chem. Soc.* **2021**, *143* (23), 8669–8678. https://doi.org/10.1021/jacs.1c02275.

(3) Balduzzi, F.; Munasinghe, V.; Evans, O. N.; Lorusso Notaro Francesco, A.; Anderson, C. J.; Nigrelli, S.; Escobar, L.; Cabot, R.; Smith, J. T.; Hunter, C. A. Length and Sequence-Selective Polymer Synthesis Templated by a Combination of Covalent and Noncovalent Base-Pairing Interactions. *J. Am. Chem. Soc.* **2024**, *146* (47), 32837–32847. https://doi.org/10.1021/jacs.4c13452.

(4) Smith, J. T.; Baixeras Buye, J.; Iddon, B.; Soloviev, D. O.; Hunter, C. A. Template-Directed Synthesis of Recognition-Encoded Melamine Oligomers Using a Base-Filling Strategy. *J. Am. Chem. Soc.* **2025**, *147* (21), 18284–18294. https://doi.org/10.1021/jacs.5c05681.

(5) Cotirlan, A.-L.; Anderson, C. J.; Eyre, N. E. J.; Soloviev, D. O.; Iddon, B.; Balduzzi, F.; Hunter, C. A. Sequence-Dependent Folding of Recognition-Encoded Melamine Oligomers. submitted.
